# Targeted quantification of *N*-1-(carboxymethyl) valine and *N*-1-(carboxyethyl) valine peptides of β-hemoglobin for better diagnostics in diabetes

**DOI:** 10.1186/s12014-016-9108-y

**Published:** 2016-03-29

**Authors:** Mashanipalya G. Jagadeeshaprasad, Kedar B. Batkulwar, Nishita N. Meshram, Shalbha Tiwari, Arvind M. Korwar, Ambika G. Unnikrishnan, Mahesh J. Kulkarni

**Affiliations:** Proteomics Facility, Division of Biochemical Sciences, CSIR-National Chemical Laboratory, Pune, 411008 India; Academy of Scientific and Innovative Research (AcSIR), New Delhi, India; Department of Diabetes and Endocrine Research, Chellaram Diabetes Institute, Pune, Maharashtra India

**Keywords:** Diabetes, Diagnosis, Glycation, Glycemic control, HbA1c, Mass spectrometry

## Abstract

**Background:**

*N*-1-(Deoxyfructosyl) valine (DFV) β-hemoglobin (β-Hb), commonly referred as HbA1c, is widely used diagnostic marker in diabetes, believed to provide glycemic status of preceding 90–120 days. However, the turnover of hemoglobin is about 120 days, the DFV-β-Hb, an early and reversible glycation product eventually may undergo irreversible advanced glycation modifications such as carboxymethylation or carboxyethylation. Hence quantification of *N*-1-(carboxymethyl) valine (CMV) and *N*-1-(carboxyethyl) valine (CEV) peptides of β-Hb would be useful in assessing actual glycemic status.

**Results:**

Fragment ion library for synthetically glycated peptides of hemoglobin was generated by using high resolution–accurate mass spectrometry (HR/AM). Using parallel reaction monitoring, deoxyfructosylated, carboxymethylated and carboxyethylated peptides of hemoglobin were quantified in clinical samples from healthy control, pre-diabetes, diabetes and poorly controlled diabetes. For the first time, we report *N*-1-β-valine undergoes carboxyethylation and mass spectrometric quantification of CMV and CEV peptides of β-hemoglobin. Carboxymethylation was found to be the most abundant modification of *N*-1-β-valine. Both CMV-β-Hb and CEV-β-Hb peptides showed better correlation with severity of diabetes in terms of fasting glucose, postprandial glucose and microalbuminuria.

**Conclusions:**

This study reports carboxymethylation as a predominant modification of *N*-1-β-valine of Hb, and quantification of CMV-β-Hb and CEV-β-Hb could be useful parameter for assessing the severity of diabetes.

**Electronic supplementary material:**

The online version of this article (doi:10.1186/s12014-016-9108-y) contains supplementary material, which is available to authorized users.

## Background

Poorly controlled diabetes leads to variety of complications including blindness, amputations and, kidney failure [1]. Thus, glycemic control is crucial in management of diabetes [2]. Diagnosis and management of diabetes heavily relies on detection of levels of HbA1c [*N*-1-(deoxyfructosyl) valine (DFV) β-hemoglobin], which is considered as a gold standard for assessing the glycemic status over preceding 90–120 days [3, 4]. In normal individuals, HbA1c ranges from 3 to 6.5 % and up to 15 % in poorly controlled diabetes [5]. However, previous studies have suggested that the HbA1c is slowly reversible, and for a given glucose concentration it eventually reaches equilibrium [6]. Furthermore precise quantification of HbA1c using available methods in routine diagnostics, such as ion exchange [7], phenylboronate affinity [8] or antibody based chromatography [9], are technically challenging. For example, ion exchange based measurement of HbA1c is influenced by other chemical modifications such as glutathionylation [10]. Whereas boronate affinity chromatography invariably detects the *cis*-diol groups of glycated hemoglobin not limited to DFV but also includes other amino acids like lysine and arginine, which may result in overestimation of HbA1c [11]. In case of antibody based affinity chromatography method, lack of specificity against DFV peptide poses serious problems.

Limitations in the light of current methods to measure accurate HbA1c, we explored the chemistry of HbA1c. HbA1c is chemically *N*-1-(deoxyfructosyl) valine (DFV) β-hemoglobin, an early and reversible glycated product formed by non-enzymatic reaction with glucose, can possibly undergo relatively stable advanced glycation modifications such as CMV and CEV during the lifespan of erythrocytes. Therefore, the assumption that HbA1c provides glycemic status over preceding 90–120 days is debatable. In fact, elevated levels of CMV-Hb were observed in diabetic nephropathic patients by immunosassy in 2001 [12]. However, its usefulness in diagnostics has been overlooked. Advanced mass spectrometry based quantitative approaches like MRM/PRM may provide better view of glycation status of hemoglobin. In MRM, a specific precursor and fragment ions are monitored for quantitation using triple quadrupole mass spectrometers. MRM based quantification is becoming quite popular in the area of proteomics research, as this approach is able to replace expensive antibody-based quantifications like western blotting and ELISA [13]. MRM performed on high resolution mass spectrometer is called parallel reaction monitoring (PRM) or HR-MRM. Unlike MRM, in PRM it is not possible to monitor the specific fragment ion during acquisition, as third quadrupole is replaced with high resolution mass analyzers such as Orbitraps and Time of flight (TOF). In PRM, post mass spectral acquisition, extracted ion chromatograms (XICs) for selected fragment ions are used for quantitation [14]. Hence, we explored the existence of CMV and CEV modifications for possibility of discovering alternative diagnostics in diabetes using PRM. In this pursuit, for the first time, we have comprehensively characterized and quantified DFV/DFL, CMV/CML and CEV/CEL peptides of hemoglobin. The major findings of this study are *N*-1-β-valine undergoes carboxyethylation; and carboxymethylation is the predominant modification of *N*-1-β-val-Hb. Furthermore, CMV and CEV peptides correlate well with fasting glucose, postprandial glucose, and microalbuminuria. Thus, we propose that CMV-β-Hb and CEV-β-Hb provides better insight of glycemic status in diabetes.

## Methods

All the chemicals were procured from Sigma-Aldrich (Sigma-Aldrich, MO, USA). MS grade solvents (Acetonitrile and Water) were obtained from J T. Baker (J T. Baker, PA, USA). RapiGest SF was procured from Waters (Waters Corporation, MA, USA). Membrane filters of 3 kDa cut off were procured from Millipore (Millipore, MA, USA).

### Study design and clinical details

**Fig. 1:**
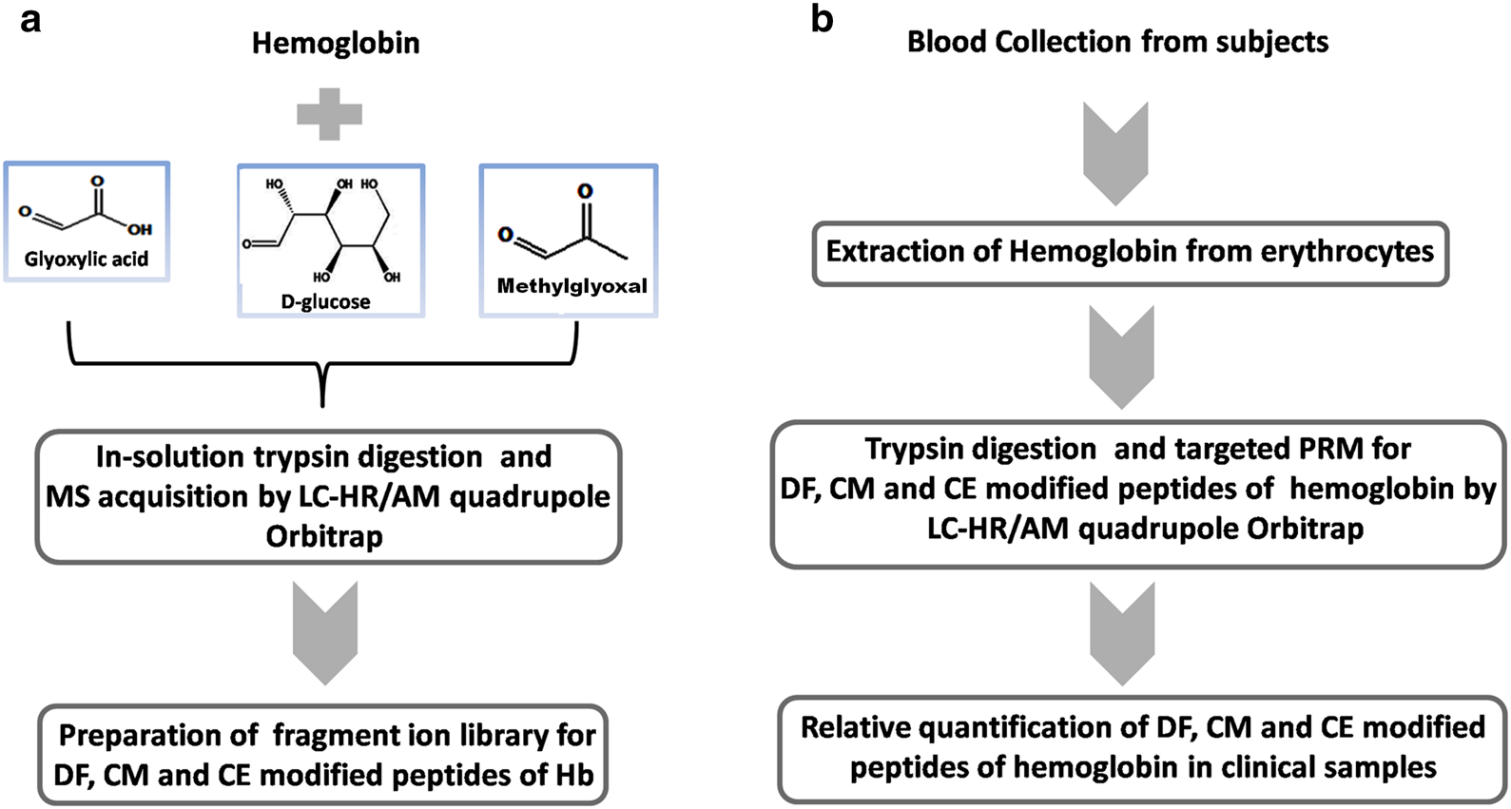
Overview of experimental design. **a** In-vitro synthesis of DF, CM and CE modified hemoglobin and fragment ion library preparation for glycated peptides. **b** Quantific**a**tion of glycated peptides in clinical samples

The current study deals with the characterization and quantification of DFV/DFL, CMV/CML and CEV/CEL modified peptides of hemoglobin from 39 clinical samples. The overview of the study design is depicted in Fig. 1a, b. The study was approved by Chellaram Diabetes Institute Ethics Committee (CDIEC), Pune, India. The written consent was obtained from all subjects prior to blood collection. Blood samples were collected from Chellaram Diabetes Institute (CDI), Pune and grouped into healthy controls (n = 9), pre-diabetes (n = 10), diabetes (n = 10) and poorly controlled diabetes (n = 10) based on their fasting, postprandial blood glucose levels and HbA1c. The descriptive characters and biochemical parameters of the study subjects are provided in Additional file 1: Table S1.

### Synthesis of AGE modified hemoglobin

#### Chemical synthesis of carboxymethyl and carboxyethyl modified hemoglobin

Hemoglobin (10 mg/mL) and sodium cyanoborohydride (0.05 M) were dissolved in 0.2 M sodium phosphate buffer (pH 7.4), to which either glyoxylic acid (0.045 M) or methylglyoxal (0.05 M) were added [15, 16], the reaction mixture was incubated at 37 °C for 24 h under sterile and dark conditions to obtain carboxymethyl and carboxyethyl modified hemoglobin respectively.

#### Synthesis of AGE modified hemoglobin

Hemoglobin (10 mg/mL) and glucose (0.5 M) in 0.2 M sodium phosphate buffer (pH 7.4) were incubated at 37 °C for 15 days under sterile and dark conditions [15, 16].

### Hemoglobin extraction from clinical samples

Blood was collected from subjects in heparin-coated capillary tubes containing aprotinin (BD Bioscience). The blood samples were kept at 4 °C for 15 min, and centrifuged at 3000 rpm for 15 min to remove plasma. The pellet containing erythrocytes was washed twice with saline buffer and stored at −80 °C until use. Erythrocytes were lysed in ammonium bicarbonate buffer (50 mM) containing 0.1 % RapiGest SF with vigorous vortexing for 30 min at 4 °C [17]. Further, cell lysate was centrifuged at 20,000 rpm for 30 min and protein in the supernatant was collected.

### Sample preparation and LC–MS/MS acquisition

In vitro AGE modified hemoglobin and hemoglobin isolated from clinical samples, were diluted with 50 mM ammonium bicarbonate buffer containing 0.1 % RapiGest SF followed by reduction (Dithiothritol), alkylation (Iodoacetamide) and trypsin digestion. Tryptic peptides were loaded onto reverse-phase column (150 × 2.1 mm, 1.9 µm) on an UPLC Accela 1250 (Thermo Fisher Scientific) coupled to a Q-Exactive Orbitrap mass spectrometer (Thermo Fisher Scientific) at a flow rate of 350 µL/min over a period of 45 min gradient, using 6 gradient segments (held at 2 % solvent A over 2 min, 2–40 % A over 35 min, 40–98 % A over 2 min, held at 98 % A over 2 min, 98–2 % A over 2 min held at 2 % A for 2 min). Solvent A was ACN with 0.1 % formic acid and solvent B was aqueous with 0.1 % formic acid.

### Quadrapole-Orbitrap mass spectrometry instrument conditions

Hybrid quadruple Q-Exactive Orbitrap was used to perform MS and MS/MS with resolution of 70,000 (MS at *m*/*z* 400), 17,500 (MS/MS at *m*/*z* 400), AGC target of 1e^6^ (MS) and 1e^5^ (MS/MS) using HESI source. The mass spectra of eluted peptides were acquired in a positive mode in data dependent manner with a mass range of 350–2000 *m*/*z* and scan time of 120 ms. MS acquisition was performed at precursor’s ion selection width of 2 *m*/*z*, under fill ratio of 0.3 % and with dynamic exclusion time of 15 s. The peptides fragmentation was performed with high energy collision induced dissociation (HCD) of 28 eV and MS/MS scans were acquired with starting *m*/*z* of 50.

### Identification of glycation modifications

Mass spectra were analyzed by using Proteome Discover software (PD 1.4.0.288, Thermo Fisher Scientific). Sequest-HT was used as a search engine for peptide identification against protein database consisting of α and β Hb (UniProt IDs: P68871 and P69905). Peptide and fragment mass tolerance was set at 10 ppm and 0.5 Da respectively. DFV/DFL (+162.0211 Da), CMV/CML (+58.0055 Da) and CEV/CEL (+72.0211 Da) were given as variable modifications at N-terminal valine and lysine residues [15, 16, 18]. The false positive rate was set to 1 % in the PD workflow. Glycated peptides were manually validated for accurate mass shift in precursor ion due to modification, and presence of fragments retaining modification. Subsequently, fragment ion library was generated for DFV/DFL, CMV/CML, and CEV/CEL modified hemoglobin peptides (Additional file 2: Table S2).

### Parallel reaction monitoring (PRM) data acquisition

A total of 42 *m*/*z* corresponding to DFV/DFL, CMV/CML and CEV/CEL modified peptides observed in vitro were used for PRM analysis in clinical samples (Additional file 2: Table S2). PRM was performed at a resolution of 17,500, isolation window of 2 Da, target AGC value of 2e^5^, maximum injection time of 150 ms, MSX count 1, and isolation offset 0.5 *m*/*z*. Fragmentation of precursor peptides was performed by high energy collision induced dissociation (HCD) of 28 eV.

### Quantification of glycated peptides

Raw files obtained from PRM acquisitions were analyzed by Pinpoint software (version 1.4.0 Thermo Fisher Scientific). Ion chromatograms were extracted with a mass tolerance of 10 ppm using all b and y ions. The composite MS/MS spectrum of each targeted peptide was reconstructed from the area under curves (AUCs) of all the transitions (Fig. 4b, Additional file 3). The AUC for each modified peptide was deduced by addition of corresponding common co-eluted fragment ions AUCs observed in all replication and groups [14].

### Statistical analysis

Mass spectra for clinical samples were acquired in technical triplicates. Graphpad Prism 5 ver 5.01 was used to determine statistical significance. Two-way ANOVA followed by Tukey’s test and Bonferroni posttests were performed for the AUC of quantified glycated peptides and their fold change in prediabetes, diabetes and poorly controlled diabetes. The AUCs of the modified peptides were correlated with clinical parameters blood glucose, HbA1c, microlabuminuria, etc. by Pearson’s correlation method. p values less than 0.05 were considered as significant (Additional file 4).

## Results and discussion

### Construction of fragment ion library for synthetically glycated hemoglobin peptides

**Fig. 2:**
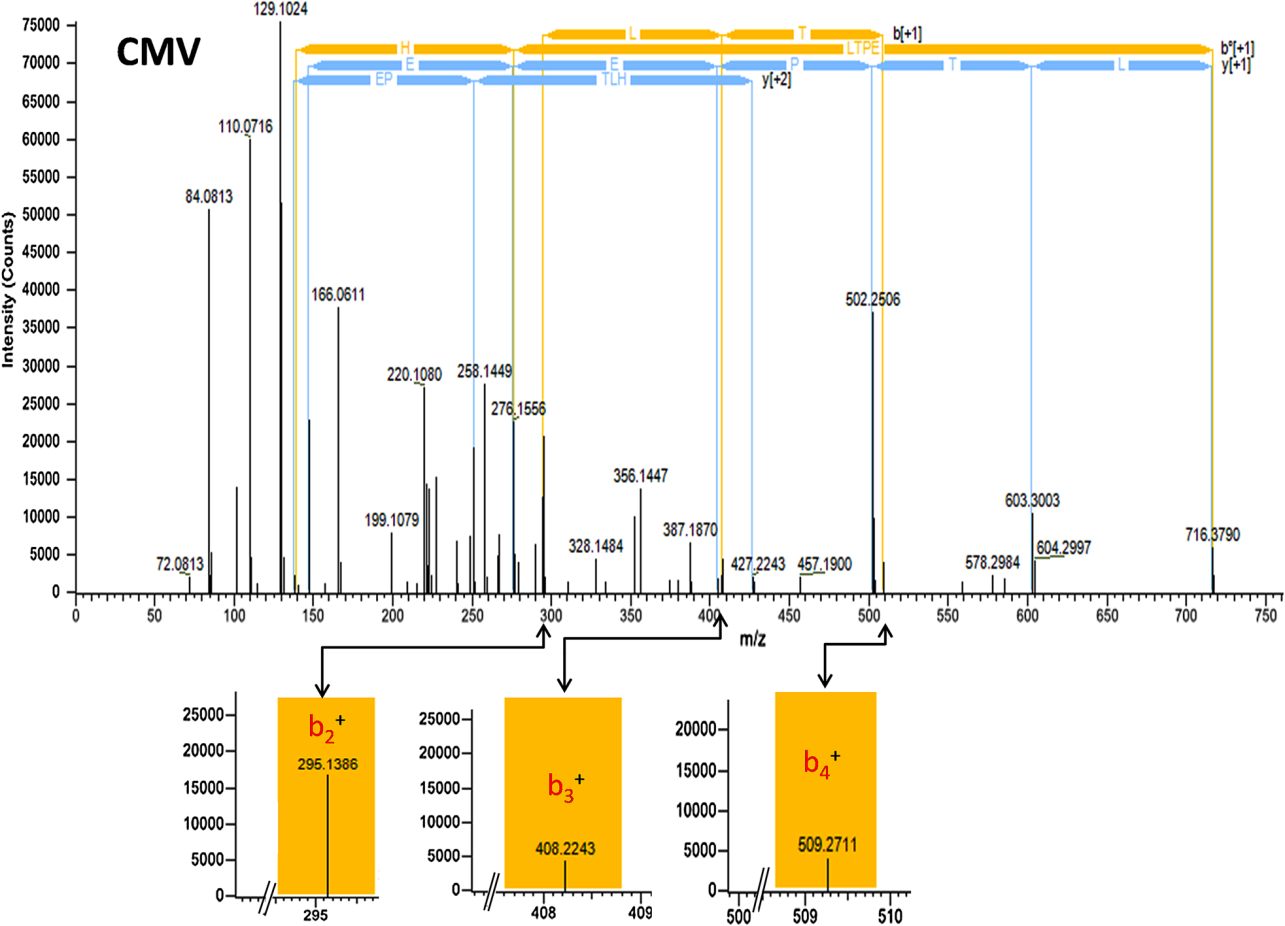
MS/MS annotation for CMV (*m*/*z*-1010.5116 Da) peptide of β-Hb depicting modified fragment ions

Mass-spectrometry based targeted quantification approaches like MRM and PRM heavily relies on fragment ion or spectral library [14, 16]. Therefore, fragment ion library for synthetically glycated hemoglobin peptides was constructed. DFV/DFL, CMV/CML and CEV/CEL containing peptides of hemoglobin were studied by LC–MS/MS analysis. Initially the AGE modified peptides identified by Proteome Discoverer were analyzed for exact mass increment of 162.0211 Da (DFV/DFL), 58.0055 Da (CMV/CML) and 72.0211 Da (CEV/CEL) in the precursor ions. Subsequently, the MS/MS spectra were manually inspected for presence of modified fragment ions as described earlier [16, 18]. For example, the N-terminal peptide of β-Hb “VHLTPEEK” was interrogated for deoxyfructosyl (DF), carboxymethyl (CM), and carboxyethyl (CE) modifications involving *N*-1-valine. The precursor mass of unmodified “VHLTPEEK” was 952.5078 Da, while DF-VHLTPEEK (1114.5607 Da), CM-VHLTPEEK (1010.5116 Da) and CE-VHLTPEEK (1024.5297 Da) peptides showed mass increment of 162.0211, 58.0055, 72.0211 Da respectively. Further, these peptides were manually inspected for modified fragment ions (e.g. CM modified V*HLTPEEK, *m*/*z*-505.75, b_2_^+^-295.1400, b_3_^+^-408.2241, b_4_^+^-509.2718) and were considered to be truly modified if the MS/MS spectrum showed presence of corresponding modified fragment ions. The annotated MS/MS spectrum for CM-VHLTPEEK is represented in Fig. 2. The list of all modified peptides and their corresponding modified fragment ions are listed in Additional file 2: Table S2 and their MS/MS annotations are provided in Additional file 3.

A total of 42 glycated peptides (Additional file 2: Table S2) were identified in DDA analysis. Glyoxylic acid modified hemoglobin had maximum number of glycated peptides (20), followed by glucose modified (13) and methylglyoxal (9). Irrespective of glycating agent used, 26 peptides were observed to be carboxymethylated. Together these glycated peptides involved 19 sites, 9 from α-Hb and 10 from β-Hb (Additional file 2: Table S2). In case of glucose modified hemoglobin, apart from deoxyfructosylation of *N*-1-β-Val, it was interesting to observe carboxymethylation and carboxyethylation of *N*-1-β-Val and other lysine residues.

### Quantification of advanced glycated peptides of hemoglobin in clinical samples

**Fig. 3:**
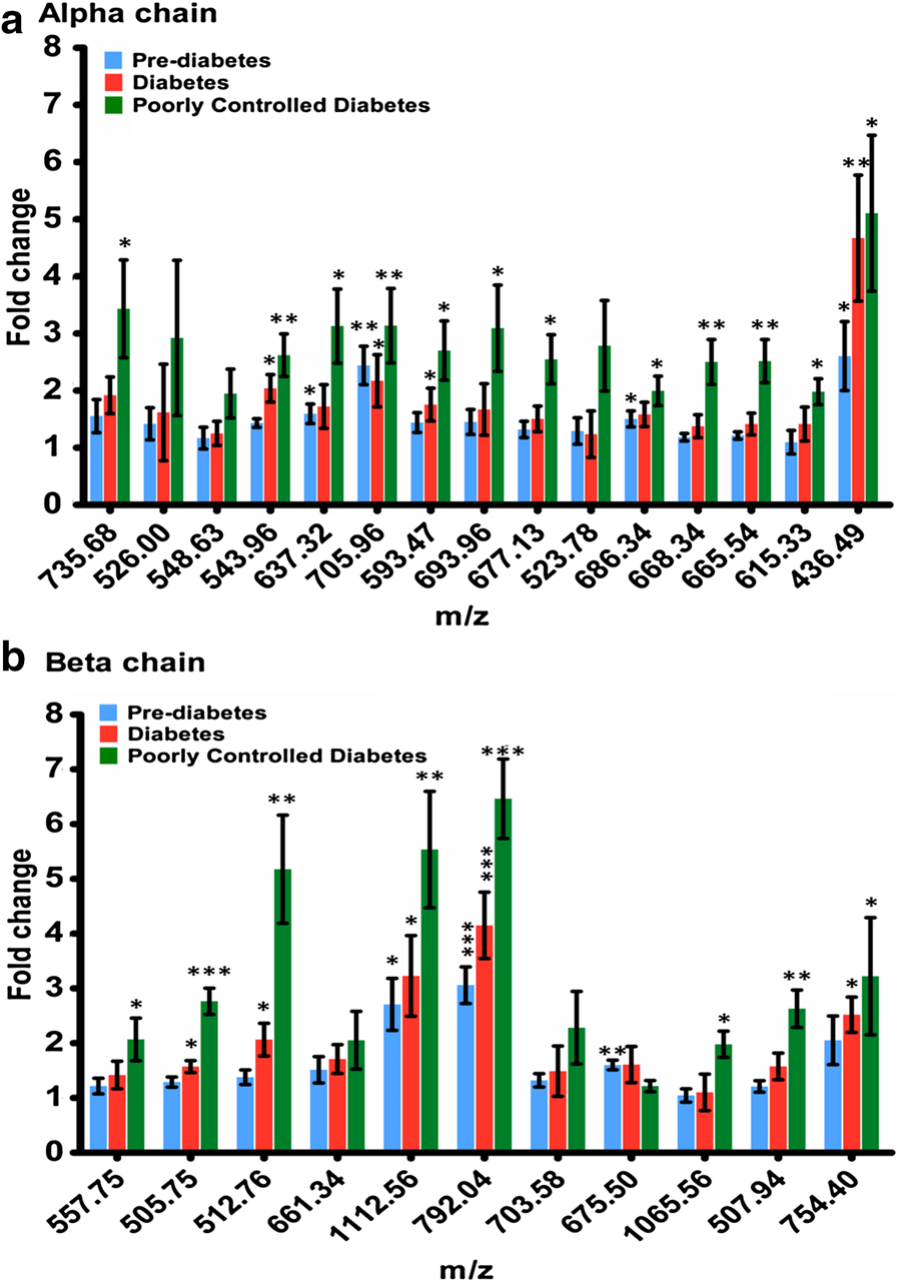
**a** Relative fold change in AUC for glycated peptides of **a** α-Hb and **b** β-Hb with respect to healthy control. Statistical analysis was performed by two-way ANOVA followed by Tukey’s test. PD-prediabetes, D-diabetes and PCD-poorly controlled diabetes (*p < 0.05, **p < 0.005, ***p < 0.0005)

Using precursor ion information, DFV/DFL, CMV/CML, and CEV/CEL modified hemoglobin peptides from Table S2 were quantified by PRM followed by PINPOINT analysis in clinical samples. Both b and y ions from all the transitions were used to generate the composite MS/MS spectrum for each targeted peptide. The co-eluted fragment ions of the glycated precursor were reconfirmed for the presence of fragment ions observed in the synthetically glycated hemoglobin. For relative quantification of modified peptides, the total AUCs of corresponding common co-eluted fragment ions observed in all replication and groups were used. The list of modified peptides and their corresponding fragment ions used for quantification is mentioned in Table 1. Total 26 glycated peptides (15 from α-Hb and 11 from β-Hb) were identified and quantified in clinical samples (Fig. 3a, b). Fold change in AUCs was calculated for all the modified peptides across different clinical conditions and is represented in Fig. 3a, b. A total of 13 peptides of α-Hb and 9 peptides of β-Hb were significantly elevated in poorly controlled diabetes as depicted in Fig. 3a, b respectively. Glycated peptides of α-Hb (1) sequence: K*(CM)VADALTNAVAHVDDM*(Oxd)PNALSALSDLHAHK*(CM)LR, *m*/*z*-705.96, site-K61 and K90; (2) sequence: K*(CM)VADALTNAVAHVDDMPNALSALSDLHAHK, *m*/*z*-640.12, site-K61; and β-Hb (3) sequence: V*(CM)HLTPEEK*(CM)SAVTALWGK*(CM)VNVDEVGGEALGR, *m*/*z*: 1112.56, site-V1, K8 and K17 and (4) FFESFGDLSTPDAVM*(Oxd)GNPK*(CEL)VK, /z: 792.04, site-K61 showed significant increase in all the diabetic conditions. Thus, these sites perhaps can be considered as glycation sensitive.

**Table 1:** The list of modified peptides and their corresponding fragment ions used for quantification in clinical samples

Sl. N	Mod site	Peptide sequence	Peptide MH + Da	Monoisotopic *m*/*z* Da (mmu/ppm)	CS	Mod	Fragment ions used for quantification		
*Alpha chain of hemoglobin*									
1	K 7 and K 11	VLSPADKTNVK*AAWGK	1741.0936	436.491 Da (−0.49 mmu/−1.02 ppm),	4	CML	204.133 (y_2_^+^)	390.2130 (y_3_^+^)	
2	K 7	VLSPADK*TNVK	1229.67131	615.33929 Da (−1.17 mmu/−1.9 ppm),	2	CML	213.1592 (b_2_^+^)	300.1912 (b_3_^+^)	769.4085 (b_7_^+^)
3	K 16	AAWGK*VGAHAGEYGAEALER	2205.04978	735.68811 Da (−2.47 mmu/−3.36 ppm),	3	DFL	329.1602 (b_3_^+^)	386.1817 (b_4_^+^)	417.2450 (y_3_^+^)
4	K 16	AAWGK*VGAHAGEYGAEALER	2101.01455	526.00909 Da (+1.18 mmu/+ 2.24 ppm),	4	CML	386.1817 (b_4_^+^)	572.2821 (b_5_^+^)	688.3618 (y_6_^+^)
5	K 61	K*VADALTNAVAHVDDMPNALSALSDLHAHK	3182.58649	637.32312 Da (−0.71 mmu/−1.11 ppm)	5	CML	286.1755 (b_2_^+^)	357.2127 (b_3_^+^)	720.3781 (y_6_^+^)
6	K 90	VADALTNAVAHVDDMPNALSALSDLHAHK*LR	3427.71986	686.34979 Da (−1.55 mmu/−2.26 ppm)	5	DFL	578.3185 (y_3_^+^)	715.3774 (y_4_^+^)	923.4734 (y_6_^+^)
7	K 90	KVADALTNAVAHVDDMPNALSALSDLHAHK*LR	3555.81860	593.47583 Da (−0.66 mmu/−1.12 ppm),	6	DFL	578.3185 (y_3_^+^)	715.3774 (y_4_^+^)	786.4145 (y_5_^+^)
8	K 40 and K 56	MFLSFPTTK*TYFPHFDLSHGSAQVK*GHGK	3381.6435	677.13452 Da (+1.46 mmu/+ 2.15 ppm)	5	CML and CML	626.3001 (b_5_^+^)	811.4415 (y_7_^+^)	
9	K 61 and K 90	K*VADALTNAVAHVDD**M**PNALSALSDLHAHK*LR	3525.7761	705.96106 Da (+0.11 mmu/+ 0.16 ppm)	5	CML, OXD and CML	175.118 (y_1_^+^)		
10	K 90	VADALTNAVAHVDDMPNALSALSDLHAHK*LR	3323.6833	665.54248 Da (+0.61 mmu/+ 0.91 ppm)	5	CML	932.541 (y_7_^+^)	1047.568 (y_8_^+^)	
11	K 139	FLASVSTVLTSK*YR	1629.8869	543.96716 Da (+0.75 mmu/+ 1.37 ppm)	3	CML	175.1184 (y_1_^+^)	611.3147 (y_4_^+^)	605.3287 (b_6_^+^)
12	K 11	TNVK*AAWGK	1046.5608	523.78406 Da (−1.07 mmu/−2.04 ppm)	2	CEL	515.2817 (b_4_^+^)	586.3189 (b_5_^+^)	732.4033 (y_6_^+^)
13	K 90	VADALTNAVAHVDDMPNALSALSDLHAHK*LR	3337.6939	668.34460 Da (−0.4 mmu/−0.6 ppm)	5	CEL	833.4734 (y_6_^+^)	1061.5844 (y_8_^+^)	
14	K 90	KVADALTNAVAHVDDMPNALSALSDLHAHK*LR	3465.7984	693.96552 Da (+1.52 mmu/+ 2.19 ppm)	4	CEL	625.3774 (y_4_^+^)	833.4734 (y_6_^+^)	
15	K 139	FLASVSTVLTSK*YR	1643.9034	548.63934 Da (+1.04 mmu/+ 1.9 ppm),	3	CEL	175.1184 (y_1_^+^)	938.5299 (y_7_^+^)	518.2967 (b_5_^+^)
*Beta chain of hemoglobin*									
16	V 1	V*HLTPEEK	1114.56072	557.78400 Da (−0.99 mmu/−1.77 ppm)	2	DFV	1095.511 (b_8_^+^)	853.4408 (y_7_^+^)	
17	V 1	V*HLTPEEK	1010.51164	505.75946 Da (−1.85 mmu/−3.66 ppm)	2	CMV	295.1317 (b_2_^+^)	605.3162 (b_5_^+^)	
18	V 1 and K 8	V*HLTPEEK*SAVTALWGK	1982.0245	661.34637 Da (+0.52 mmu/+ 0.78 ppm)	3	CMV and CML	295.1395 (b_2_^+^)	147.1122 (y_1_^+^)	204.1337 (y_2_^+^)
19	V 1, K 8 and K 17	V*HLTPEEK*SAVTALWGK*VNVDEVGGEALGR	3335.6835	1112.56604 Da (+2.67 mmu/+ 2.4 ppm)	3	CMV, CML and CML	295.1395 (b_2_^+^)	659.3465 (y_7_^+^)	
20	K 59 and K 61	FFESFGDLSTPDAV**M**GNPK*VK*AHGK	2811.3238	703.58643 Da (−1.42 mmu/−2.02 ppm)	4	OXDN, CML and CML	511.2181 (b_4_^+^)	658.2866 (b_5_^+^)	204.1337 (y_2_^+^)
21	K 66 and K 82	K*VLGAFSDGLAHLDNLK*GTFATLSELH**C**DK	3373.6704	675.53992 Da (−0.59 mmu/−0.88 ppm)	5	CML, CML and CABD	187.1071 (b_1_^+^)	286.1755 (b_2_^+^)	527.3182 (b_5_^+^)
22	K 120	LLGNVLVCVLAHHFGK*EFTPPVQAAYQK	3194.6810	1065.56519 Da (−0.35 mmu/−0.33 ppm),	3	CABD And CML	284.1963 (b_3_^+^)	497.3076 (b_5_^+^)	
23	V 1	V*HLTPEEK	1024.5297	512.76849 Da (−0.64 mmu/−1.25 ppm)	2	CEV	522.2790 (b_4_^+^)	276.1548 (y_2_^+^)	
24	K 59	FFESFGDLSTPDAV**M**GNPK*VK	2374.1282	792.04761 Da (+0.33 mmu/+ 0.42 ppm)	3	OXD and CEL	658.2866 (b_5_^+^)	715.3080 (b_6_^+^)	
25	K 144	VVAGVANALAHK*YH	1521.8168	507.94379 Da (−0.16 mmu/−0.32 ppm)	3	CEL	199.1435 (b_2_^+^)		
26	K 144	VVAGVANALAHK*YH	1507.79827	754.40277 Da (−1.69 mmu/−2.24 ppm)	2	CML	505.2399 (y_3_^+^)	826.4201 (y_6_^+^)

**Fig. 4:**
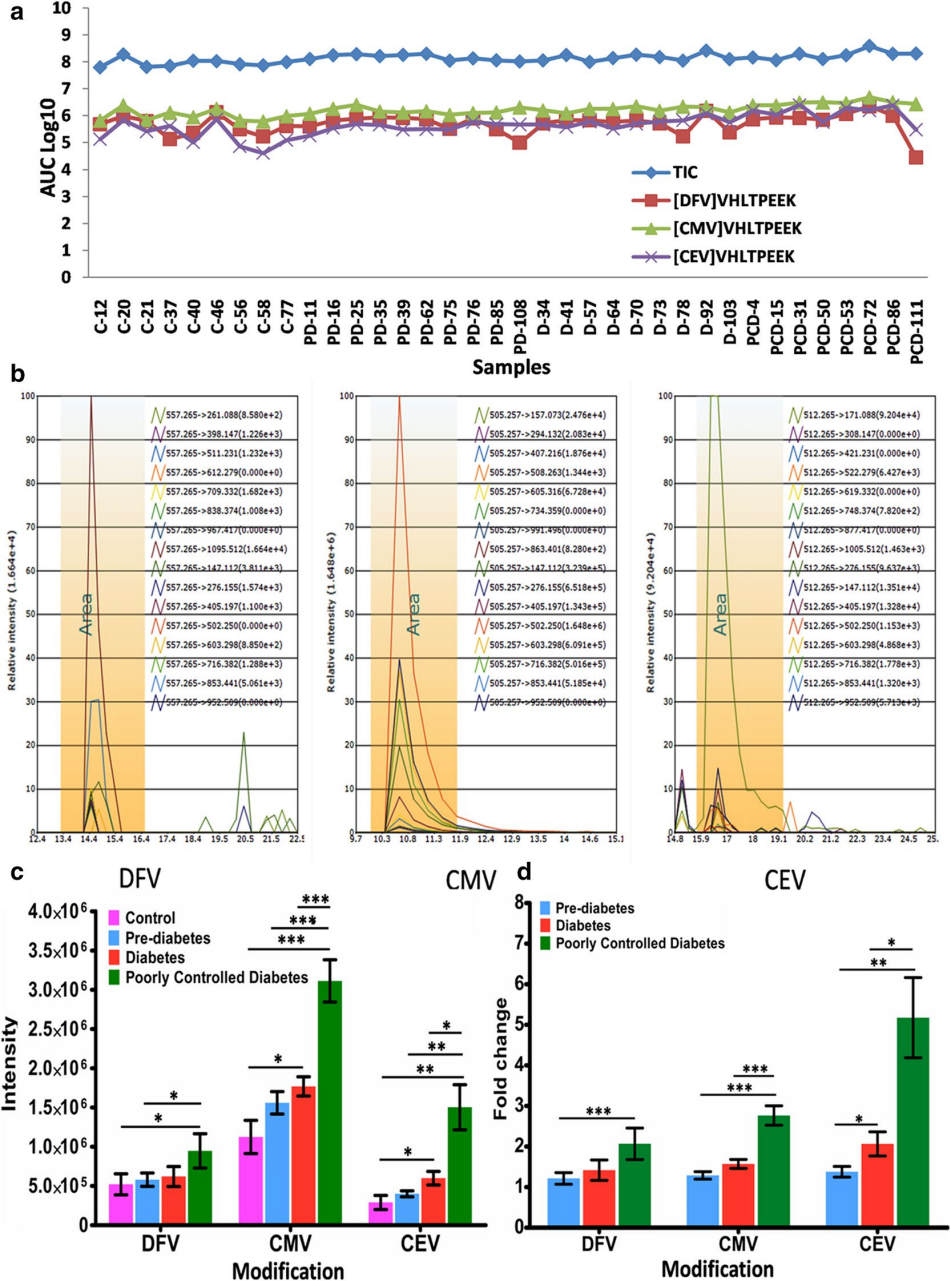
**a** Displays the Log_(10)_ values of average of TIC and average of AUC of CMV, CEV and DFV peptides, indicating that there was no major variation in TIC across different samples, although the AUC of CMV, CEV and DFV increased with severity of diabetes. **b** Spectra depicting co-eluted fragment ions of DFV, CMV and CEV peptides of β-hemoglobin using PINPOINT software. **c** AUC of DFV, CMV and CEV peptides of β-hemoglobin depicting relative abundance. **d** Relative fold change in AUC for DFV, CMV and CEV peptides of β-hemoglobin by PRM. Statistical analysis was performed by two-way ANOVA followed by Tukey’s test and Bonferonnis posttests. Clinical groups are represented as *C* control, *PD* prediabetes, *D* diabetes, *PCD* poorly controlled diabetes (*p < 0.05, **p < 0.005, ***p < 0.0005)

In context of diagnostics of diabetes, DFV of β-Hb is quantified. Therefore, here we have emphatically discussed the quantification of advanced glycated peptides involving *N*-1-β-Val. Figure 4a displays the Log_(10)_ values of average TIC, and average AUC of CMV, CEV and DFV peptides, indicating that there was no major variation in TIC across different samples, although the AUC of CMV, CEV and DFV increased with severity of diabetes. Subsequently, quantification of CMV, CEV and DFV peptides was performed by PINPOINT analysis. For the first time, we have observed that *N*-1-β-valine undergoes carboxyethylation; and carboxymethylation as a predominant modification of *N*-1-β-Val in all the subjects (Fig. 4b, c). Apart from V*(CM)HLTPEEK (*m*/*z*-505.75), an additional peptide with 2 missed cleavages V*(CM)HLTPEEK*(CM)SAVTALWGK*(CM)VNVDEVGGEALGR (*m*/*z*-1112.56) involving CMV modification was found to be significantly elevated in all the diabetic conditions.

Although previous studies have reported that CMV-Hb was more prevalent in diabetic patients than in healthy subjects, and was proposed to be a valuable marker for the progression of diabetic nephropathy, and oxidative stress [13, 19], this study reports that CMV is the predominant modification of β-*N*-1-Val in all the clinical conditions (Fig. 4c). Furthermore, CEV showed higher fold change in AUC followed by CMV and DFV, in all diabetic conditions compared to healthy controls (Fig. 4d, Additional file 5) suggesting its usefulness as marker in the early stages of diabetes. Thus, monitoring CMV and CEV, along with DFV could be useful to assess the glycemic status in diabetes.

### Correlation of DFV, CMV and CEV modified peptides of β-Hb with severity of diabetes

Next we correlated the levels of the modified peptides with clinical parameters by using Pearson’s correlation method (Table 2, Additional file 4). However, in the context of HbA1c, glycated peptides involving β-*N*-1-Val are discussed here. All three DFV, CMV and CEV peptides showed significant positive correlation with each other. However, CMV and CEV peptides showed significant correlation with HbA1c (r = 0.729*** and r = 0.549***), fasting blood glucose (r = 0.515** and r = 0.304*) and postprandial blood glucose (r = 0.696*** and r = 0.673***). While DFV peptide showed significant positive correlation with postprandial glucose (r = 0.284*) and HbA1c (r = 0.283*). Interestingly, the CMV peptide also showed a relatively better correlation with microalbuminuria (r = 0.305) and negative correlation with HDL (r = −0.362), although both were statistically not significant. This study suggests that CMV and CEV are better correlated with severity of diabetes.

**Table 2:** Correlations of glycated peptides of Hb with fasting blood glucose, postprandial blood glucose, HbA1c, serum triglyceride, LDL, HDL, cholesterol, VLDL, creatinine and microalbuminuria by Pearson’s correlation method

Sl. No.	Chain, glycation site and type of modification	Precursor *m*/*z*	Fasting blood glucose	Postprandial blood glucose	HbA1c	HDL	MIC
1	α-K-11-CML	436.491	0.423*	0.474**	0.569***	−0.3514	0.126
2	α-K-90-CML	665.54	0.411*	0.499**	0.546***	−0.326	−0.0284
3	α-K-90-CEL	668.34	0.406	0.515	0.554	−0.338	−0.0323
4	β-V-1-DFV	557.75	−0.05	0.284*	0.283*	−0.317	0.132
5	β-V-1-CMV	505.75	0.515**	0.696***	0.729***	−0.362	0.305
6	β-V-1-CEV	512.75	0.304*	0.673***	0.549***	−0.332	0.089
7	β-V-1-CMV, K-8-CML and K-17	1112.56	0.384*	0.475**	0.540***	−0.383	0.093
8	β-K-61-CEL	792.04	0.376*	0.657***	0.692***	−0.375	0.225
9	β-K-144-CML	754.4	0.379*	0.569***	0.650***	−0.320	0.229

* p < 0.05; ** p < 0.005; *** p < 0.0005

**Fig. 5:**
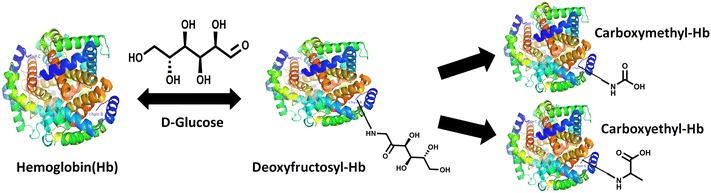
Mechanism of formation of CMV and CEV β-hemoglobin during dynamic glycation reaction

Proteins undergo heterogeneous modifications during dynamic advanced glycation reactions [20]. Generally lysine residues of protein reacts with glucose to form fructosyl lysine, which eventually undergo structural rearrangement involving fragmentation and glyoxidation to form AGEs like carboxymethyl lysine (CML) and carboxyethyl lysine (CEL). The occurrence of CML and CEL is accelerated in hyperglycemic conditions. Especially long lived proteins are known to have elevated levels of CML modifications [21]. Therefore, these AGE modifications may reflect the glycemic status over a long time. Despite this fact, HbA1c i.e. DFV-β-Hb, an early glycated product is used for diagnosis of diabetes. Furthermore, given the turnover of hemoglobin is 120 days, it is expected that DFV-β-Hb, the first and reversible modification of glycation, to undergo stable AGE modifications like carboxymethylation and carboxyethylation (Fig. 5). Moreover, elevated levels of dicarbonyl such as glyoxal and methylglyoxal can also cause carboxymethylation and carboxyethylation [20]. Our observation of carboxymethylation and carboxyethylation of *N*-1-β-valine supports the hypothesis that hemoglobin undergoes advanced glycation modifications.

## Conclusion

This is the first study that describes detailed characterization and quantification of advanced glycated peptides of hemoglobin including CMV and CEV peptides of β-Hb. Since advanced glycation modifications are associated with diabetic complications [22], quantification of CMV and CEV peptides may be more meaningful in the evaluation of glycemic status in diabetes. In addition, CMV and CEV show better correlation with severity of diabetes. Hence, we propose that quantification of CMV and CEV peptides of β-Hb along with HbA1c measurement, as these peptides provide better insight of glycemic status and therefore could be useful in treatment and better management of diabetes.

## Additional files

10.1186/s12014-016-9108-y Clinical characteristics of participating subjects.
10.1186/s12014-016-9108-y Fragment ion library for glycated hemoglobin peptides.
10.1186/s12014-016-9108-y The list of all modified peptides and their corresponding modified fragment ions and their MS/MS annotations.
10.1186/s12014-016-9108-y Pearson’s correlations analysis of all modified peptides with fasting blood glucose, postprandial blood glucose, HbA1c, serum triglyceride, LDL, HDL, cholesterol, VLDL, creatinine and microalbuminuria in all 35 clinical subjects.
10.1186/s12014-016-9108-y Two way ANOVA followed by Bonferonnis posttests for fold change in AUCs of β-*N*-1-Val of hemoglobin.

## Abbreviations

DF: deoxyfructosyl
CM: carboxymethyl
CE: carboxyethyl
HbA1c: glycated hemoglobin
HR–AM: high resolution–accurate mass spectrometry
PRM: parallel reaction monitoring
AGE: advanced glycation end product
AUC: area under curve
PCD: poorly controlled diabetes
PD: pre-diabetes
