# Supplementary material for: Targeted quantification of N-1-(carboxymethyl) valine and N-1-(carboxyethyl) valine peptides of β-hemoglobin for better diagnostics in diabetes
Source: Clin Proteomics. 2016 Mar 29;13:7. doi: 10.1186/s12014-016-9108-y (PMC4812615; doi:10.1186/s12014-016-9108-y)
Supplement: Supplementary file 1 — 10.1186/s12014-016-9108-y Clinical characteristics of participating subjects. [file 12014_2016_9108_MOESM1_ESM.docx]

| **Charactrestics** | **Control**  **(n=9)** | **Pre-diabetes (n=10)** | **Diabetes**  **(n=10)** | **Poorly Controlled Diabetes (n=10)** |
| --- | --- | --- | --- | --- |
| **Age (years)** | 48.10 ± 18.6 | 60.20 ± 12.4 | 54.9 ± 14.3 | 56.1 ± 13.6 |
| **Sex** | 7(M) and 2 (F) | 5(M) and 5(F) | 3 (M) and 7(F) | 9(M) and 1 (F) |
| **Fasting blood glucose (mmol/L)** | 92 ± 05.87 | 107 ± 11.39 | 136 ± 28.60 | 261 ± 108.43 |
| **Postprandial blood glucose (mmol/L)** | 104.50 ± 17.10 | 169.85 ± 51.00 | 270.18 ± 79.20 | 425.60 ± 58.90 |
| **HbA1c (%)** | 4.94 ± 0.22 | 5.96 ± 0.25 | 7.46 ± 0.619 | 9.99 ± 1.49 |
| **HbA1c (mmol/mol)** | 30 ± 0.86 | 42 ± 0.75 | 58 ± 2.00 | 86 ± 8.33 |
| **Hemoglobin (g/dl)** | 14.21 ± 1.28 | 12.86 ± 1.44 | 12.74 ± 1.66 | 14.50 ± 1.33 |
| **Serum triglyceride (mmol/L)** | 77.40 ±26.80 | 118.61 ± 49.49 | 123.09 ± 37.68 | 120.20 ± 29.10 |
| **Serum LDL (mmol/L)** | 119.20 ± 25.69 | 129.23 ± 22.21 | 131.63 ± 42.29 | 161.30 ± 25.38 |
| **Serum HDL (mmol/L)** | 48.20 ±24.18 | 41.61 ± 7.43 | 33.00 ± 5.15 | 30.90 ± 4.17 |
| **Serum Cholesterol (mmol/L)** | 170.60 ± 32.60 | 183.15 ± 30.98 | 194.36 ± 36.63 | 209.10 ± 27.66 |
| **Serum VLDL (mmol/L)** | 12.30 ± 4.3 | 22.60 ± 14.50 | 20.20 ± 5.90 | 18.97 ± 4.59 |
| **Creatinine (µmol/L)** | 0.94 ± 0.05 | 0.85 ± 0.15 | 0.84 ± 0.15 | 0.93 ± 0.11 |
| **ALT or SGPT**  **(units/liter)** | 18.40 ± 4.28 | 17.50 ± 4.29 | 17.6 ± 5.54 | 18.8 ± 4.54 |
| **AST or SGOP (units/liter)** | 18.60 ± 7.27 | 20.38 ± 4.69 | 17.2 ± 2.79 | 18.7 ± 2.49 |
| **MIC (mg/dl)** | 0.54 ± 0.05 | 0.69 ± 0.21 | 0.72 ± 0.24 | 1.63 ± 0.94 |

**Targeted quantification of N-1-(carboxymethyl) valine and N-1-(carboxyethyl) valine peptides of β-hemoglobin for better diagnostics in diabetes**

**Table S1**. Clinical characteristics of participating subjects. Continuous variables are expressed as mean ± SD and categorical variables as indicated. Fasting blood glucose; Postprandial blood glucose; HbA1c: Glycated Hemoglobin A; LDL: Low density lipoprotein; HDL: High density lipoprotein; VLDL: Very low-density lipoprotein; ALT: Alanine amino transferase; SGPT: Serum Glutamic Pyruvic Transaminase; AST: Aspartate Aminotransferase; SGOT: Serum Glutamic Oxaloacetic Transaminase; and MIC: Microalbuminuria.
