# Supplementary material for: Targeted quantification of N-1-(carboxymethyl) valine and N-1-(carboxyethyl) valine peptides of β-hemoglobin for better diagnostics in diabetes
Source: Clin Proteomics. 2016 Mar 29;13:7. doi: 10.1186/s12014-016-9108-y (PMC4812615; doi:10.1186/s12014-016-9108-y)
Supplement: Supplementary file 2 — 10.1186/s12014-016-9108-y Fragment ion library for glycated hemoglobin peptides. [file 12014_2016_9108_MOESM2_ESM.docx]

| **Sl.**  **Targeted quantification of N-1-(carboxymethyl) valine and N-1-(carboxyethyl) valine peptides of β-hemoglobin for better diagnostics in diabetes**  **Table S2.**  Fragment ion library for glycated hemoglobin peptides. Detailed information of glucose (A), glyoxyl (B) and methyglyoxal (C) derived N-valine and lysine peptides of Hb and their corresponding modified fragment ions. DFV/DFL: N-1-deoxyfructosyl-valine/lysine; CMV/CML: N-1-carboxymethyl-valine/lysine; CEV/CEL: N-1-carboxyethyl-valine/lysine; CS: Charge state; XC: Xcorr; MC: Miss cleavage; and Mod: Modification.  **No.** | **Modsite** | **Peptide start-end** | **Peptide sequence** | **Peptide MH+ Da** | **Monoisotopic m/z (mmu/ppm)** | **CS** | **XC** | **MC** | **Mod** | **Signature fragment ions** | | |
| --- | --- | --- | --- | --- | --- | --- | --- | --- | --- | --- | --- | --- |
| 1. **Glucose induced glycation modifications** | | | | | | | | | | | | |
| **Alpha chain of Hemoglobin** | | | | | | | | | | | | |
| 1 | K7 and  K 11 | 1 to 17 | VLSPAD**K***TNV**K***AAWGK | 1904.99453 | 477.00409 Da (-0.49 mmu/-1.02 ppm), | 4 | 2.05 | 1 | DFL  and CML | \| **y⁺6** \| \| --- \| \| 718.38829 \| | \| **y⁺7** \| \| --- \| \| 817.45671 \| | \| **y²⁺11** \| \| --- \| \| 719.36467 \| |
| 2 | K 7 | 1 to 11 | VLSPAD**K***TNVK | 1229.67131 | 615.33929 Da (-1.17 mmu/-1.9 ppm), | 2 | 2.53 | 1 | CML | \| **y⁺5** \| \| --- \| \| 647.37229 \| | \| **y⁺6** \| \| --- \| \| 762.39924 \| | \| **y⁺7** \| \| --- \| \| 833.43636 \| |
| 3 | K 16 | 12 to 31 | AAWG**K***VGAHAGEYGAEALER | 2205.04978 | 735.68811 Da (-2.47 mmu/-3.36 ppm), | 3 | 4.44 | 1 | DFL | \| **b⁺7** \| \| --- \| \| 832.41999 \| | \| **b⁺11** \| \| --- \| \| 1168.57461 \| | \| **y²⁺16** \| \| --- \| \| 910.44472 \| |
| 4 | K 16 | 12 to 31 | AAWG**K***VGAHAGEYGAEALER | 2101.01455 | 526.00909 Da (+1.18 mmu/+2.24 ppm), | 4 | 2.92 | 1 | CML | \| **b⁺6** \| \| --- \| \| 671.35118 \| | \| **b⁺8** \| \| --- \| \| 799.40977 \| | \| **b⁺9** \| \| --- \| \| 936.46868 \| |
| 5 | K 61 | 61 to 90 | **K***VADALTNAVAHVDDMPNALSALSDLHAHK | 3182.58649 | 637.32312 Da (-0.71 mmu/-1.11 ppm), | 5 | 3.36 | 1 | CML | \| **b⁺2** \| \| --- \| \| 286.17615 \| | \| **b⁺3** \| \| --- \| \| 357.21327 \| | \| **b⁺4** \| \| --- \| \| 472.24022 \| |
| 6 | K 90 | 62 to 92 | VADALTNAVAHVDDMPNALSALSDLHAH**K***LR | 3427.71986 | 686.34979 Da (-1.55 mmu/-2.26 ppm) | 5 | 2.58 | 1 | DFL | \| **y⁺4** \| \| --- \| \| 715.40974 \| | \| **y⁺5** \| \| --- \| \| 786.44686 \| | \| **y⁺6** \| \| --- \| \| 923.50577 \| |
| 7 | K 90 | 61 to 92 | KVADALTNAVAHVDDMPNALSALSDLHAH**K***LR | 3555.81860 | 593.47583 Da (-0.66 mmu/-1.12 ppm), | 6 | 1.55 | 2 | DFL | \| **y⁺3** \| \| --- \| \| 578.35083 \| | \| **y⁺6** \| \| --- \| \| 923.50577 \| | \| **y²⁺16** \| \| --- \| \| 953.01310 \| |
| 8 | K 139 | 100 to 141 | LLSHCLLVTLAAHLPAEFTPAVHASLDKFLASVSTVLTS**K***YR | 4635.47875 | 773.41919 Da (-3.7 mmu/-4.78 ppm), | 6 | 4.32 | 2 | CABD and CML | \| **y⁺3** \| \| --- \| \| 524.28273 \| | \| **y⁺5** \| \| \| --- \| --- \| \| 712.36244 \| \| \|  \| \| \|  \| \| | \| **y⁺6** \| \| \| --- \| --- \| \| 825.44651 \| \| \|  \| \| \|  \| \| |
| **Beta chain of Hemoglobin** | | | | | | | | | | | | |
| 9 | V 1 | 1 to 8 | **V***HLTPEEK | 1114.56072 | 557.78400 Da (-0.99 mmu/-1.77 ppm) | 2 | 1.66 | 0 | DFV | \| **b²⁺1** \| \| --- \| \| 131.56790 \| | \| **b²⁺2** \| \| --- \| \| 200.09735 \| | \| **b⁺3** \| \| --- \| \| 512.27150 \| |
| 10 | V 1 | 1 to 8 | **V***HLTPEEK | 1010.51164 | 505.75946 Da (-1.85 mmu/-3.66 ppm) | 2 | 2.01 | 0 | CMV | \| **b⁺2** \| \| --- \| \| 295.14009 \| | \| **b⁺3** \| \| --- \| \| 408.22416 \| | \| **b⁺4** \| \| --- \| \| 509.27184 \| |
| 11 | K 17 | 9 to 30 | SAVTALWG**K***VNVDEVGGEALGR | 2390.22086 | 797.41180 Da (+0.31 mmu/+0.39 ppm) | 3 | 4.56 | 1 | DFL | \| **b³⁺9** \| \| --- \| \| 359.52562 \| | \| **b³⁺11** \| \| --- \| \| 430.56273 \| | \| **b³⁺14** \| \| --- \| \| 544.94206 \| |
| 12 | K 17 | 9 to 30 | SAVTALWG**K***VNVDEVGGEALGR | 2286.17484 | 762.72980 Da (+0.75 mmu/+0.99 ppm) | 3 | 3.84 | 1 | CML | \| **y⁺15** \| \| --- \| \| 1557.78682 \| | \| **b³⁺9** \| \| --- \| \| 324.84317 \| | \| **b³⁺15** \| \| --- \| \| 543.28242 \| |
| 13 | K 144 | 133 to 146 | VVAGVANALAH**K***YH | 1507.79827 | 754.40277 Da (-1.69 mmu/-2.24 ppm), | 2 | 3.66 | 1 | CML | \| **y⁺3** \| \| --- \| \| 505.24052 \| | \| **y⁺4** \| \| --- \| \| 642.29943 \| | \| **y⁺5** \| \| --- \| \| 713.33655 \| |
| 1. **Glyoxal induced glycation modifications** | | | | | | | | | | | | |
| **Alpha Chain of Hemoglobin** | | | | | | | | | | | | |
| 1 | K 40  and  K 56 | 32 to 60 | MFLSFPTT**K***TYFPHFDLSHGSAQV**K***GHGK | 3381.6435 | 677.13452 Da (+1.46 mmu/+2.15 ppm) | 5 | 6.62 | 1 | CML and CML | \| **y⁺5** \| \| --- \| \| 584.31511 \| | \| **y⁺6** \| \| --- \| \| 683.38353 \| | \| **y⁺8** \| \| --- \| \| 882.47923 \| |
| 2 | K 40,  K 56  and  K 60 | 32 to 60 | MFLSFPTT**K***TYFPHFDLSHGSAQV**K***GHG**K*** | 3439.6412 | 688.73407 Da (-0.09 mmu/-0.14 ppm) | 5 | 5.68 | 2 | CML,  CML  and CML | \| **y⁺5** \| \| --- \| \| 642.32059 \| | \| **y⁺6** \| \| --- \| \| 741.38901 \| | \| **y⁺7** \| \| --- \| \| 869.44759 \| \|  \| |
| 3 | K 61 and  K 90 | 61 to 92 | **K***VADALTNAVAHVDDMPNALSALSDLHAH**K***LR | 3509.7777 | 878.19989 Da (-0.75 mmu/-0.85 ppm) | 4 | 5.00 | 2 | CML and CML | \| **b⁺2** \| \| --- \| \| 286.17615 \| | \| **b⁺3** \| \| --- \| \| 357.21327 \| | \| **b⁺4** \| \| --- \| \| 472.24022 \| |
|  |  |  |  |  |  |  |  |  |  | \| **y⁺3** \| \| --- \| \| 474.30348 \| | \| **y⁺4** \| \| --- \| \| 611.36239 \| | \| **y⁺5** \| \| --- \| \| 682.39951 \| |
| 4 | K 61 and  K 90 | 61 to 92 | **K***VADALTNAVAHVDD**M**PNALSALSDLHAH**K***LR | 3525.7761 | 705.96106 Da (+0.11 mmu/+0.16 ppm) | 5 | 2.28 | 2 | CML,  OXDN  and CML | \| **b⁺2** \| \| --- \| \| 286.17615 \| | \| **b⁺3** \| \| --- \| \| 357.21327 \| | \| **b⁺4** \| \| --- \| \| 472.24022 \| |
|  |  |  |  |  |  |  |  |  |  | \| **y⁺3** \| \| --- \| \| 474.30348 \| | \| **y⁺4** \| \| --- \| \| 611.36239 \| | \| **y⁺5** \| \| --- \| \| 682.39951 \| |
| 5 | K 90 | 62 to 92 | VADALTNAVAHVDDMPNALSALSDLHAH**K***LR | 3323.6833 | 665.54248 Da (+0.61 mmu/+0.91 ppm) | 5 | 6.53 | 1 | CML | **y⁺3**  474.30348 | **y⁺4**  611.36239 | **y⁺5**  682.39951 |
| 6 | K 90 | 61 to 92 | KVADALTNAVAHVDDMPNALSALSDLHAH**K***LR | 3451.7783 | 691.16150 Da (+0.63 mmu/+0.91 ppm) | 5 | 5.51 | 2 | CML | \| **y⁺3** \| \| --- \| \| 474.30348 \| | \| **y⁺4** \| \| --- \| \| 611.36239 \| | \| **y⁺5** \| \| --- \| \| 682.39951 \| |
| 7 | K 90 | 62 to 92 | VADALTNAVAHVDD**M**PNALSALSDLHAH**K***LR | 3339.6729 | 668.74042 Da (-0.44 mmu/-0.66 ppm) | 5 | 3.35 | 1 | OXDN  and  CML | **y⁺3**  474.30348 | **y⁺4**  611.36239 | **y⁺5**  682.39951 |
| 8 | K 139 | 128 to 141 | FLASVSTVLTS**K***YR | 1629.8869 | 543.96716 Da (+0.75 mmu/+1.37 ppm) | 3 | 1.11 | 1 | CML | \| **y⁺3** \| \| --- \| \| 524.28273 \| | \| **y⁺4** \| \| --- \| \| 611.31476 \| | \| **y⁺5** \| \| --- \| \| 712.36244 \| \|  \| \|  \| |
| 9 | K 139 | 100 to 141 | LLSH**C**LLVTLAAHLPAEFTPAVHASLDKFLASVSTVLTS**K***YR | 4635.4970 | 773.42224 Da (-0.64 mmu/-0.83 ppm) | 6 | 2.38 | 2 | CABD  and  CML | \| **y⁺3** \| \| --- \| \| 524.28273 \| | \| **y⁺4** \| \| --- \| \| 611.31476 \| | \| **y⁺5** \| \| --- \| \| 712.36244 \| |
| **Beta Chain of Hemoglobin** | | | | | | | | | | | | |
| 10 | V 1 | 1 to 8 | **V***HLTPEEK | 1010.5151 | 505.76123 Da (-0.08 mmu/-0.15 ppm) | 2 | 1.68 | 0 | CMV | **b^+^1**  158.081 | **b^+^2**  295.140 | **b^+^3**  408.224 |
| 11 | V 1  and  K 8 | 1 to 17 | **V***HLTPEE**K***SAVTALWGK | 1982.0245 | 661.34637 Da (+0.52 mmu/+0.78 ppm) | 3 | 2.75 | 1 | CMV  and  CML | \| **b⁺2** \| \| --- \| \| 295.14009 \| | \| **b⁺3** \| \| --- \| \| 408.22416 \| | \| **b⁺4** \| \| --- \| \| 509.27184 \| |
| 12 | V 1,  K 8 and  K 17 | 1 to 30 | **V***HLTPEE**K***SAVTALWG**K***VNVDEVGGEALGR | 3335.6835 | 1112.56604 Da (+2.67 mmu/+2.4 ppm), | 3 | 6.31 | 2 | CMV,  CML  and  CML | \| **b⁺2** \| \| --- \| \| 295.14009 \| | \| **b⁺8** \| \| --- \| \| 1050.51025 \| | \| **y⁺14** \| \| --- \| \| 1500.76535 \| |
| 13 | V 1,  K 8 and  K 17 | 1 to 30 | **V***HLTPEE**K***SAVTALWG**K***VNVDEVGGEALGR | 3349.6951 | 1117.23657 Da (+1.32 mmu/+1.18 ppm) | 3 | 5.53 | 2 | CEV,  CML  and  CML | \| **b⁺2** \| \| --- \| \| 309.15574 \| | \| **b⁺10** \| \| --- \| \| 1222.59505 \| | \| **y⁺14** \| \| --- \| \| 1500.76535 \| |
| 14 | K 59 | 41 to 61 | **FFESFGDLSTPDAVMGNPK*VK** | 2360.1113 | 787.37531 Da (-0.08 mmu/-0.11 ppm) | 3 | 2.46 | 1 | OXDN  and CML | \| **y⁺7** \| \| --- \| \| 847.43427 \| | \| **y⁺8** \| \| --- \| \| 946.50269 \| | \| **y⁺9** \| \| --- \| \| 1017.53981 \| |
| 15 | K 59  and  K 61 | 41 to 65 | FFESFGDLSTPDAV**M**GNP**K***V**K***AHGK | 2811.3238 | 703.58643 Da (-1.42 mmu/-2.02 ppm) | 4 | 2.37 | 2 | OXDN,  CML  and  CML | \| **y⁺8** \| \| --- \| \| 980.55240 \| | \| **y⁺10** \| \| --- \| \| 1151.61680 \| | \| **y⁺11** \| \| --- \| \| 1298.65221 \| |
| 16 | K 59  and  K 61 | 41 to 61 | FFESFGDLSTPDAV**M**GNP**K***V**K*** | 2418.1188 | 806.71112 Da (+0.57 mmu/+0.71 ppm), | 3 | 2.50 | 1 | OXDN,  CML  and  CML | \| **y⁺3** \| \| --- \| \| 490.28716 \| | \| **y⁺4** \| \| --- \| \| 587.33993 \| | \| **y⁺7** \| \| --- \| \| 905.43974 \| |
| 17 | K 65 and  K 66 | 62 to 82 | AHG**K*K***VLGAFSDGLAHLDNLK | 2307.2118 | 577.55841 Da (+0.63 mmu/+1.1 ppm) | 4 | 2.32 | 2 | CML  and CML | \| **b⁺5** \| \| --- \| \| 638.32567 \| | \| **b⁺6** \| \| --- \| \| 737.39409 \| | \| **b⁺9** \| \| --- \| \| 978.53675 \| |
| 18 | K 66 and  K 82 | 66 to 95 | **K***VLGAFSDGLAHLDNL**K***GTFATLSELH**C**DK | 3373.6704 | 675.53992 Da (-0.59 mmu/-0.88 ppm) | 5 | 2.19 | 2 | CML,  CML and  CABD | \| **b⁺4** \| \| --- \| \| 456.28169 \| | \| **b⁺5** \| \| --- \| \| 527.31881 \| | \| **b⁺6** \| \| --- \| \| 674.38723 \| |
| 19 | K 120 | 105 to 132 | LLGNVLVCVLAHHFG**K***EFTPPVQAAYQK | 3194.6810 | 1065.56519 Da (-0.35 mmu/-0.33 ppm), | 3 | 5.71 | 1 | CABD  And  CML | \| **y⁺14** \| \| --- \| \| 1621.82211 \| | \| **y⁺15** \| \| --- \| \| 1768.89053 \| | \| **y⁺16** \| \| --- \| \| 1905.94944 \| |
| 20 | K 144 | 133 to 146 | **VVAGVANALAHK*YH** | 1507.8036 | 503.27274 Da (+0.67 mmu/+1.34 ppm) | 3 | 3.23 | 1 | CML | \| **y⁺3** \| \| --- \| \| 505.24052 \| | \| **y⁺4** \| \| --- \| \| 642.29943 \| | \| **y⁺5** \| \| --- \| \| 713.33655 \| |
| 1. **Methyl glyoxal induced glycation modifications** | | | | | | | | | | | | |
| **Alpha Chain of Hemoglobin** | | | | | | | | | | | | |
| 1 | K 61 | 61 to 90 | **K***VADALTNAVAHVDDMPNALSALSDLHAHK | 3196.5965 | 640.12512 Da (-1.84 mmu/-2.87 ppm) | 5 | 2.65 | 1 | CEL | \| **b⁺2** \| \| --- \| \| 300.19180 \| | \| **b⁺3** \| \| --- \| \| 486.25587 \| | \| **b⁺4** \| \| --- \| \| 557.29299 \| |
| 2 | K 11 | 8 to 16 | TNV**K***AAWGK | 1046.5608 | 523.78406 Da (-1.07 mmu/-2.04 ppm) | 2 | 1.91 | 1 | CEL | \| **b⁺4** \| \| --- \| \| 515.28241 \| | \| **b⁺5** \| \| --- \| \| 586.31953 \| | – |
| 3 | K 90 | 62 to 92 | VADALTNAVAHVDDMPNALSALSDLHAH**K***LR | 3337.6939 | 668.34460 Da (-0.4 mmu/-0.6 ppm) | 5 | 4.94 | 1 | CEL | \| **y⁺3** \| \| --- \| \| 488.31913 \| | \| **y⁺4** \| \| --- \| \| 625.37804 \| | \| **y⁺5** \| \| --- \| \| 696.41516 \| |
| 4 | K 90 | 61 to 92 | KVADALTNAVAHVDDMPNALSALSDLHAH**K***LR | 3465.7984 | 693.96552 Da (+1.52 mmu/+2.19 ppm) | 4 | 1.52 | 2 | CEL | \| **y⁺3** \| \| --- \| \| 488.31913 \| | \| **y⁺4** \| \| --- \| \| 625.37804 \| | \| **y⁺5** \| \| --- \| \| 696.41516 \| |
| 5 | K 139 | 128 to 141 | FLASVSTVLTS**K***YR | 1643.9034 | 548.63934 Da (+1.04 mmu/+1.9 ppm), | 3 | 1.51 | 1 | CEL | \| **y⁺4** \| \| --- \| \| 625.33041 \| | \| **y⁺5** \| \| --- \| \| 726.37809 \| | \| **y⁺6** \| \| --- \| \| 839.46216 \| |
| **Beta chain of Hemoglobin** | | | | | | | | | | | | |
| 6 | V 1 | 1 to 8 | **V***HLTPEEK | 1024.5297 | 512.76849 Da (-0.64 mmu/-1.25 ppm) | 2 | 1.14 | 0 | CEV | **b^+^1**  172.096 | **b^+^2**  309.155 | – |
| 7 | V 1  and  K 8 | 1 to 17 | **V***HLTPEE**K***SAVTALWGK | 1996.0387 | 499.76514 Da (+0.01 mmu/+0.02 ppm) | 3 | 2.41 | 1 | CEV  And  CML | \| **b⁺2** \| \| --- \| \| 309.15574 \| | \| **b⁺3** \| \| --- \| \| 422.23981 \| | \| **b⁺4** \| \| --- \| \| 523.28749 \| |
| 8 | K 59 | 41 to 61 | FFESFGDLSTPDAV**M**GNP**K***VK | 2374.1282 | 792.04761 Da (+0.33 mmu/+0.42 ppm) | 3 | 1.10 | 1 | OXDN  and  CEL | \| **y⁺4** \| \| --- \| \| 543.35010 \| | \| **y⁺6** \| \| --- \| \| 714.41450 \| | \| **y⁺7** \| \| --- \| \| 861.44992 \| |
| 9 | K 144 | 133 to 146 | VVAGVANALAH**K***YH | 1521.8168 | 507.94379 Da (-0.16 mmu/-0.32 ppm) | 3 | 2.58 | 1 | CEL | \| **y⁺3** \| \| --- \| \| 519.25617 \| | \| **y⁺4** \| \| --- \| \| 656.31508 \| | \| **y⁺5** \| \| --- \| \| 727.35220 \| |

Static modification: Carbamidomethyl (57.02146 Da), CS-Charge state, XC- Xcorr, MC-Missed cleavage
