# Supplementary material for: Targeted quantification of N-1-(carboxymethyl) valine and N-1-(carboxyethyl) valine peptides of β-hemoglobin for better diagnostics in diabetes
Source: Clin Proteomics. 2016 Mar 29;13:7. doi: 10.1186/s12014-016-9108-y (PMC4812615; doi:10.1186/s12014-016-9108-y)
Supplement: Supplementary file 3 — 10.1186/s12014-016-9108-y The list of all modified peptides and their corresponding modified fragment ions and their MS/MS annotations. [file 12014_2016_9108_MOESM3_ESM.docx]

**Additional File 3**

Targeted quantification of glycated peptides of hemoglobin by mass spectrometry: Carboxymethylation, not deoxyfructosylation, is the predominant modification of N-1β-valine

**Legends**

A. MS/MS spectrum for mentioned peptide *in vitro*

B. Representative spectrum of co-eluting fragment ions *in vivo*

C. Bar graph depicting average cumulative AUC of co-eluted fragments in control samples

D. Relative intensities of co-eluted fragments in individual control samples in technical replicates

E. Bar graph depicting average cumulative AUC of co-eluted fragments in prediabetes samples

F. Relative intensities of co-eluted fragments in individual prediabetes samples in technical replicates

G. Bar graph depicting average cumulative AUC of co-eluted fragments in diabetes samples

H. Relative intensities of co-eluted fragments in individual diabetes samples in technical replicates

I. Bar graph depicting average cumulative AUC of co-eluted fragments in poorly controlled diabetes samples

J. Relative intensities of co-eluted fragments in individual poorly controlled diabetes samples in technical replicates

**Modified peptides of α-hemoglobin**

1. **VLSPADK*TNVK:** K7-CML (58.00548 Da), Charge: +2, Monoisotopic m/z: 615.33929 Da (-1.17 mmu/-1.9 ppm), MH+: 1229.67131 Da.

**B.**

**A.**

**
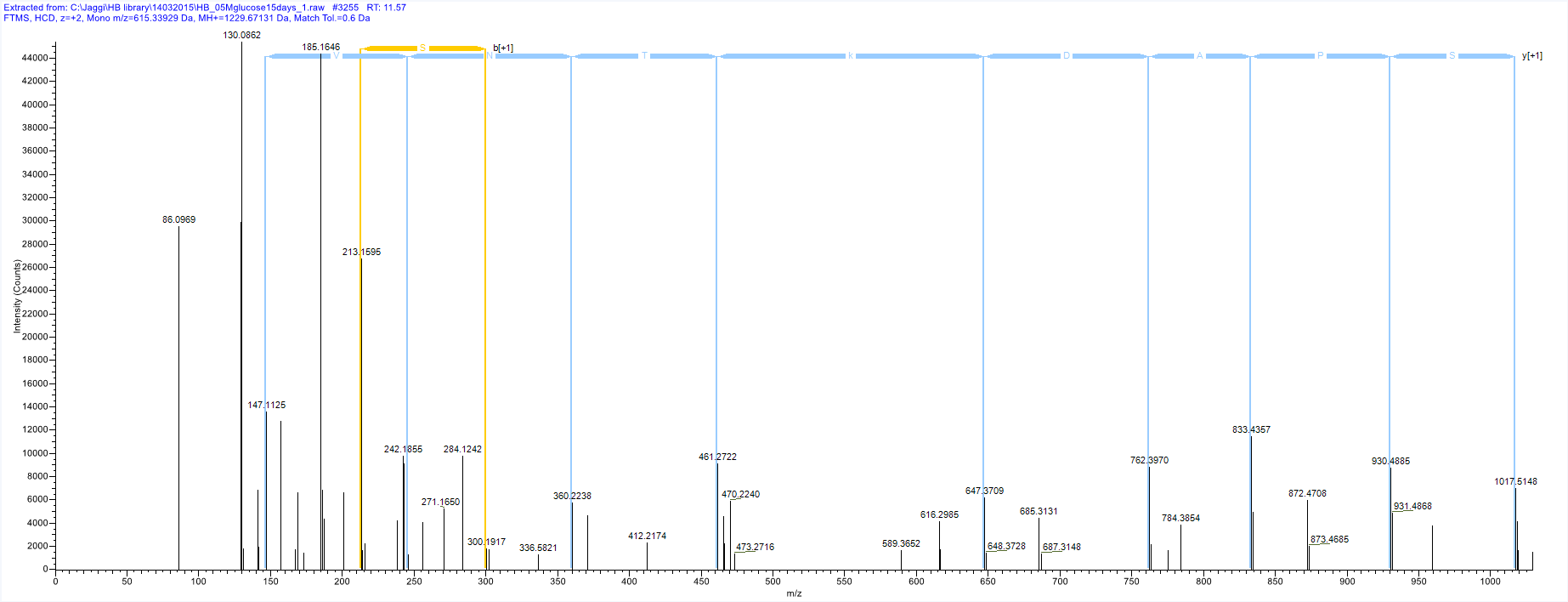

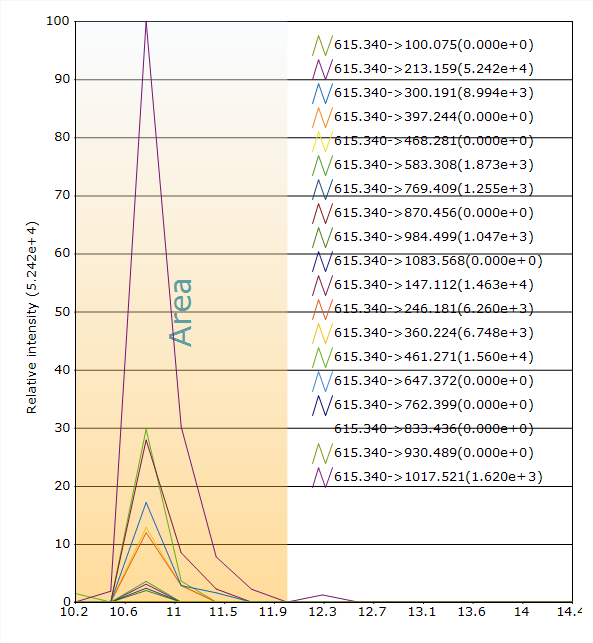
**

**
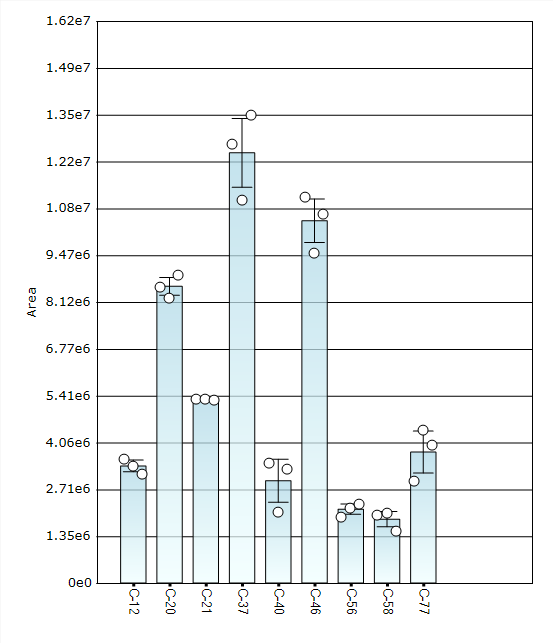

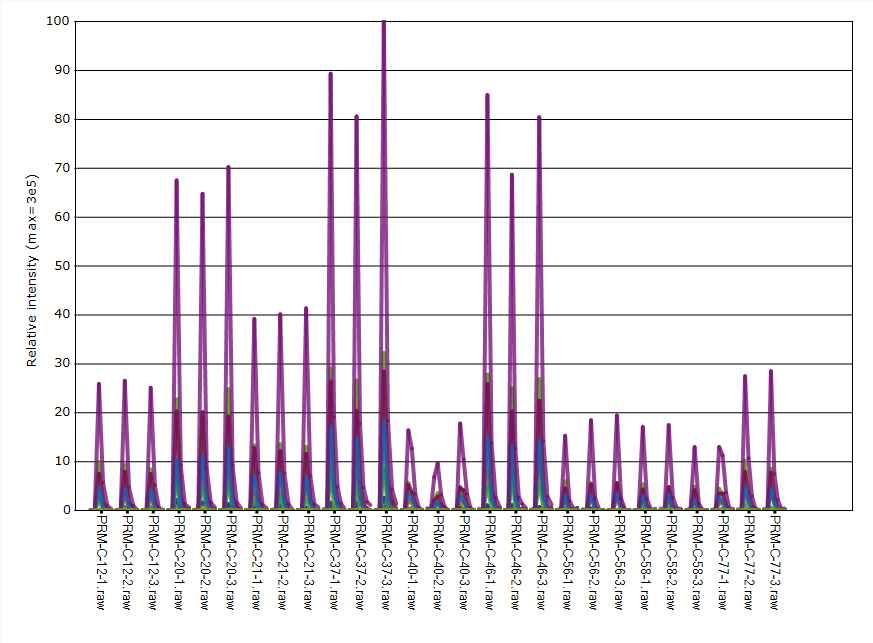
**

**D**

**C.**

**
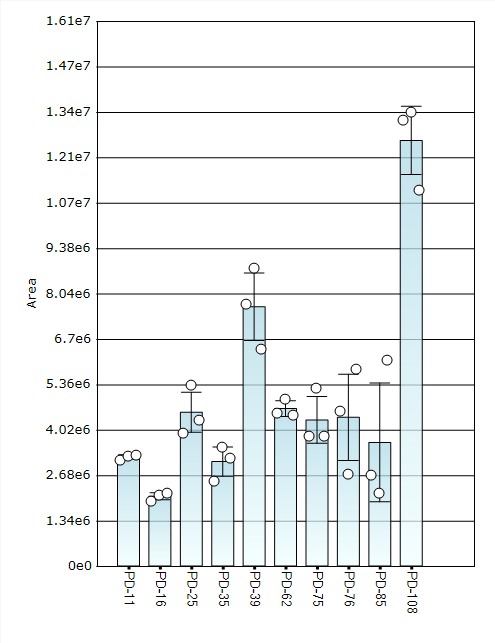

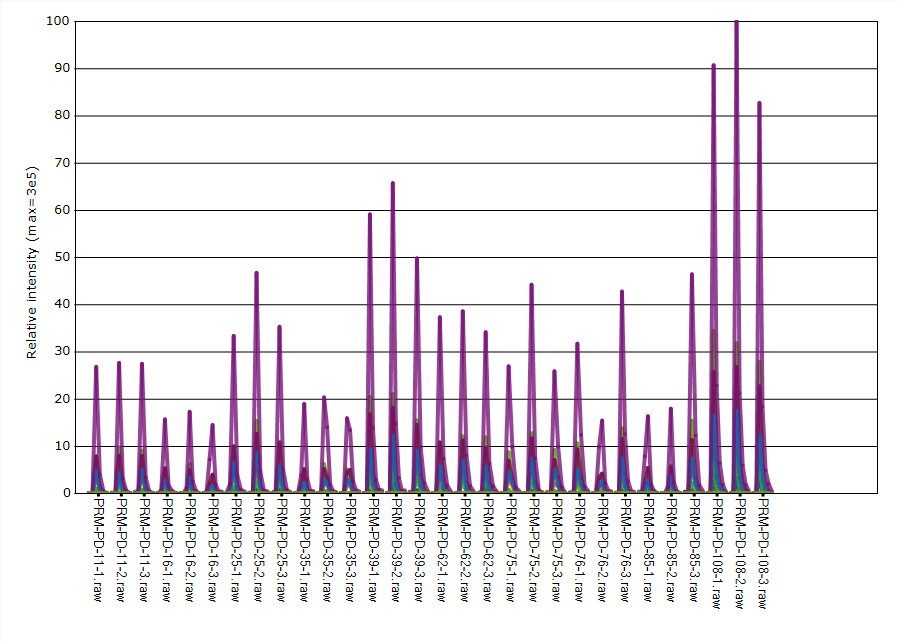
**

**F**

**E**

**
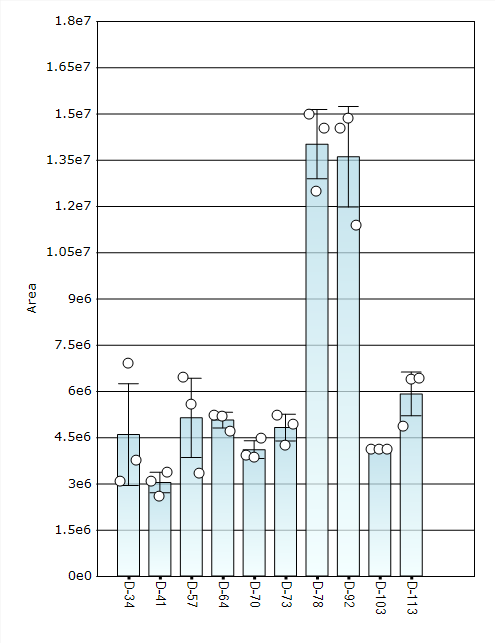

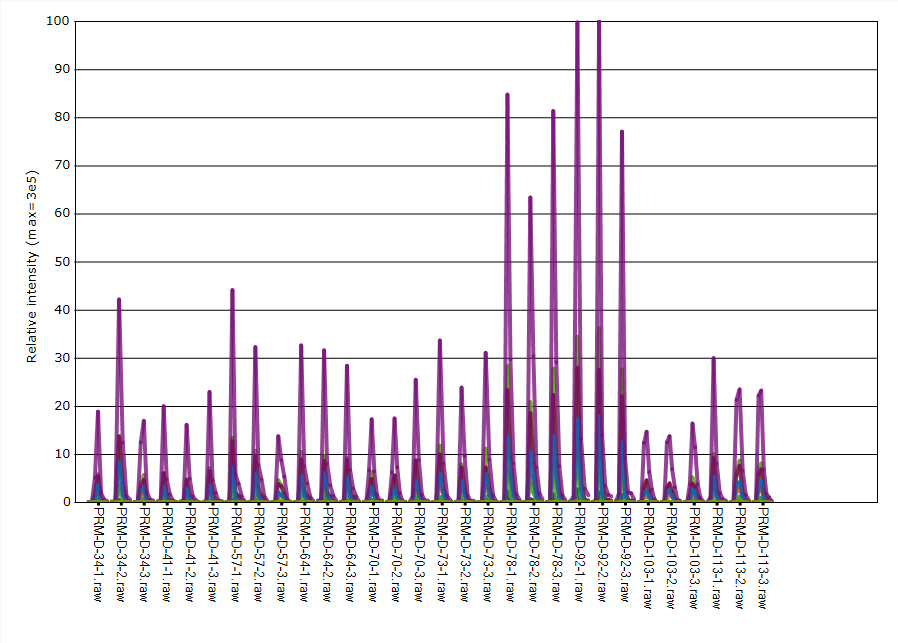
**

**H**

**G**

**
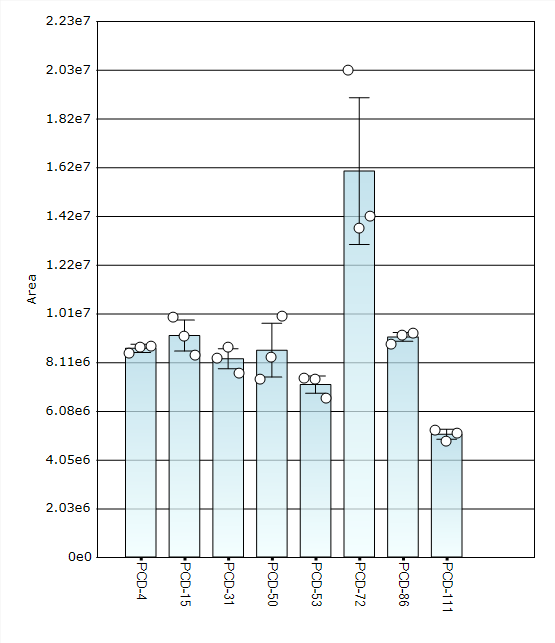

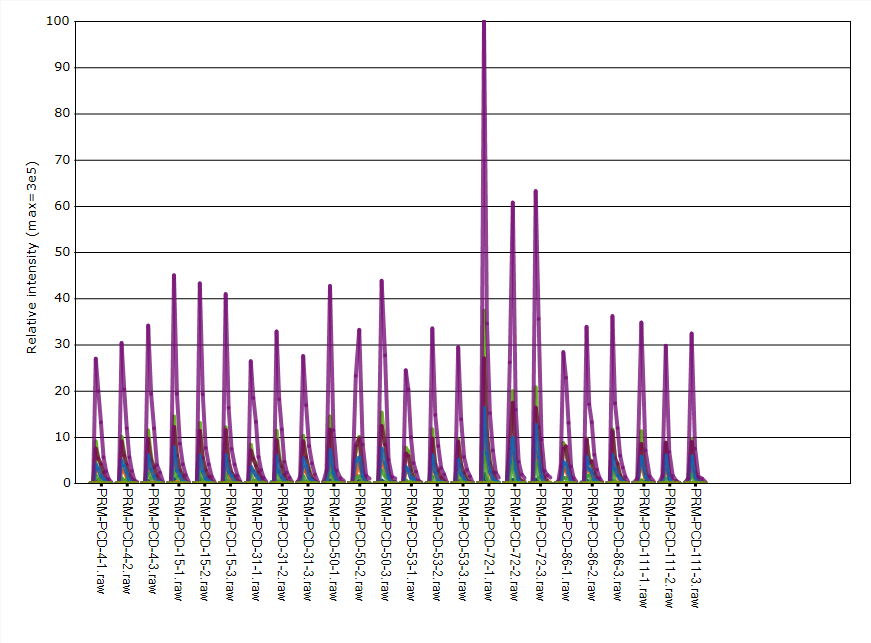
**

**J**

**I**

1. **AAWGK-(DFL)VGAHAGEYGAEALER**: K5-DFL (162.05282 Da),Charge: +3, Monoisotopic m/z: 735.68811 Da (-2.47 mmu/-3.36 ppm), MH+: 2205.04978 Da.

**
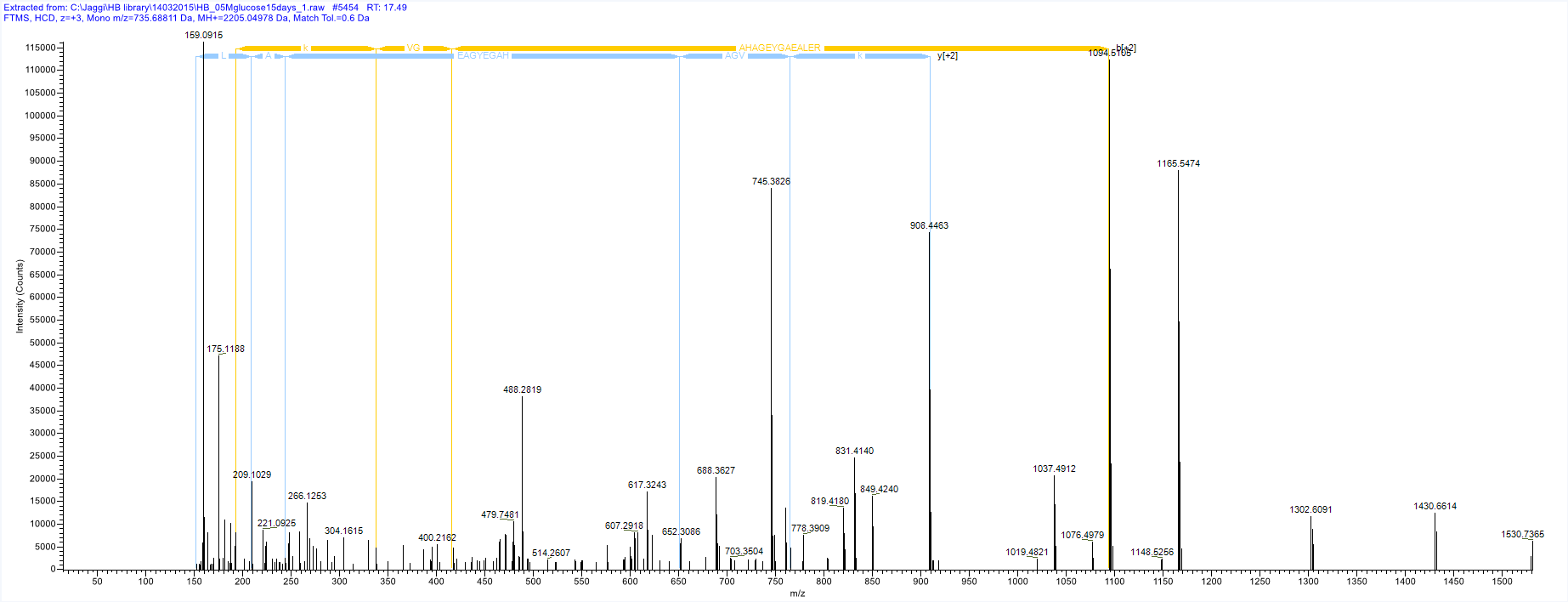

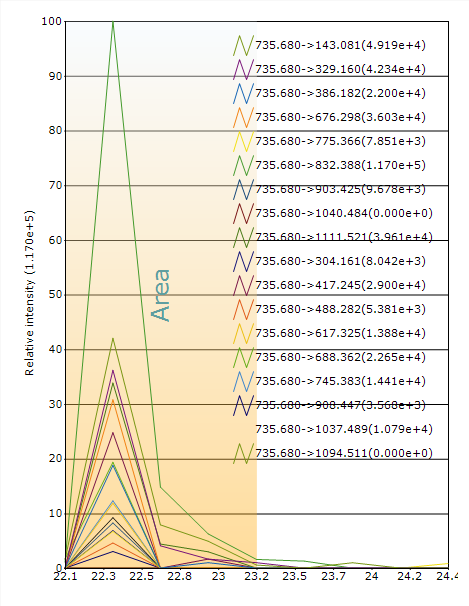
**

**B**

**A**

**
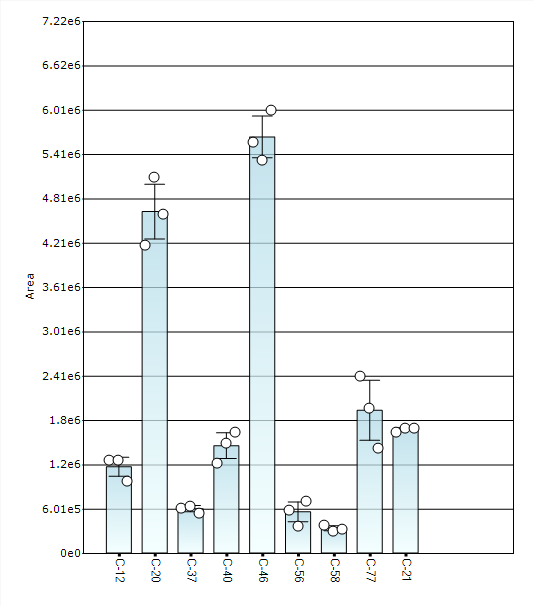

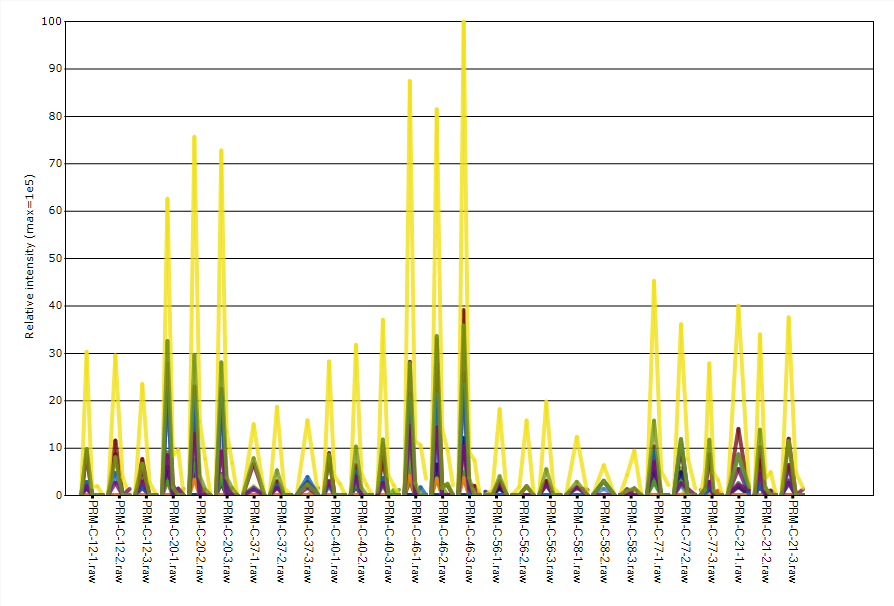
**

**D**

**C**

**E**

**
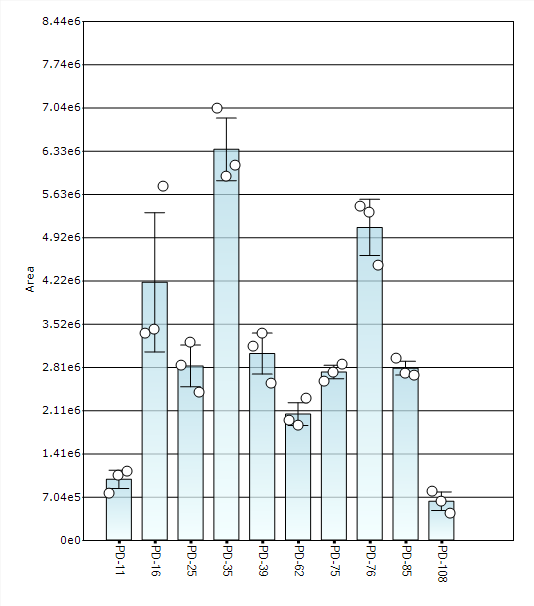

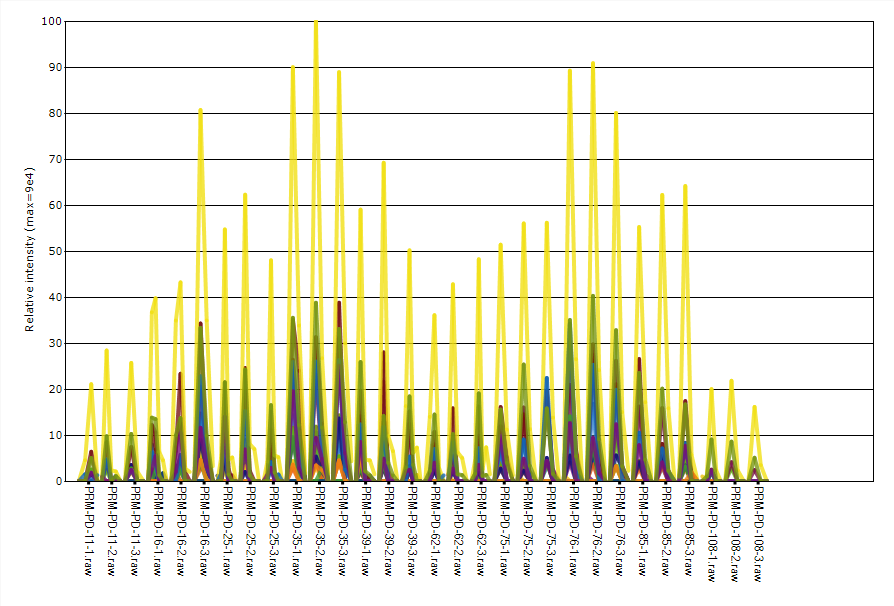
**

**F**

**
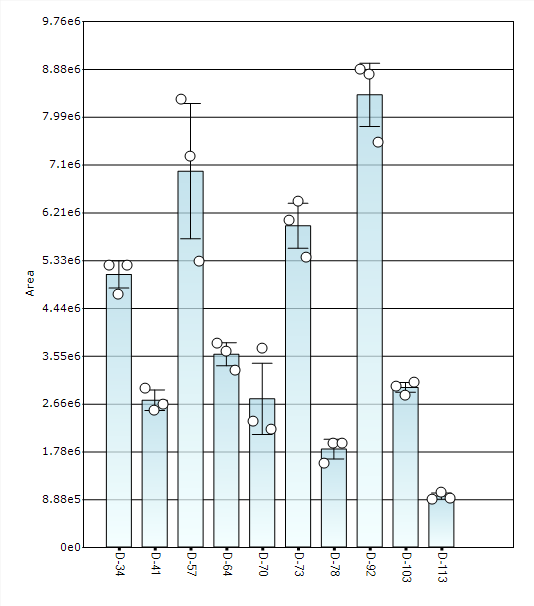

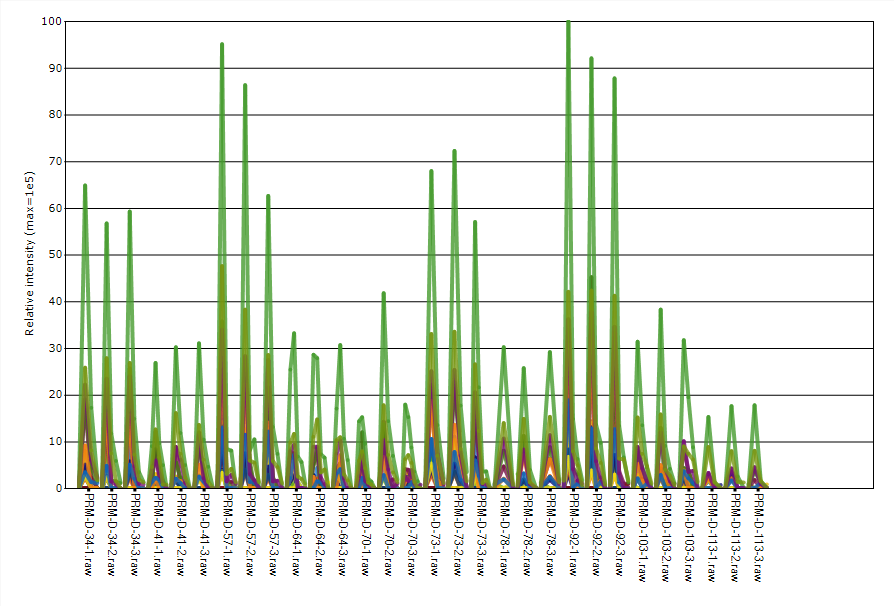
**

**H**

**G**

**
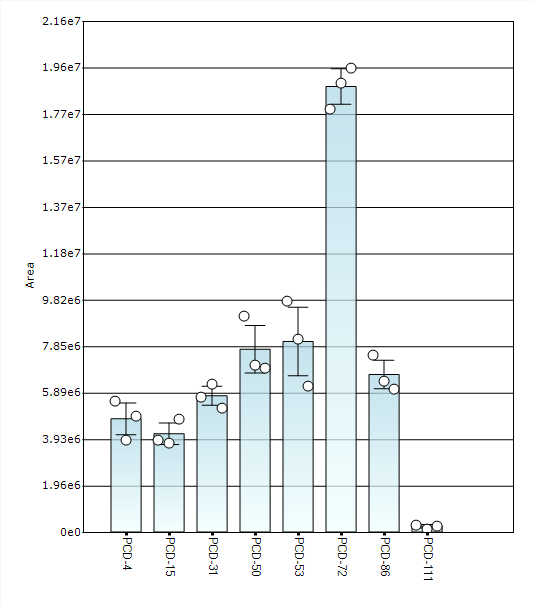

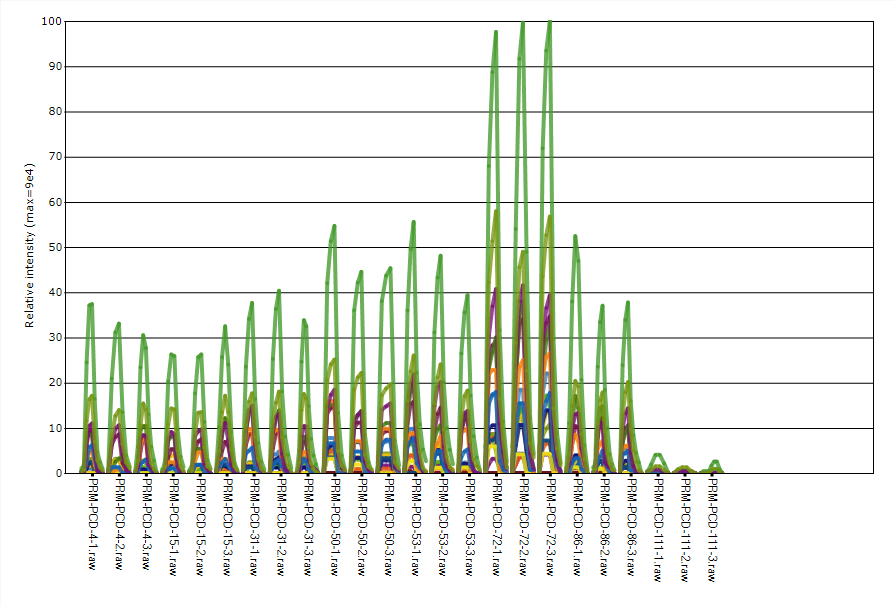
**

**J**

**I**

1. **AAWGK-(CML)VGAHAGEYGAEALER:** K5-CML (58.00548 Da), Charge: +4, Monoisotopic m/z: 526.00909 Da (+1.18 mmu/+2.24 ppm), MH+: 2101.01455 Da.

**
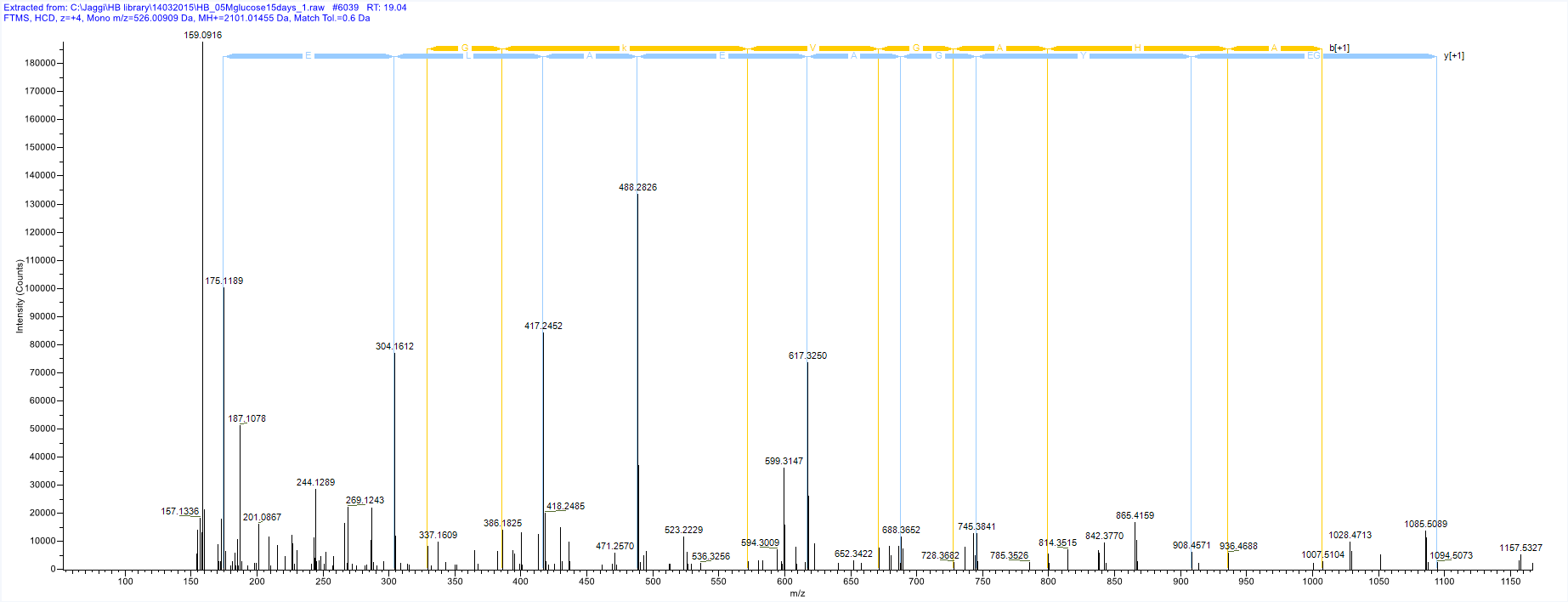

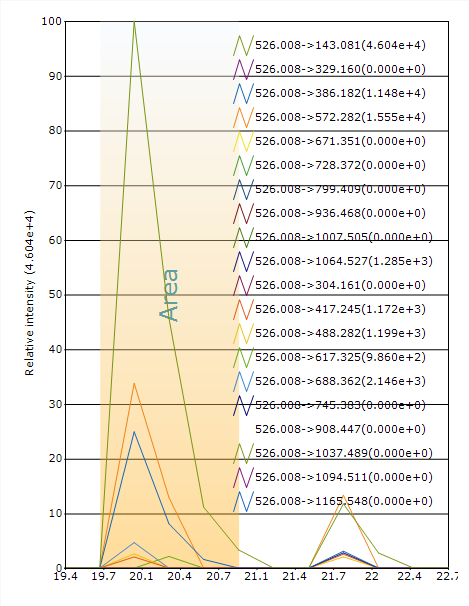
**

**B**

**A**

**
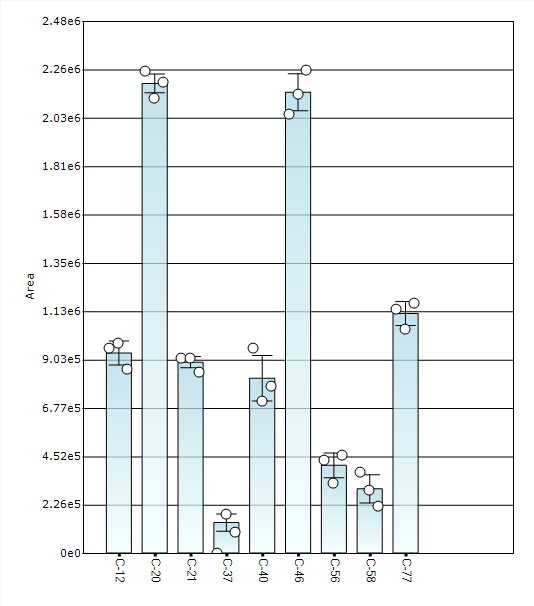

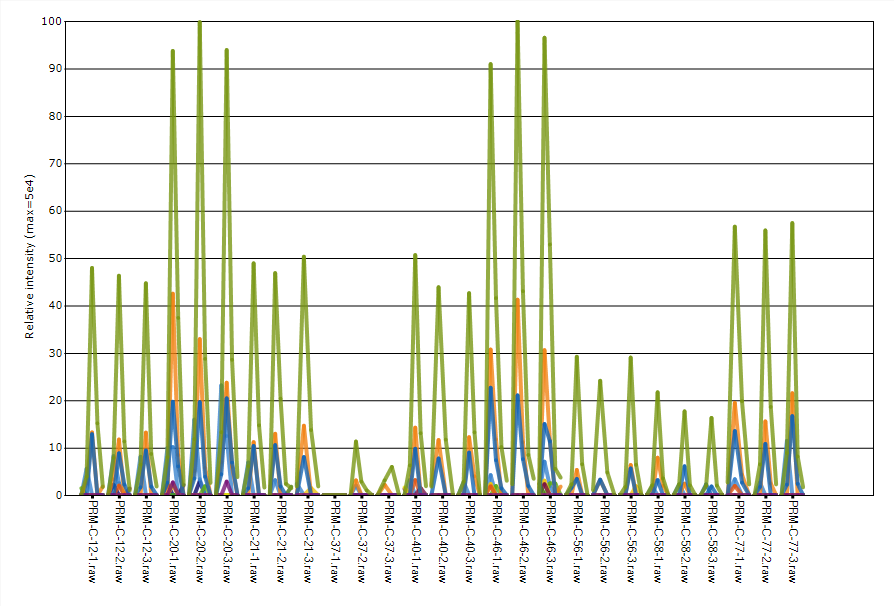
**

**C**

**D**

**
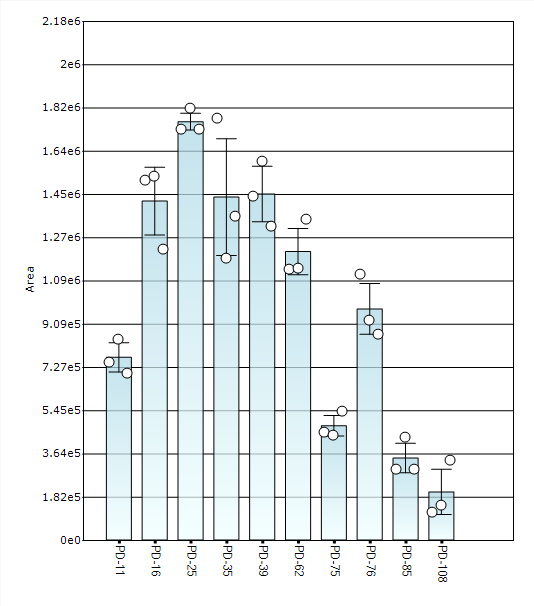

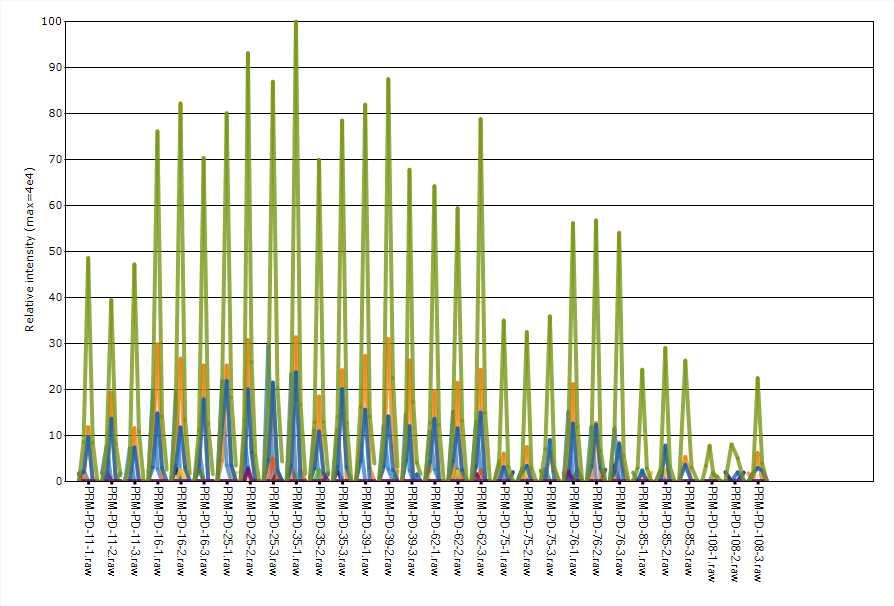
**

**E**

**F**

**
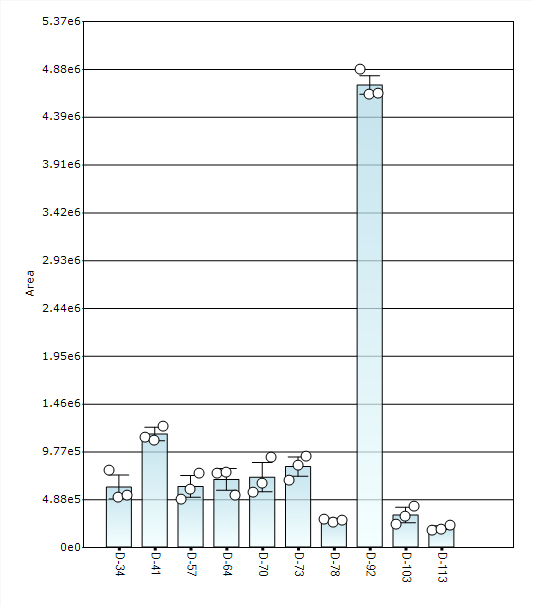

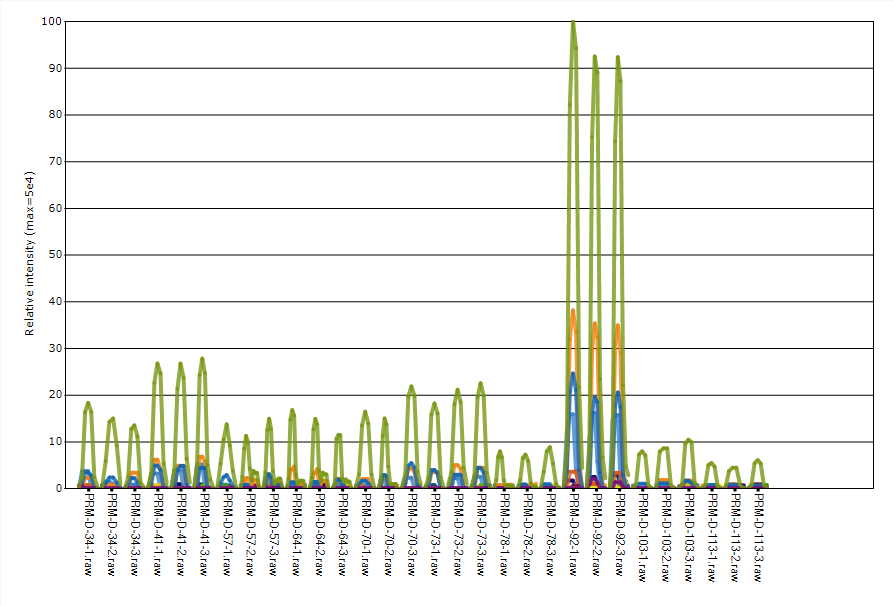
**

**H**

**G**

**
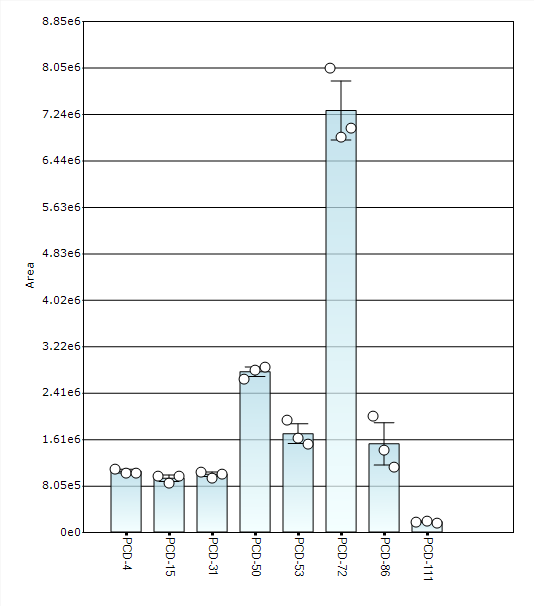

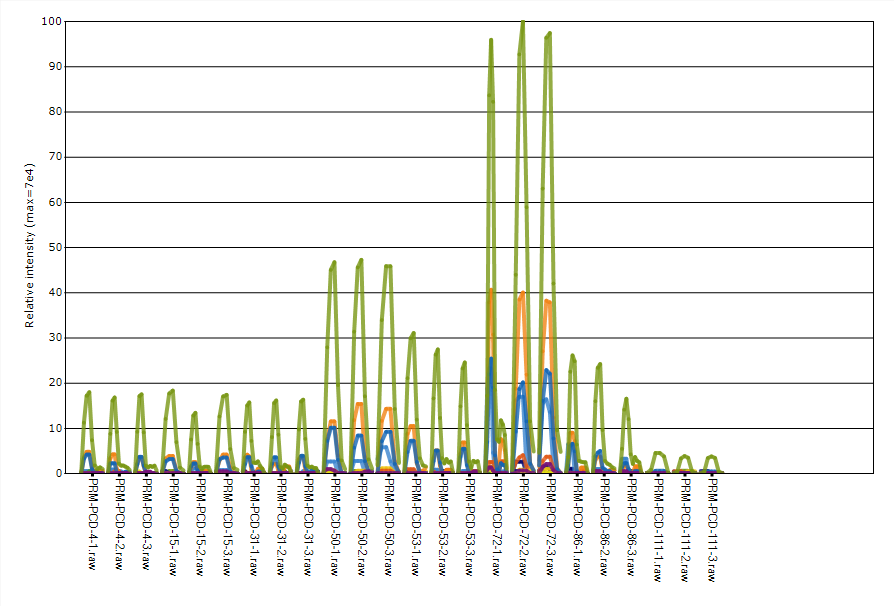
**

**J**

**I**

1. **K- (CML)VADALTNAVAHVDDMPNALSALSDLHAHK:** K1-CML (58.00548 Da), Charge: +5, Monoisotopic m/z: 637.32312 Da (-0.71 mmu/-1.11 ppm), MH+: 3182.58649 Da.

**
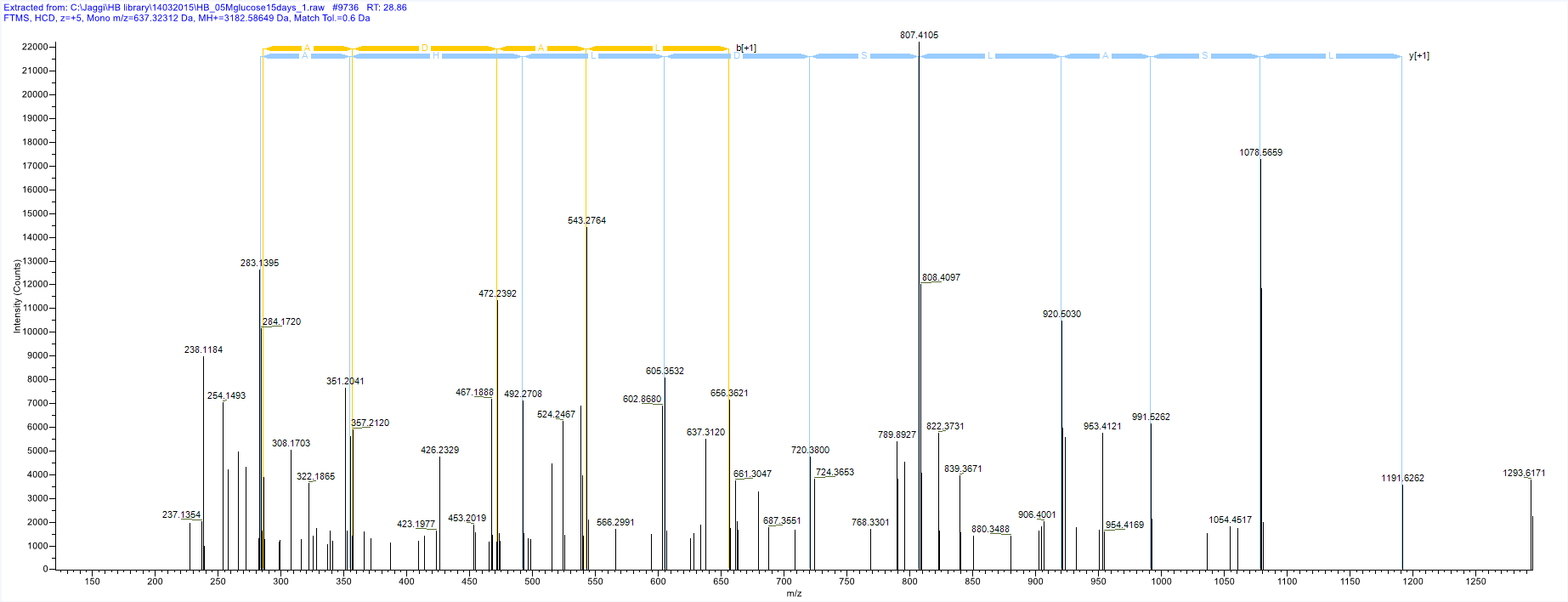

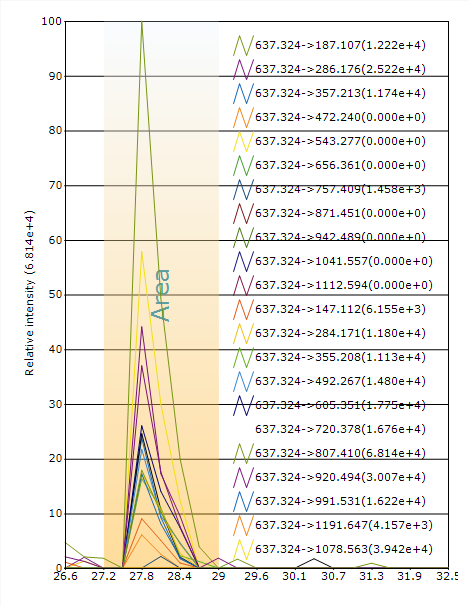
**

**B**

**A**

**
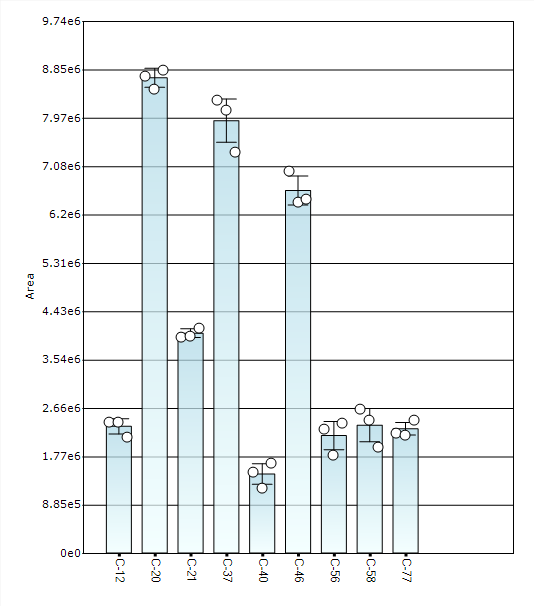

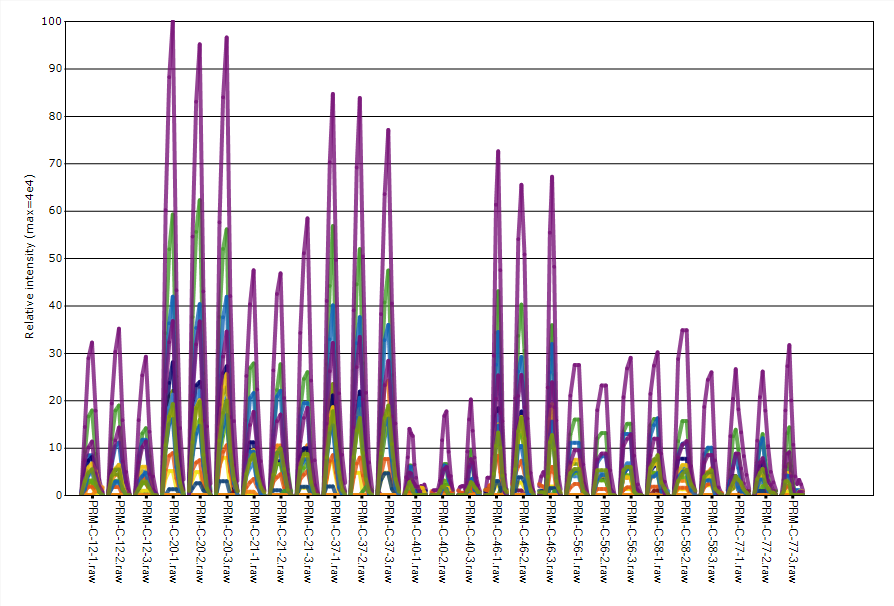
**

**D**

**C**

**
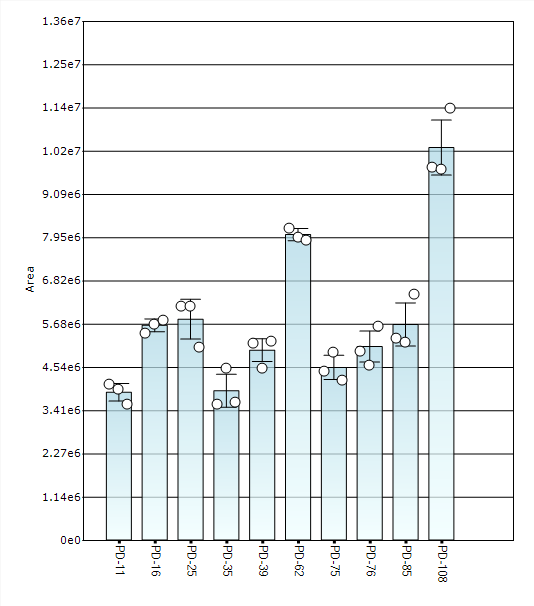

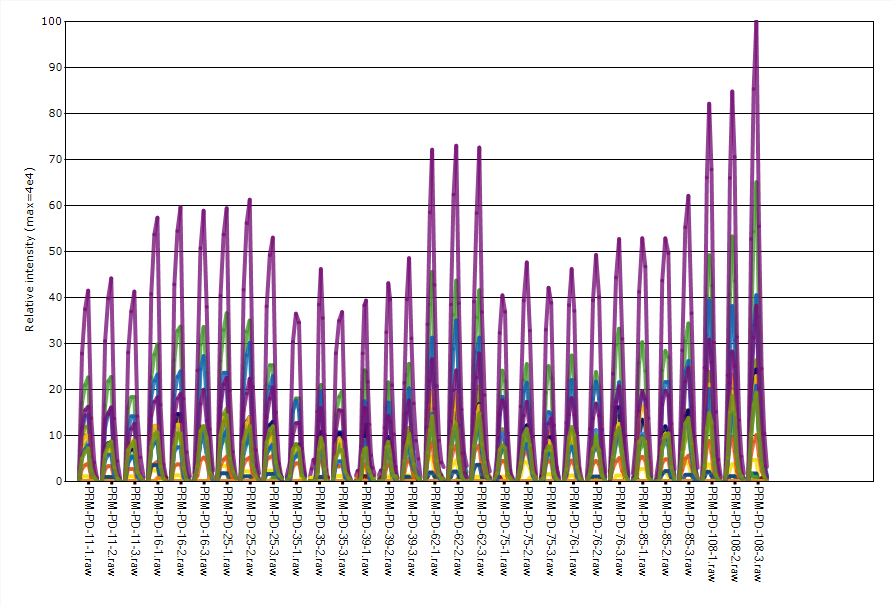
**

**F**

**E**

**
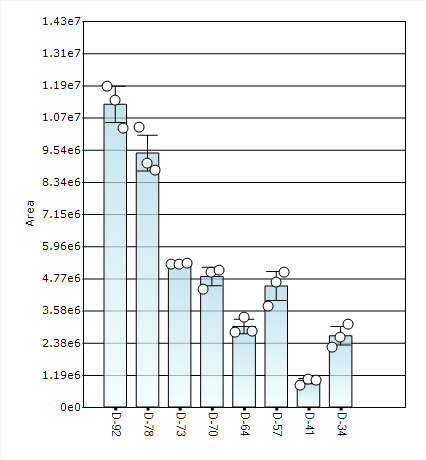

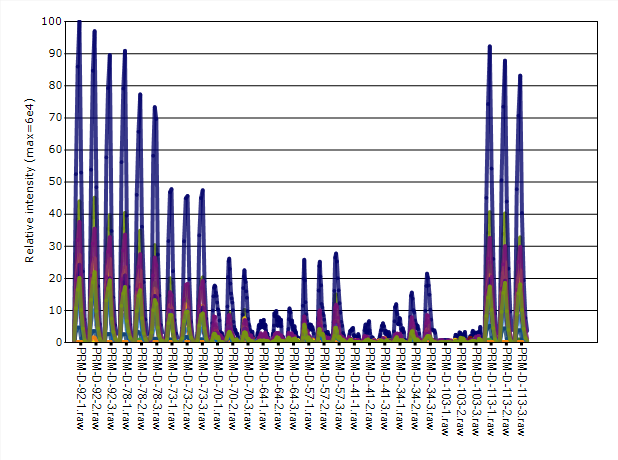
**

**H**

**G**

**
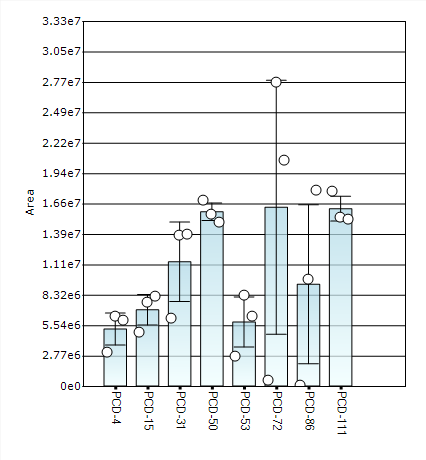

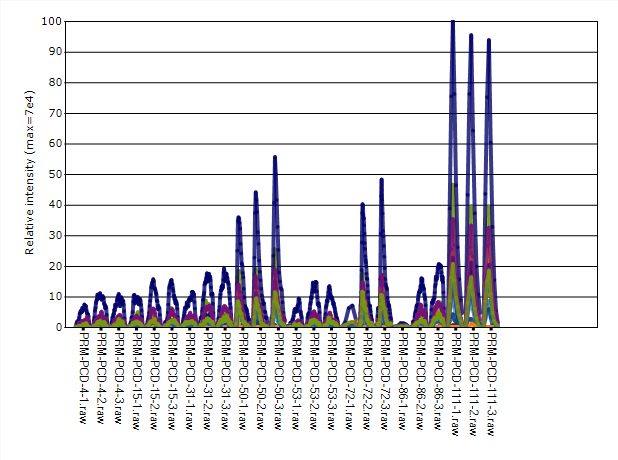
**

**J**

**I**

1. **VADALTNAVAHVDDMPNALSALSDLHAHK-(DFL)LR:** K29-DFL (162.05282 Da), Charge: +5, Monoisotopic m/z: 686.34979 Da (-1.55 mmu/-2.26 ppm), MH+: 3427.71986 Da.

**B**

**
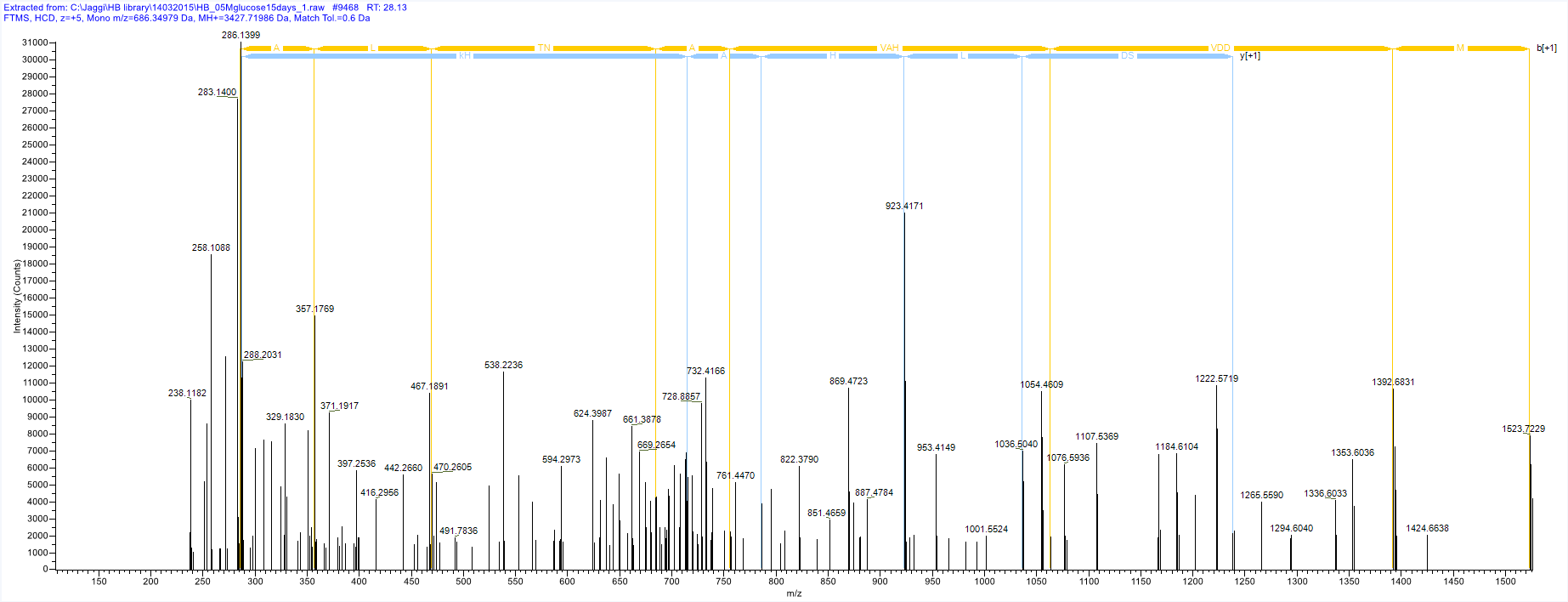

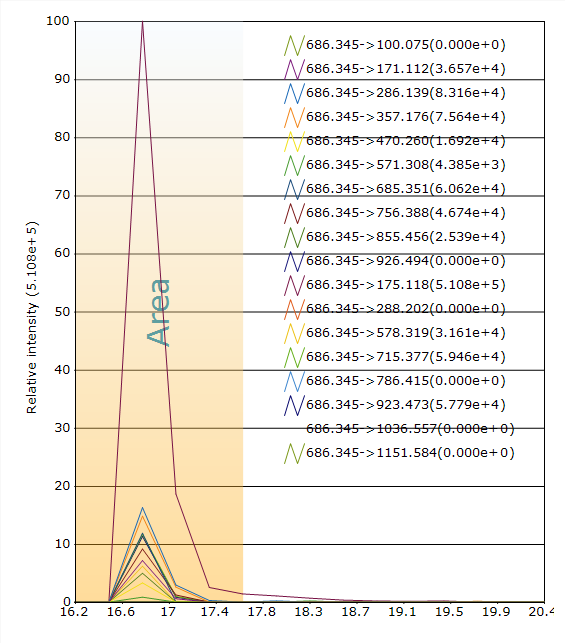
**

**A**

**
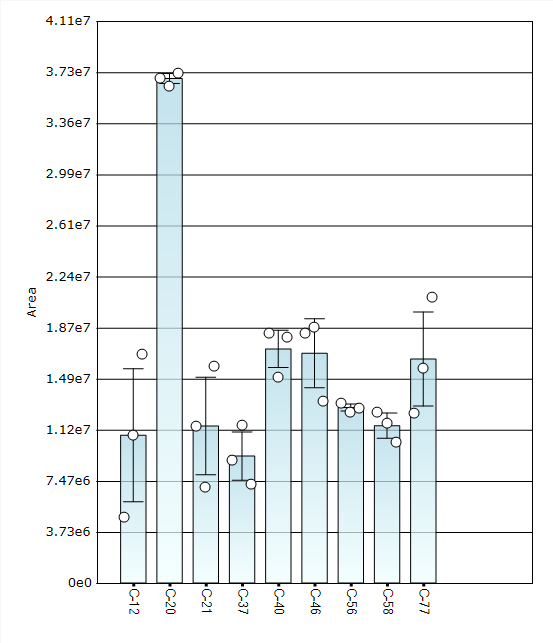

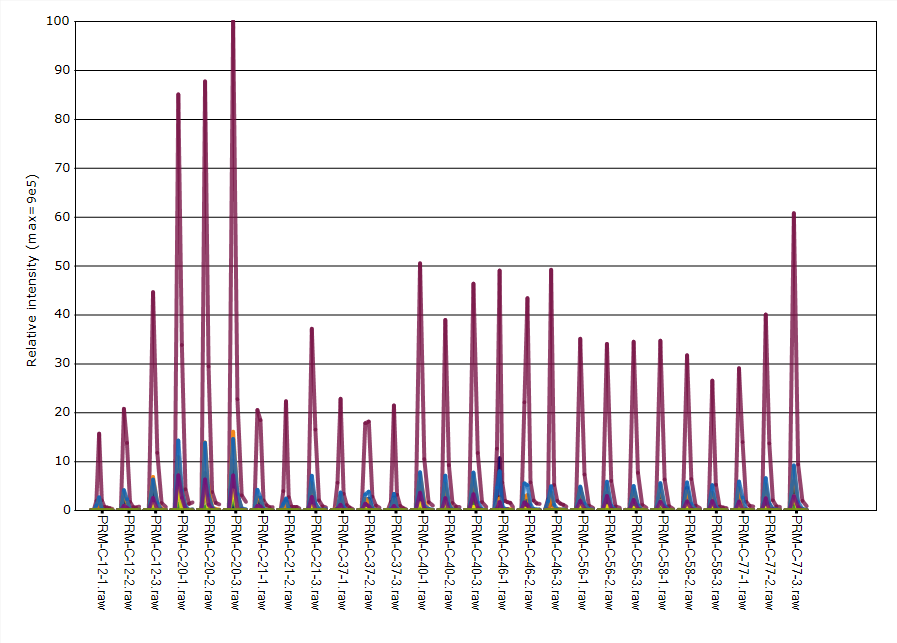
**

**D**

**C**

**
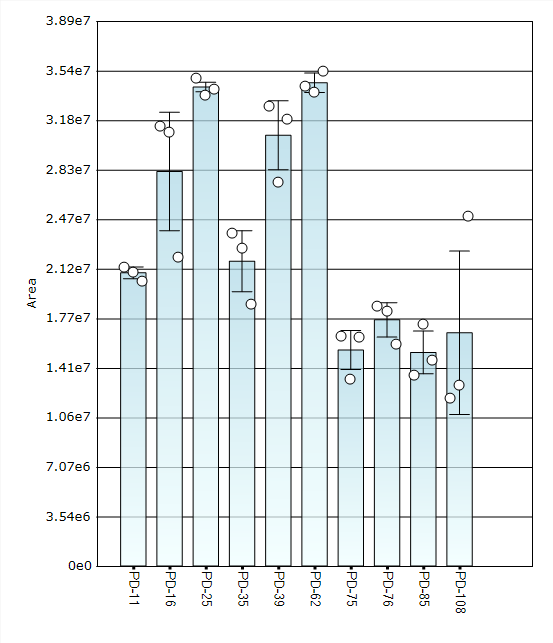

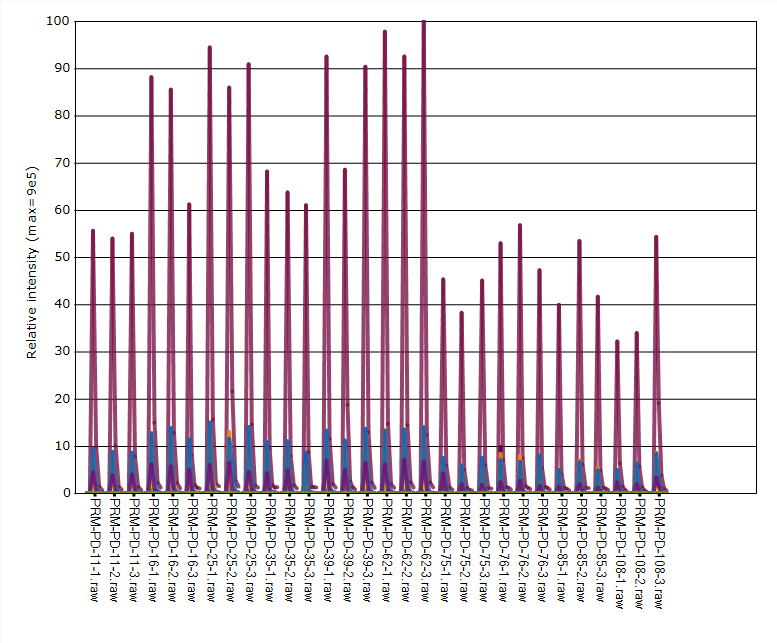
**

**F**

**E**

**
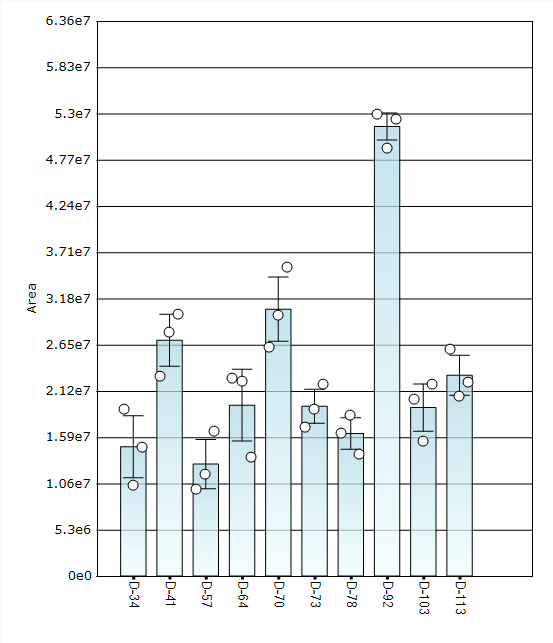

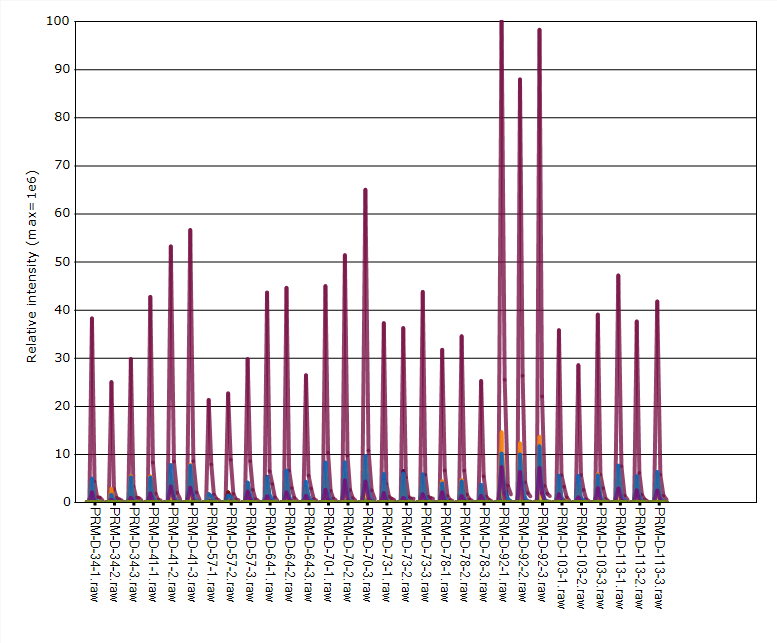
**

**H**

**G**

**
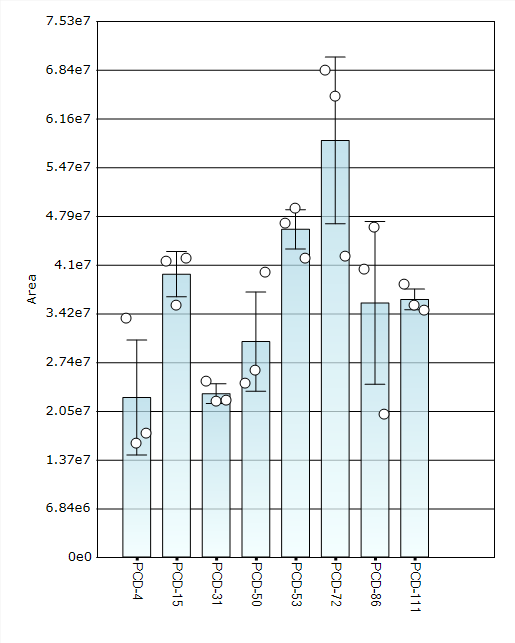

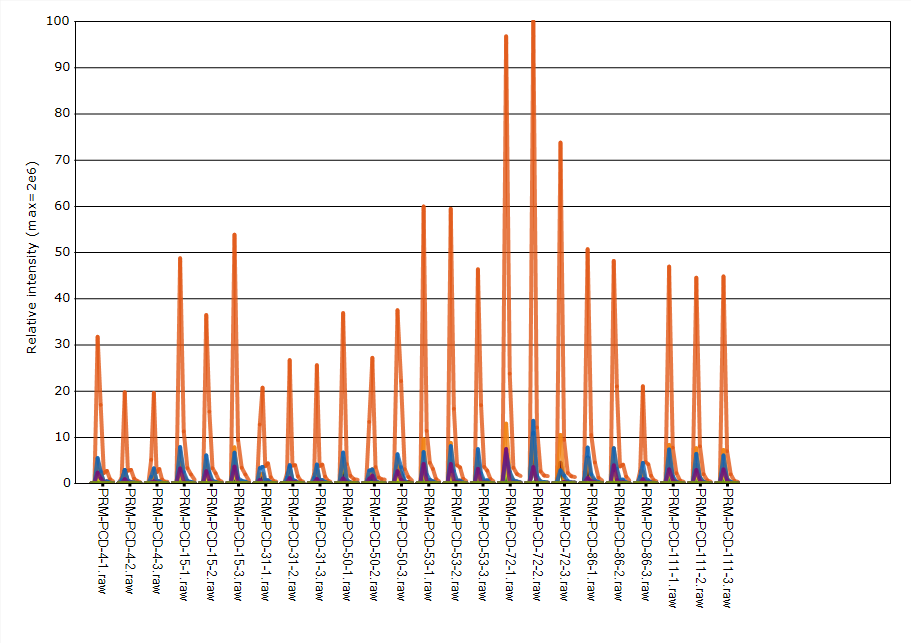
**

**J**

**I**

1. **KVADALTNAVAHVDDMPNALSALSDLHAHK-(DFL)LR:** K30-DFL (162.05282 Da), Charge: +6, Monoisotopic m/z: 593.47583 Da (-0.66 mmu/-1.12 ppm), MH+: 3555.81860 Da.

**B**

**
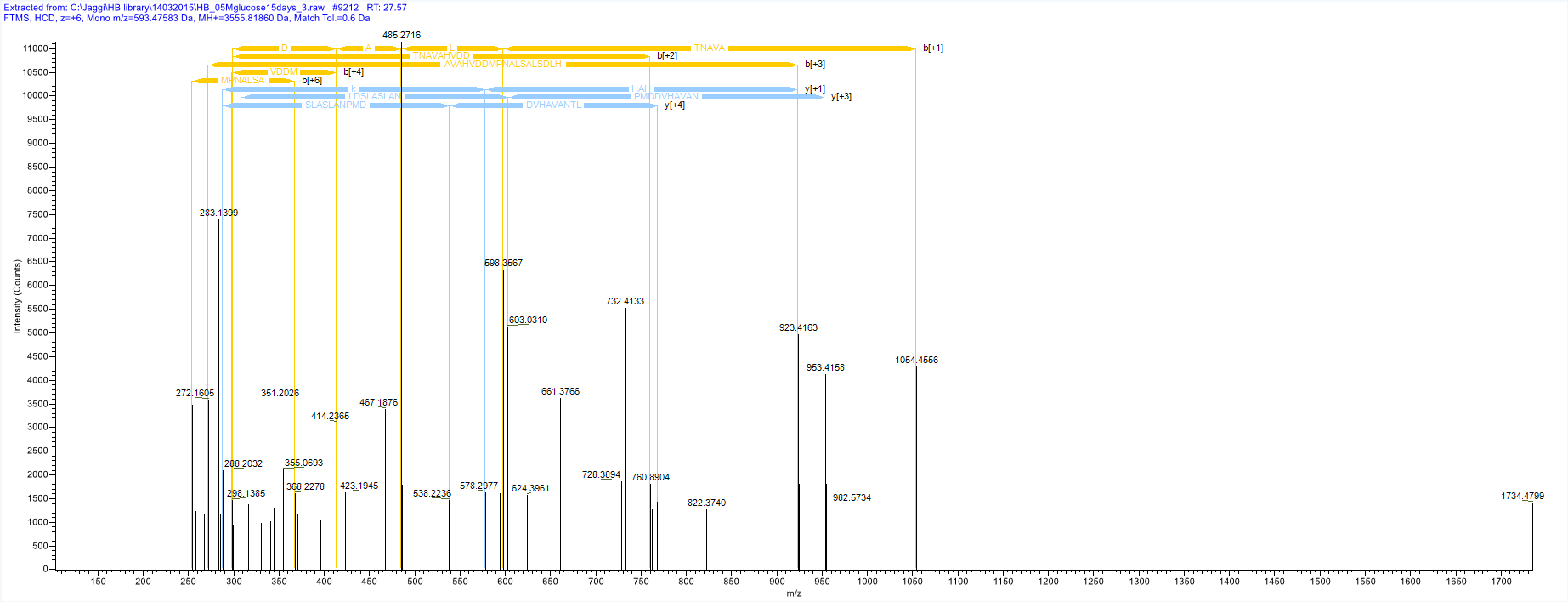

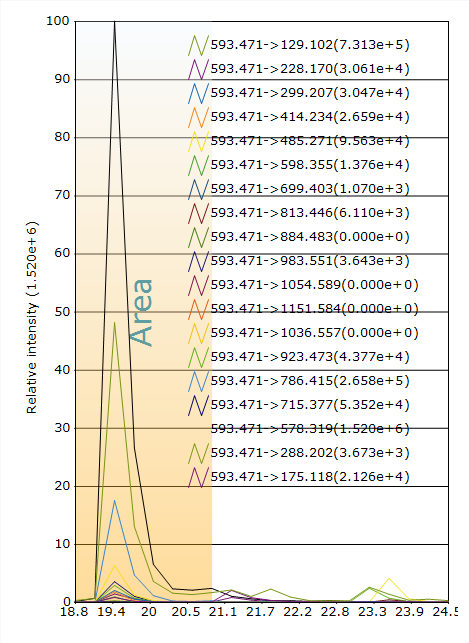
**

**A**

**
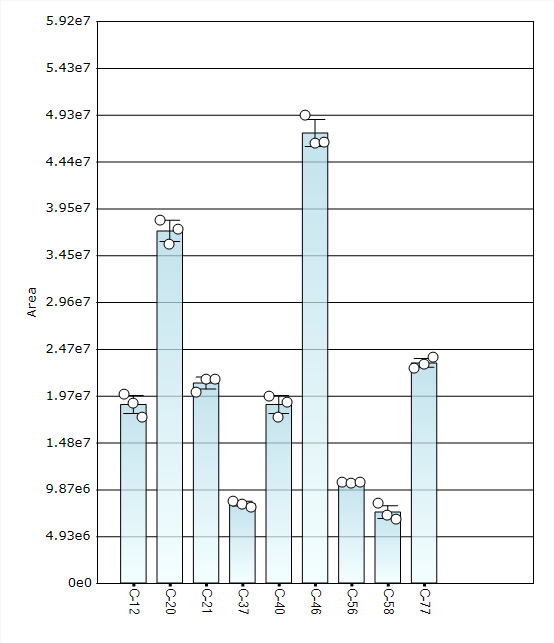

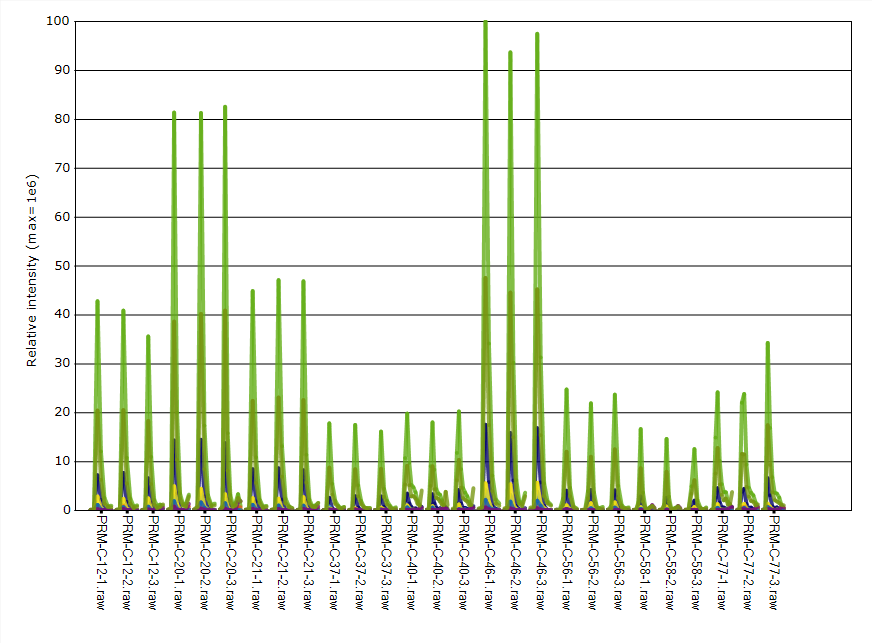
**

**D**

**C**

**
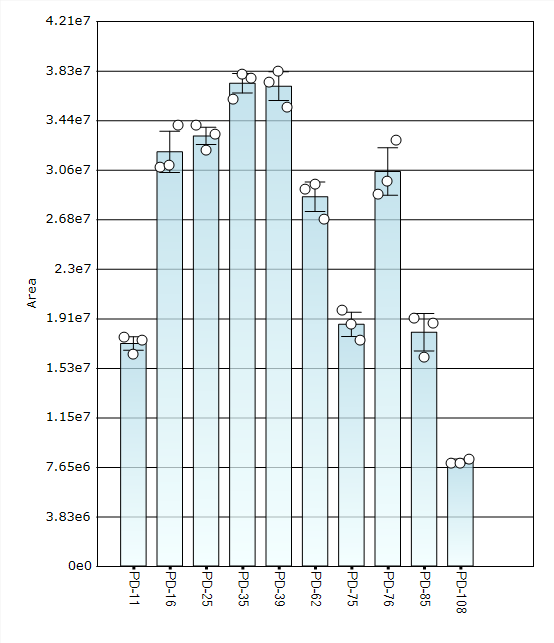

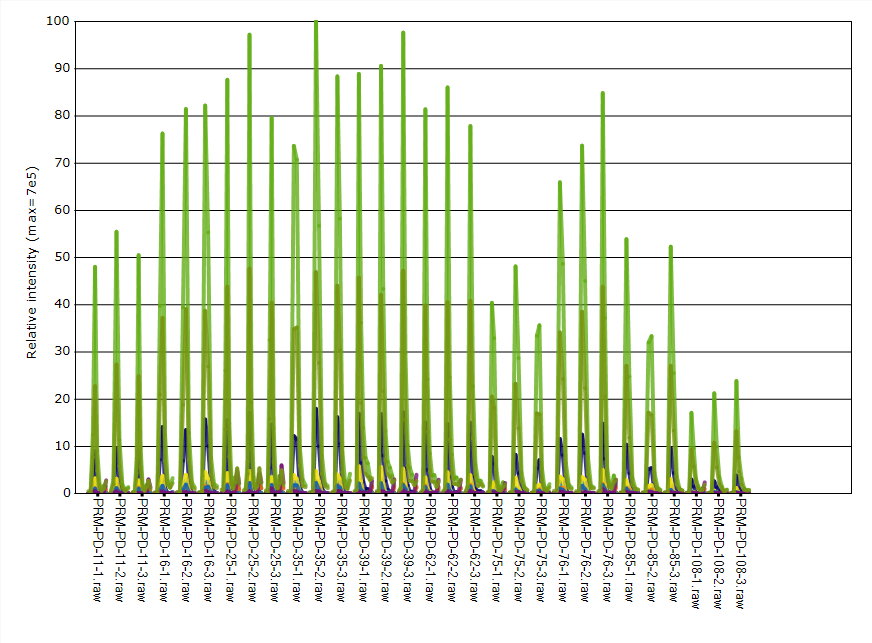
**

**E**

**F**

**
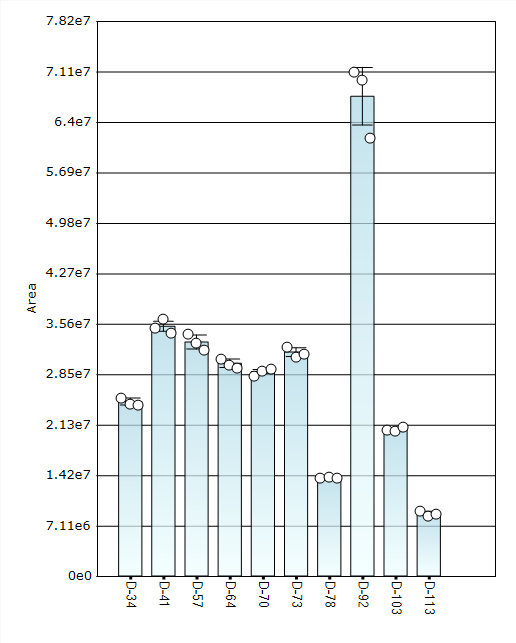

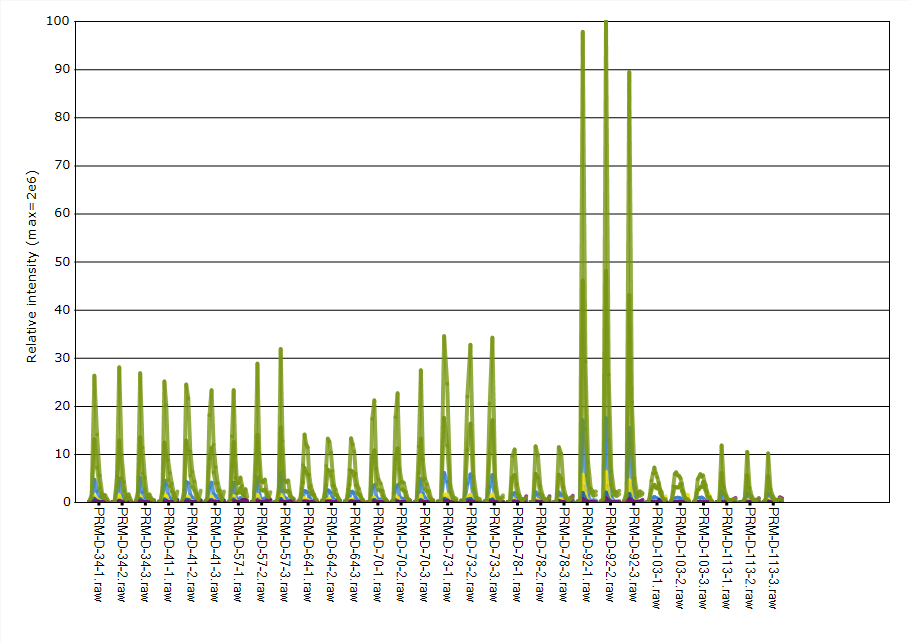
**

**H**

**G**

**
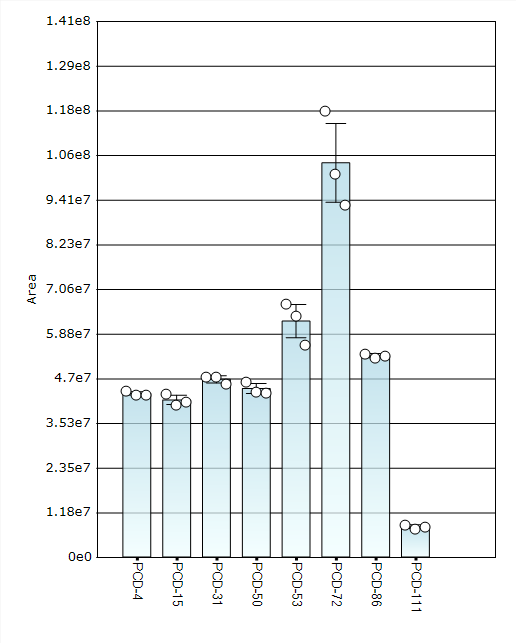

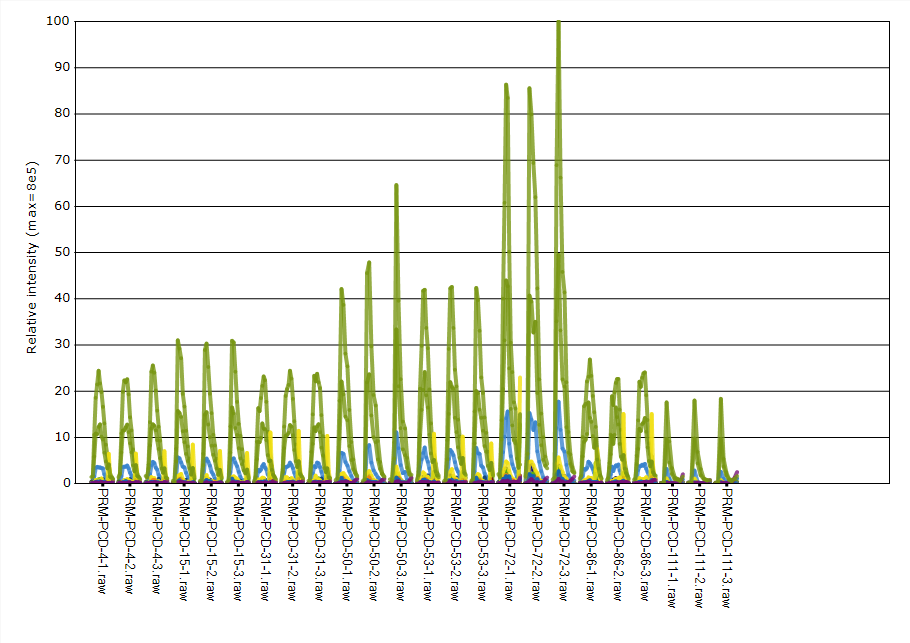
**

**J**

**I**

1. **MFLSFPTTK-(CML)TYFPHFDLSHGSAQVK-(CML)GHGK:** K9-CML(58.00548 Da), K25-CML(58.00548 Da) Charge: +5, Monoisotopic m/z: 677.13324 Da (+0.17 mmu/+0.26 ppm), MH+: 3381.63709 Da.

**
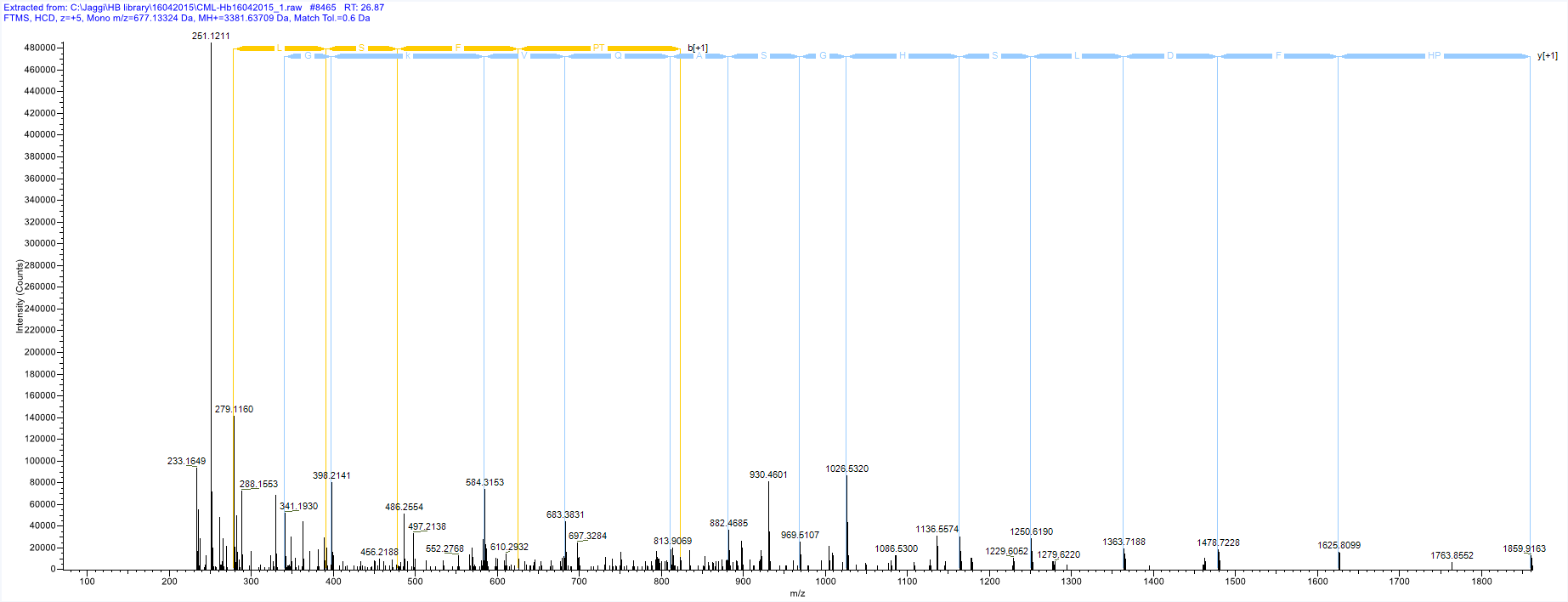

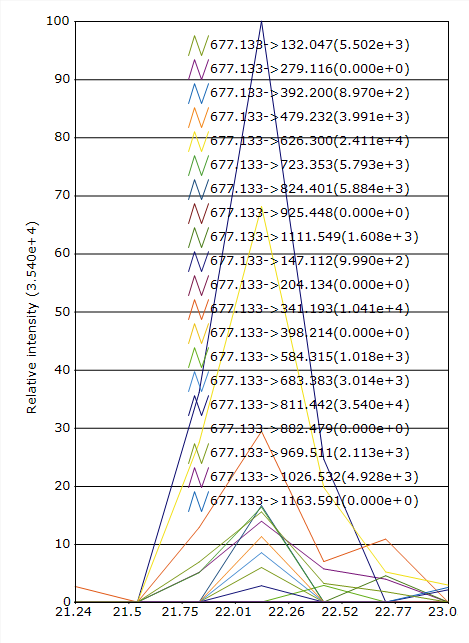
**

**B**

**A**

**
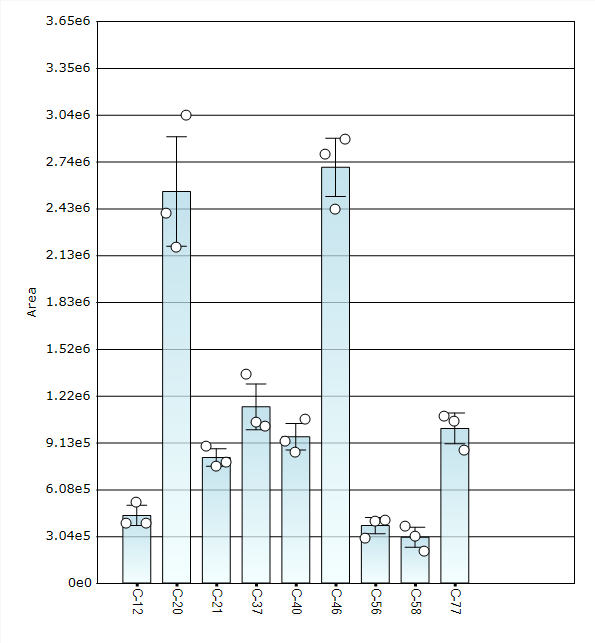

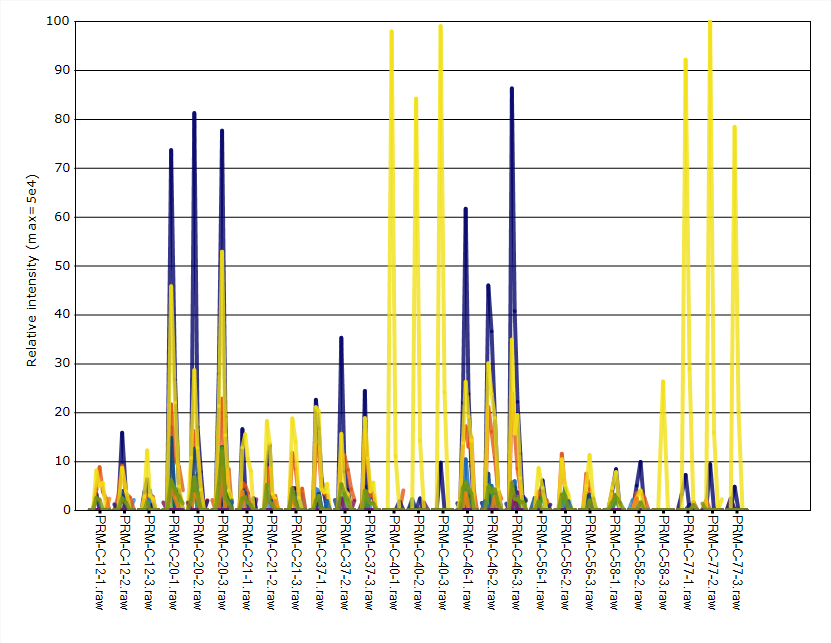
**

**D**

**C**

**
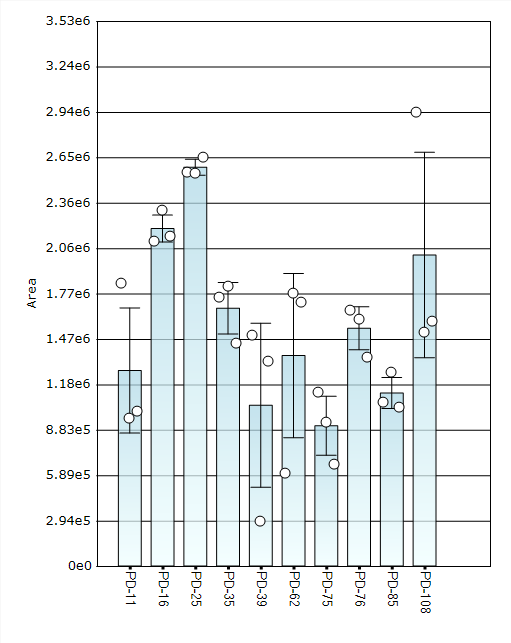

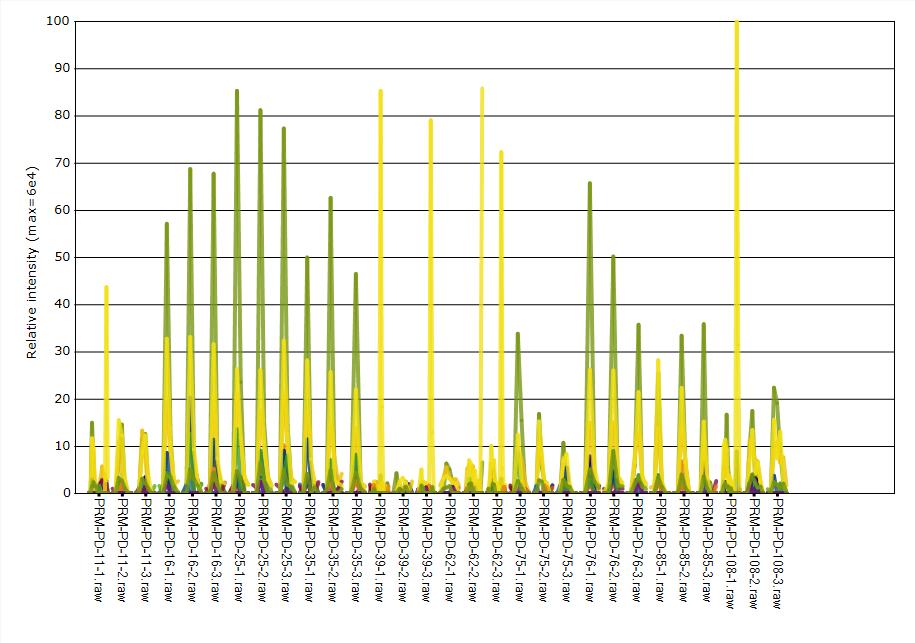
**

**F**

**E**

**
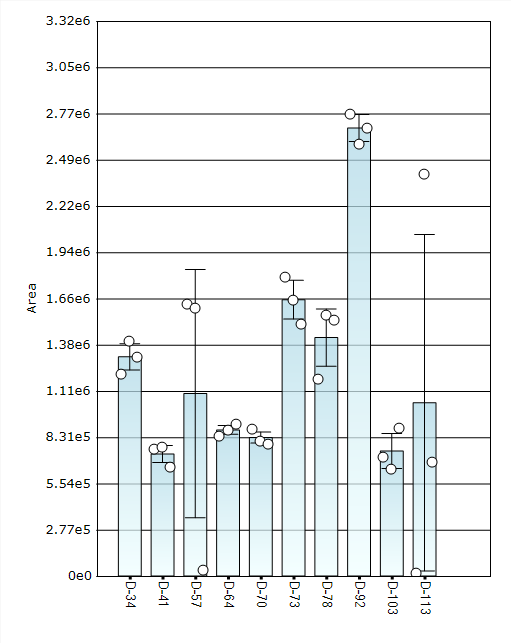

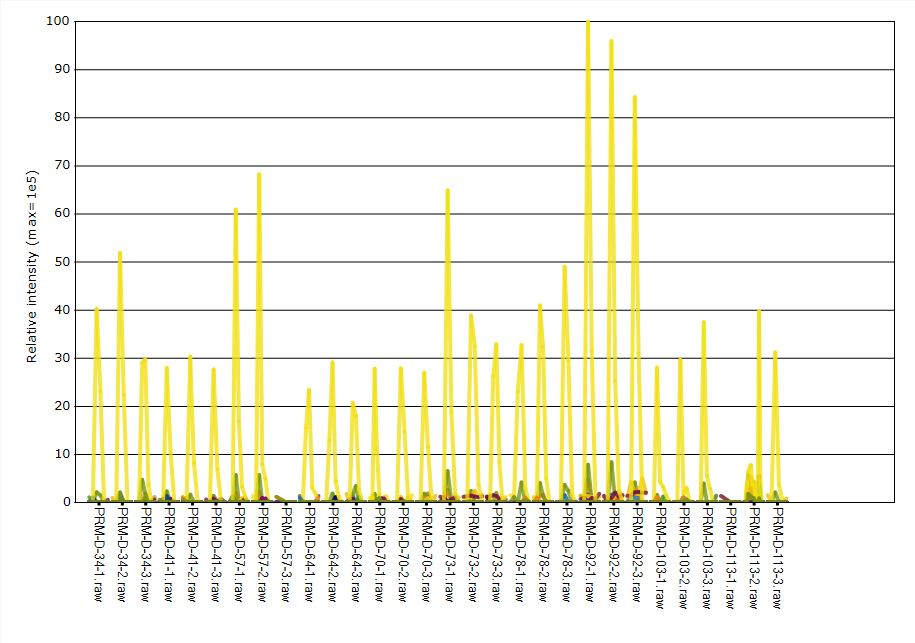
**

**H**

**G**

**
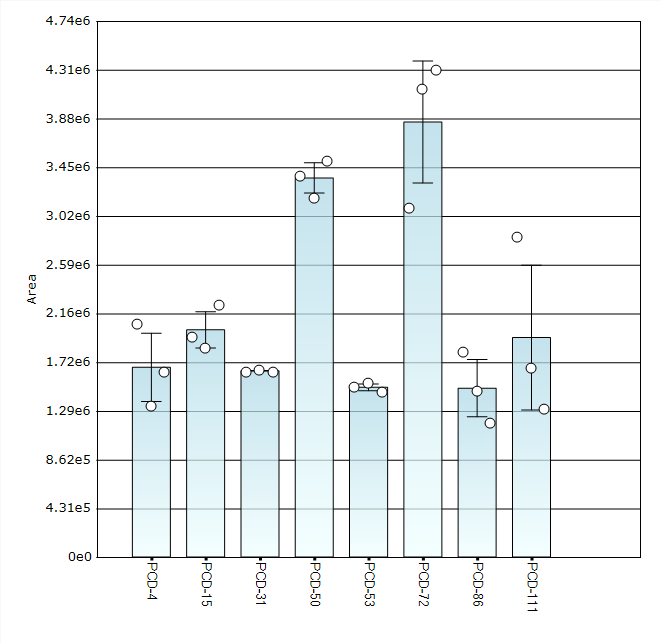

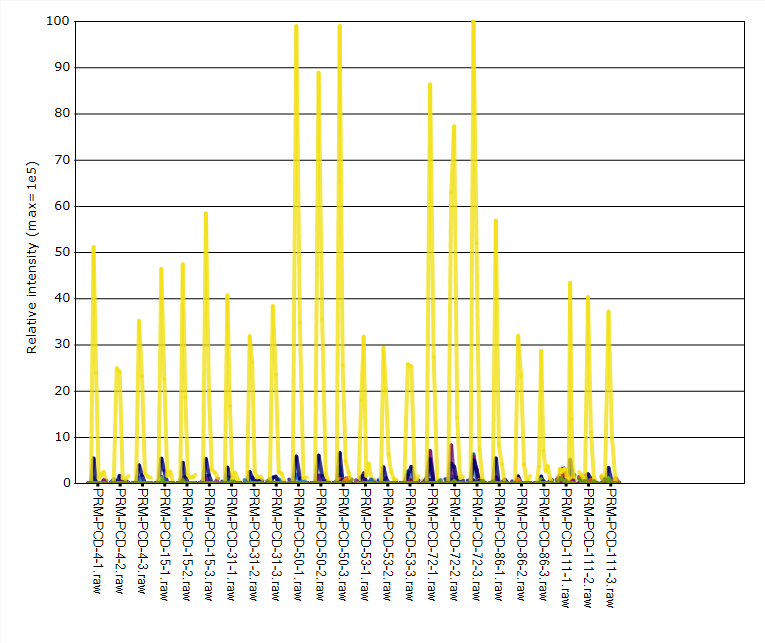
**

**J**

**I**

1. **K-(CML)VADALTNAVAHVDDM-(OXIDATION)PNALSALSDLHAHK-(CML)LR:** K1-CML(58.00548 Da), M16-OXD(15.99492 Da), K30-CML(58.00548 Da) Charge: +5, Monoisotopic m/z: 705.96106 Da (+2.25 mmu/+2.56 ppm), MH+: 3525.7761 Da.

**
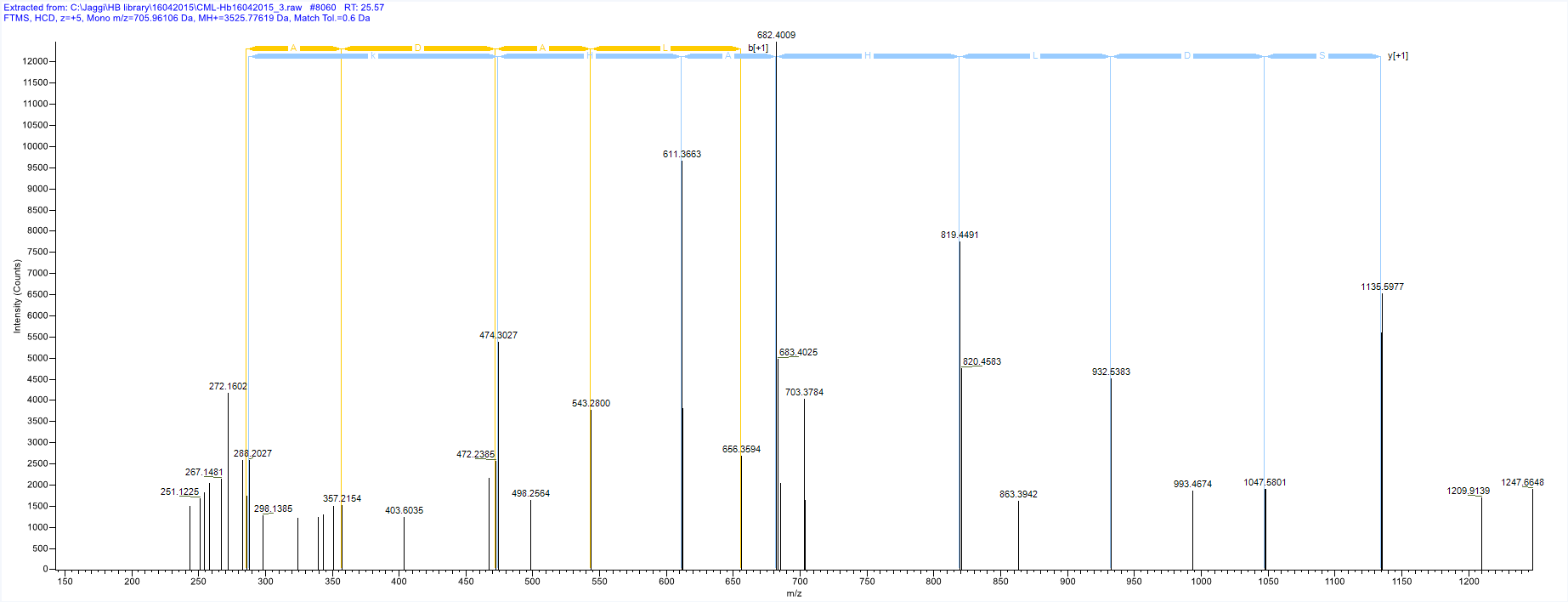

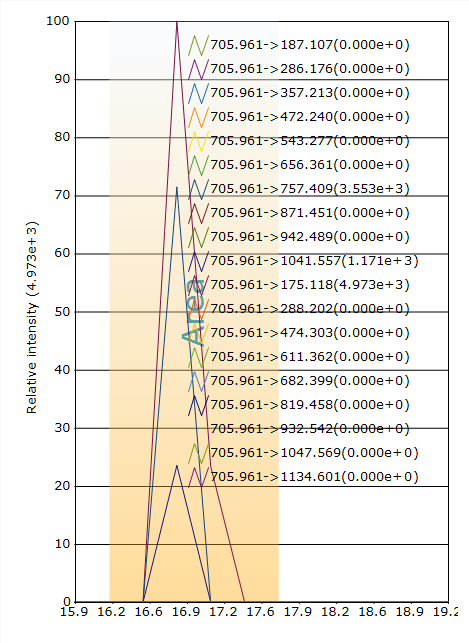
**

**B**

**A**

**
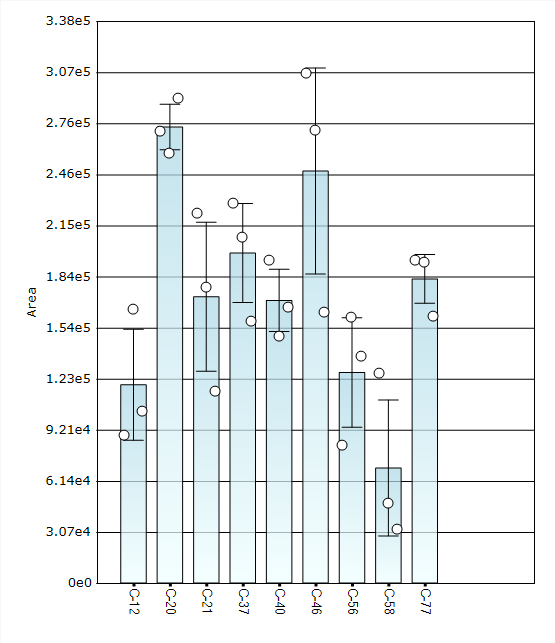

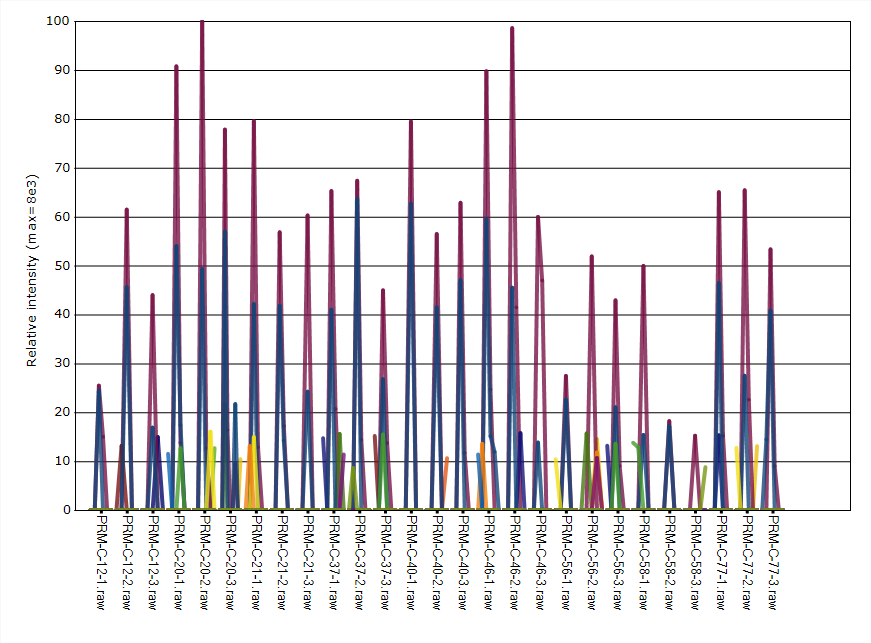
**

**D**

**C**

**
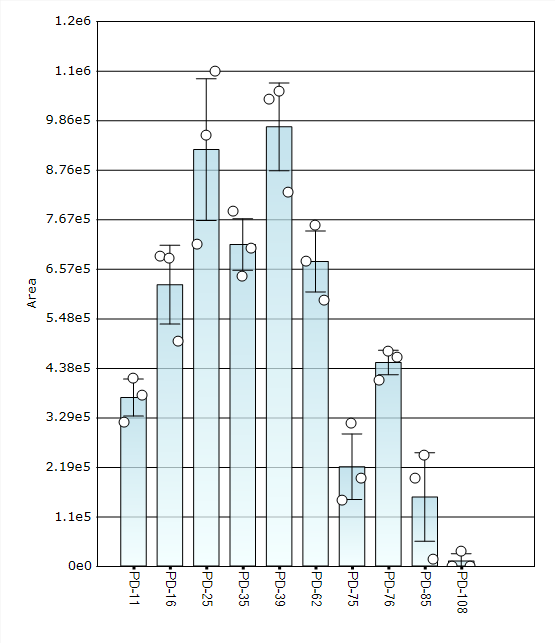

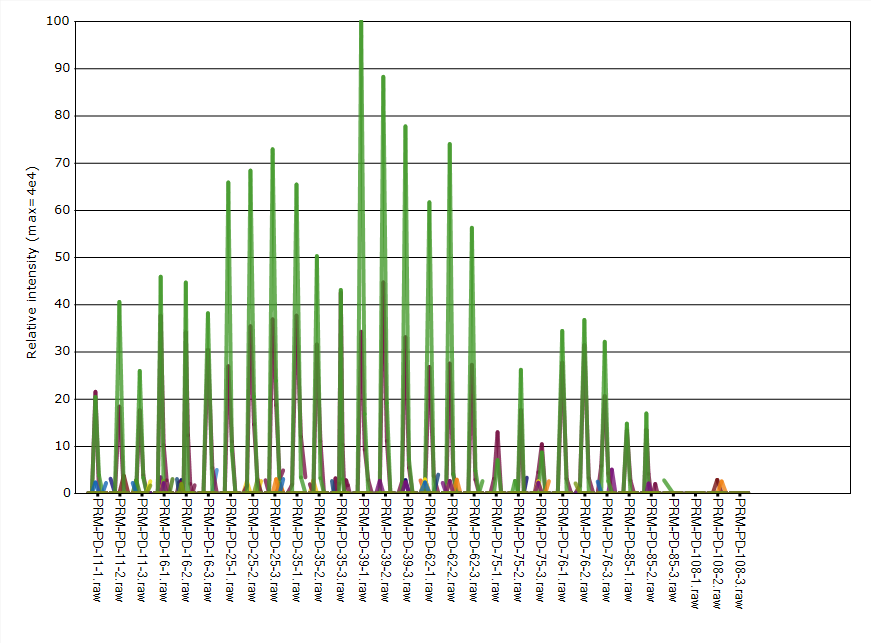
**

**F**

**E**

**
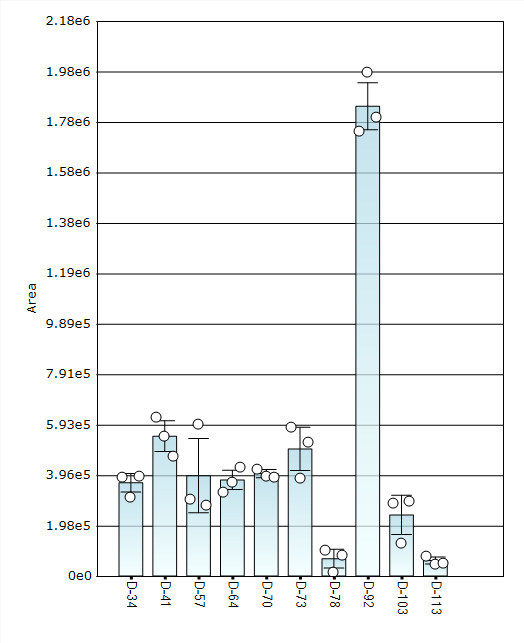

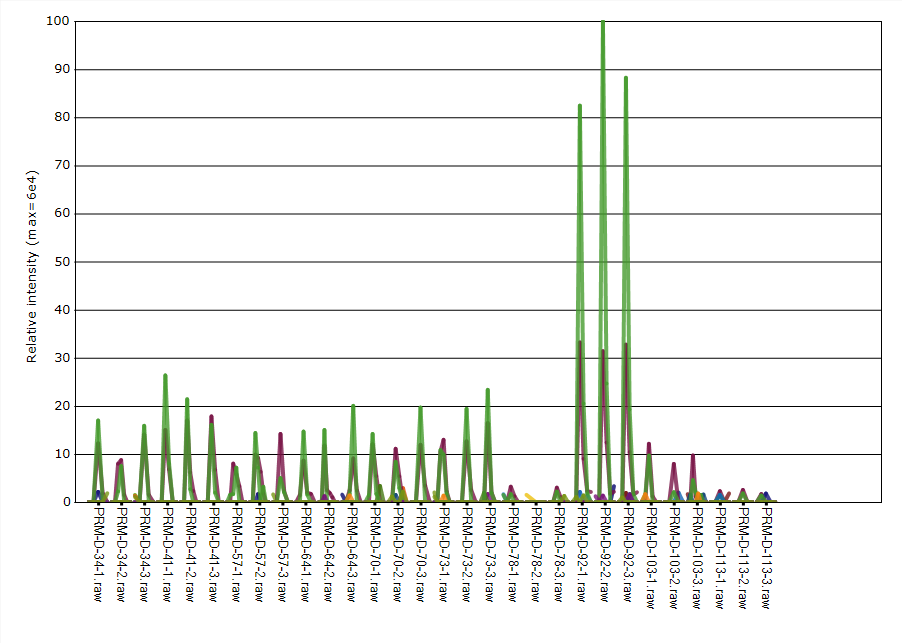
**

**H**

**G**

**
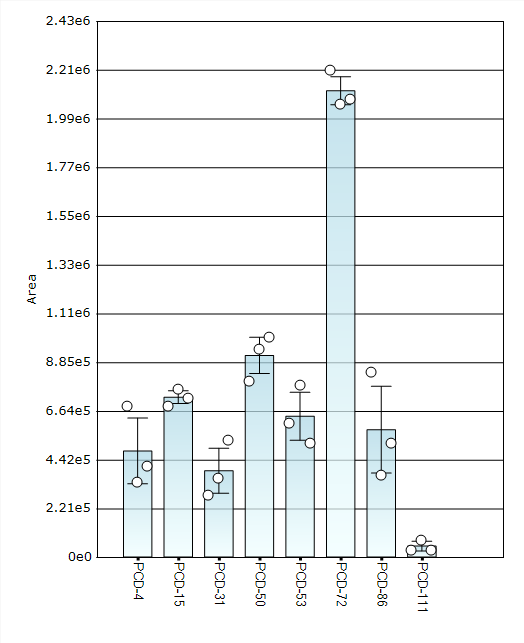

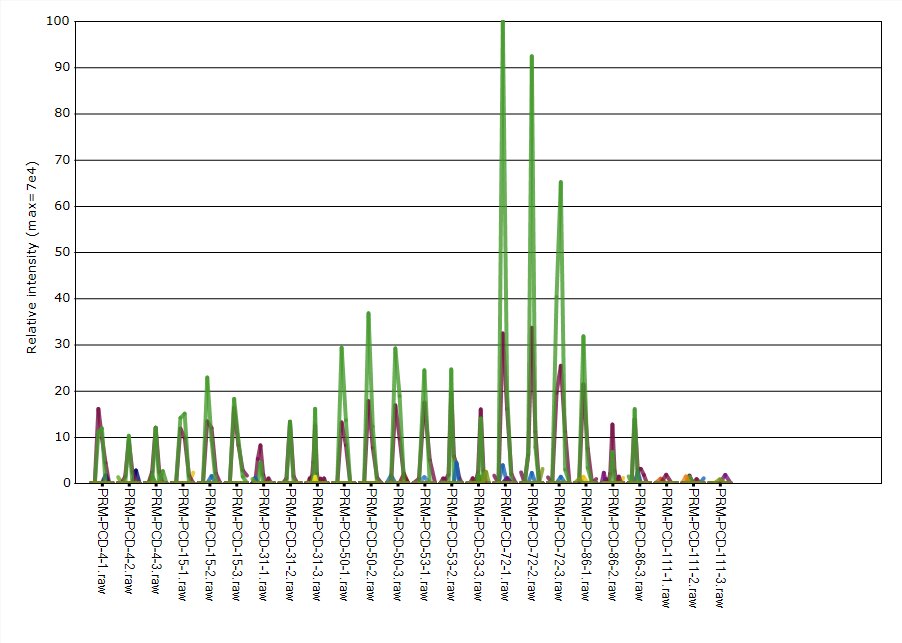
**

**J**

**I**

1. **VADALTNAVAHVDDMPNALSALSDLHAHK-(CML)LR:** K29-CML(58.00548 Da), Charge: +5, Monoisotopic m/z: 665.54303 Da (+1.16mmu/+1.74 ppm), MH+: 3323.68604 Da.

**
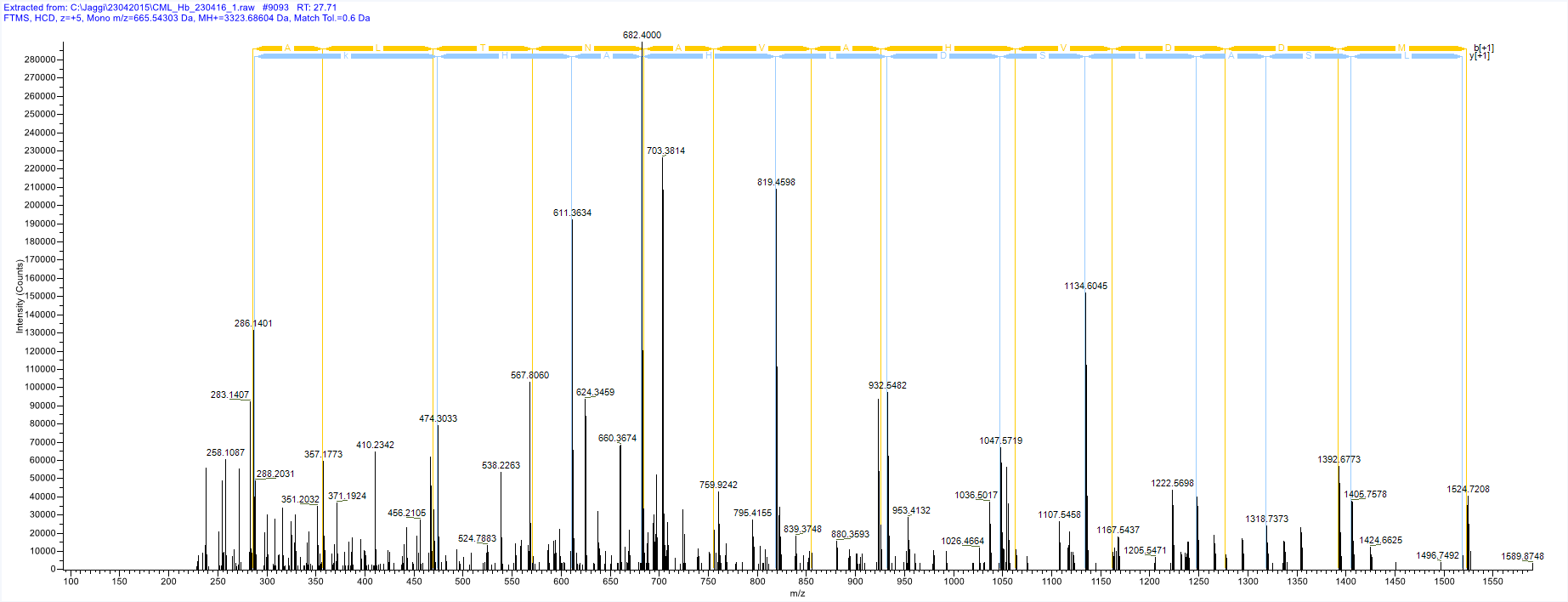

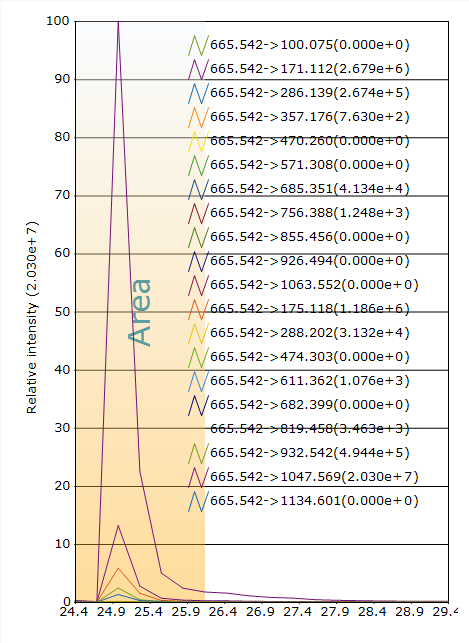
**

**B**

**A**

**
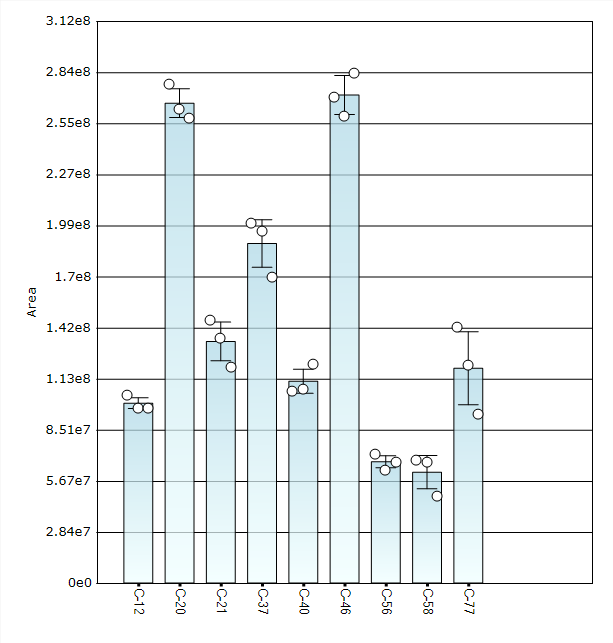

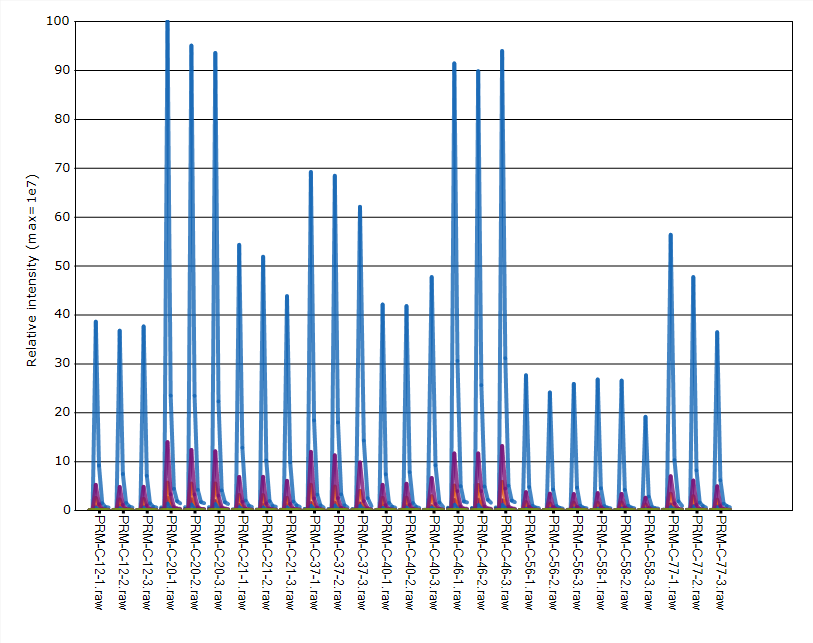
**

**D**

**C**

**
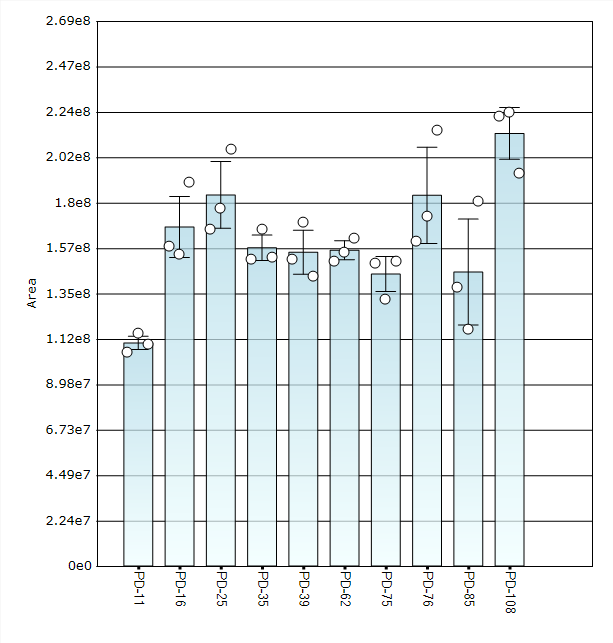

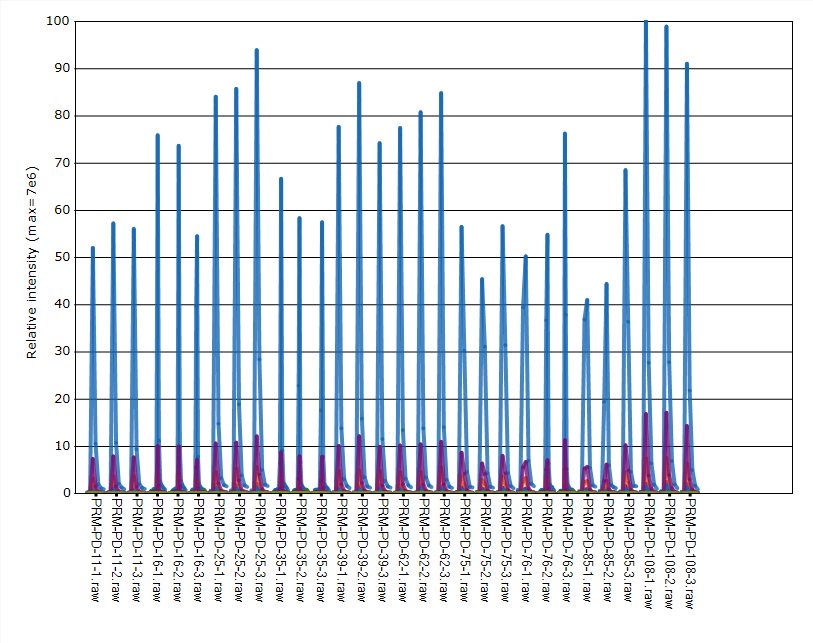
**

**F**

**E**

**
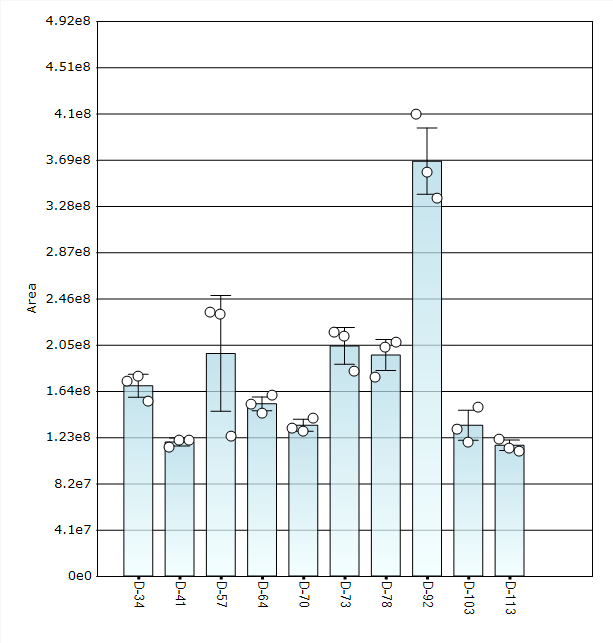

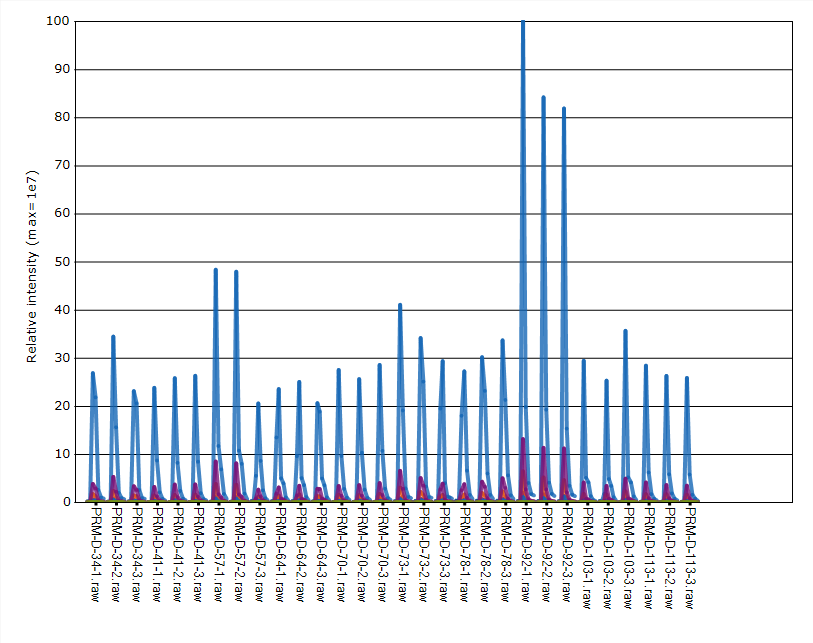
**

**H**

**G**

**
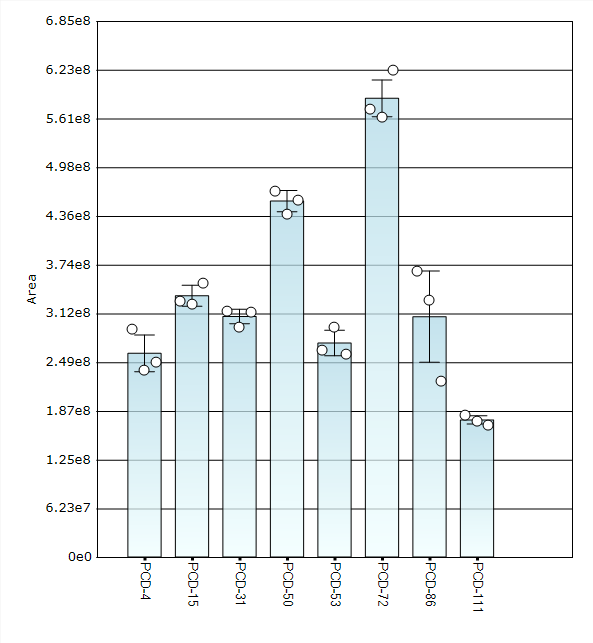

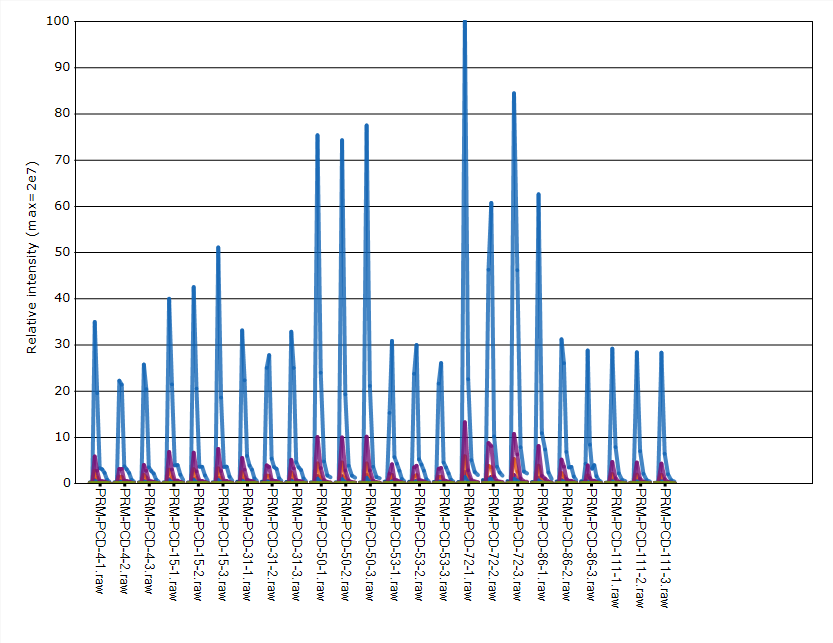
**

**J**

**I**

1. **VADALTNAVAHVDDM-(OXIDATION)PNALSALSDLHAHK-(CML)LR:** M15-OXD(15.99492 Da), K29-CML(58.00548 Da), Charge: +5, Monoisotopic m/z: 668.74042 Da (-0.44 mmu/-0.66 ppm), MH+: 3339.67298 Da.

**B**

**
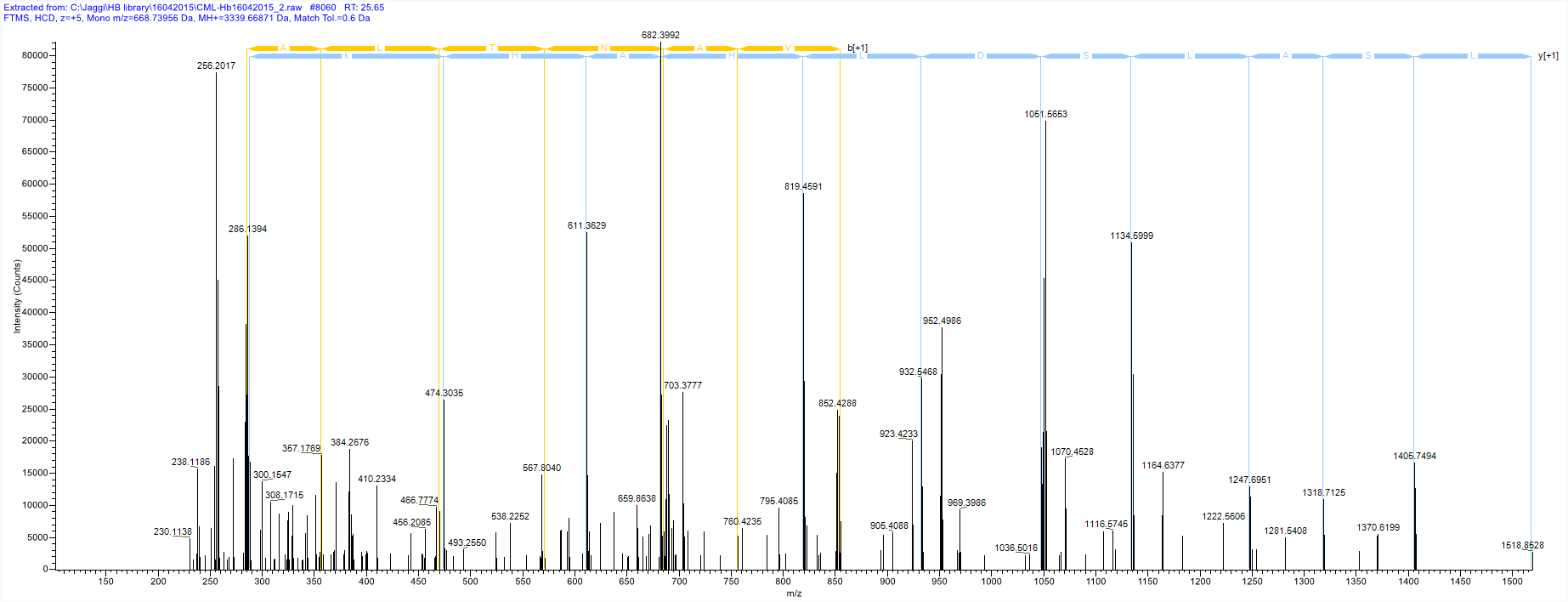

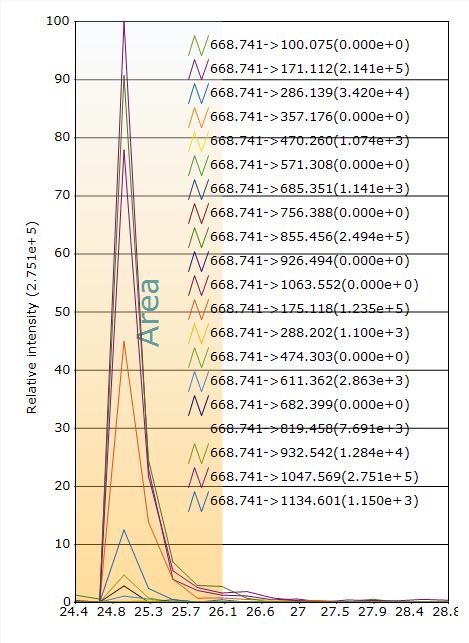
**

**A**

**
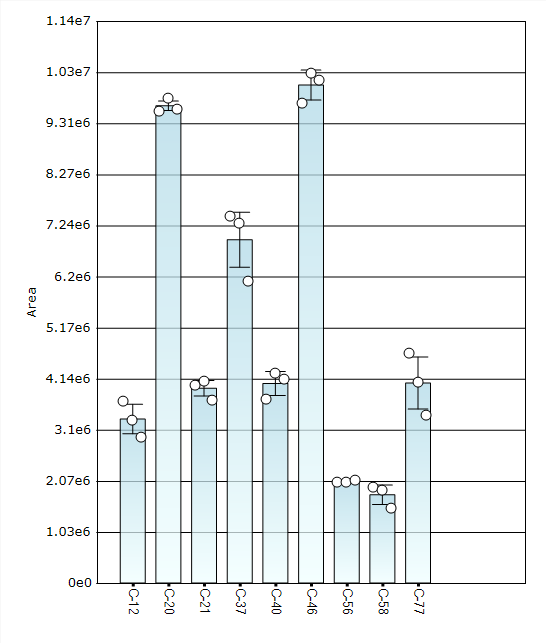

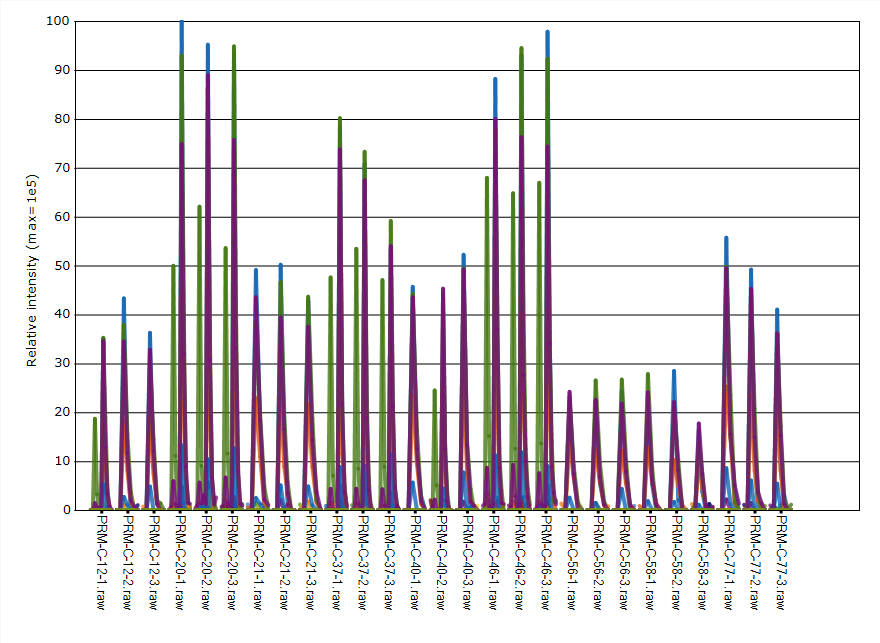
**

**D**

**C**

**
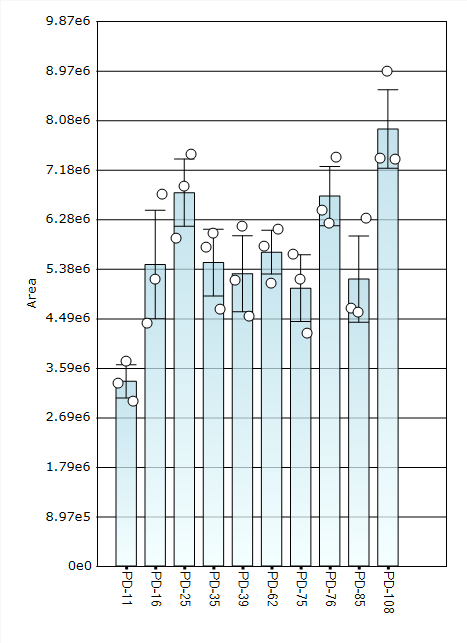

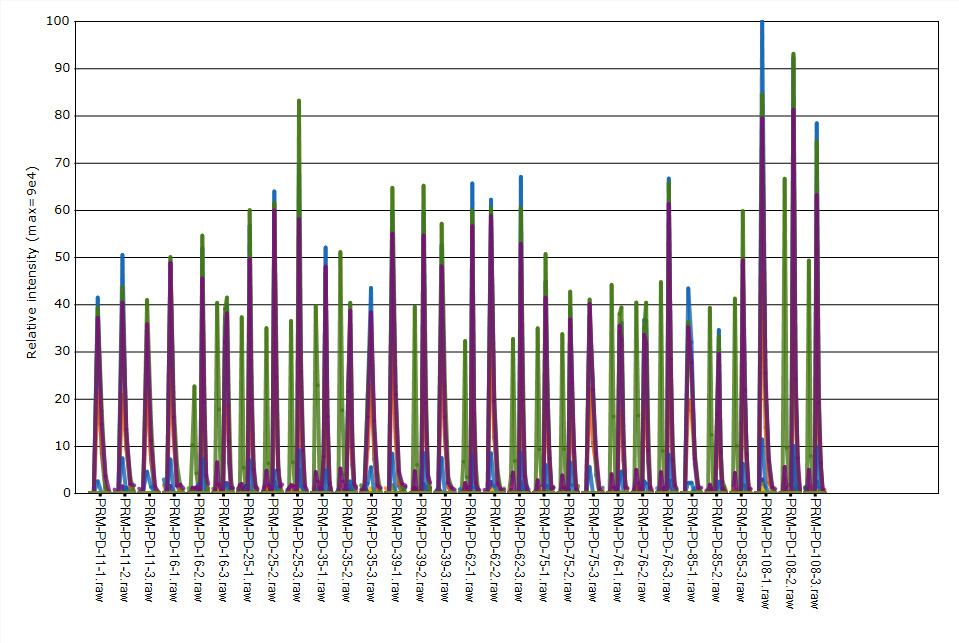
**

**F**

**E**

**
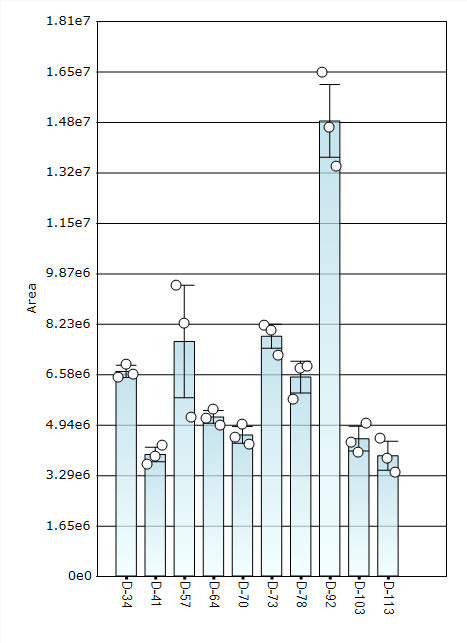

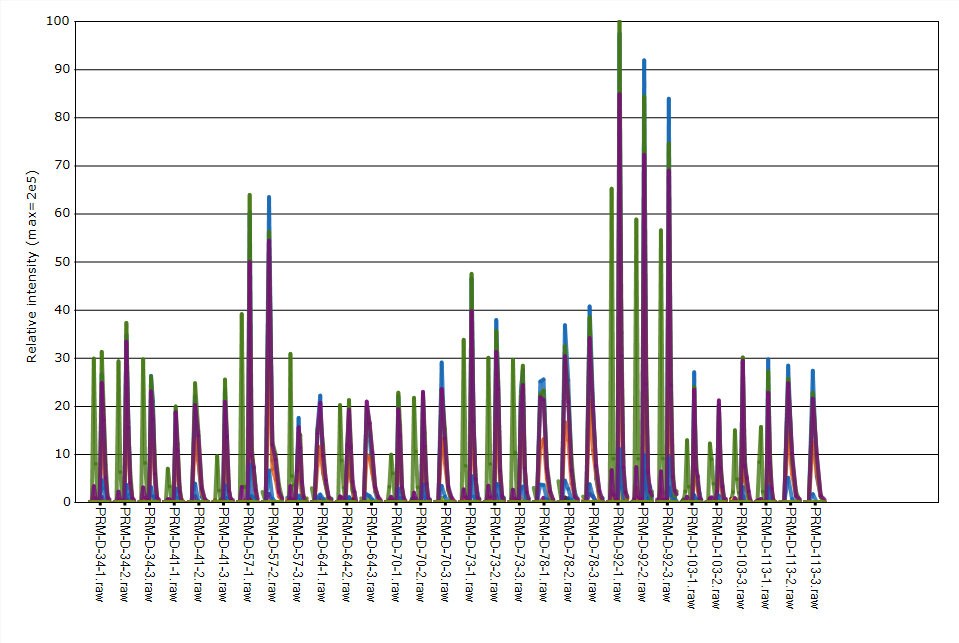
**

**H**

**G**

**
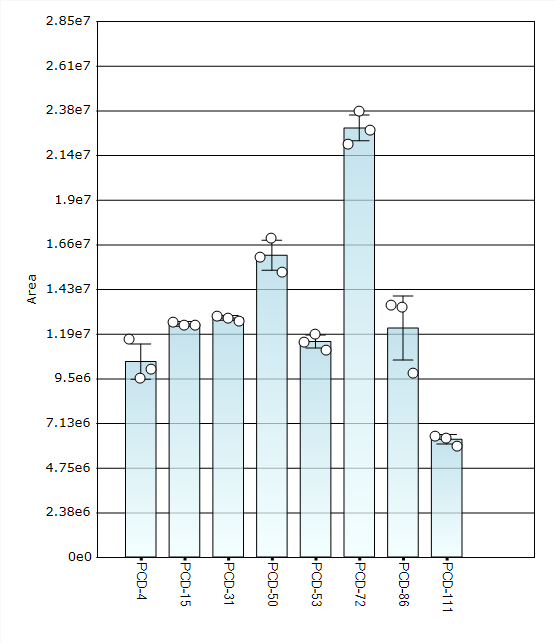

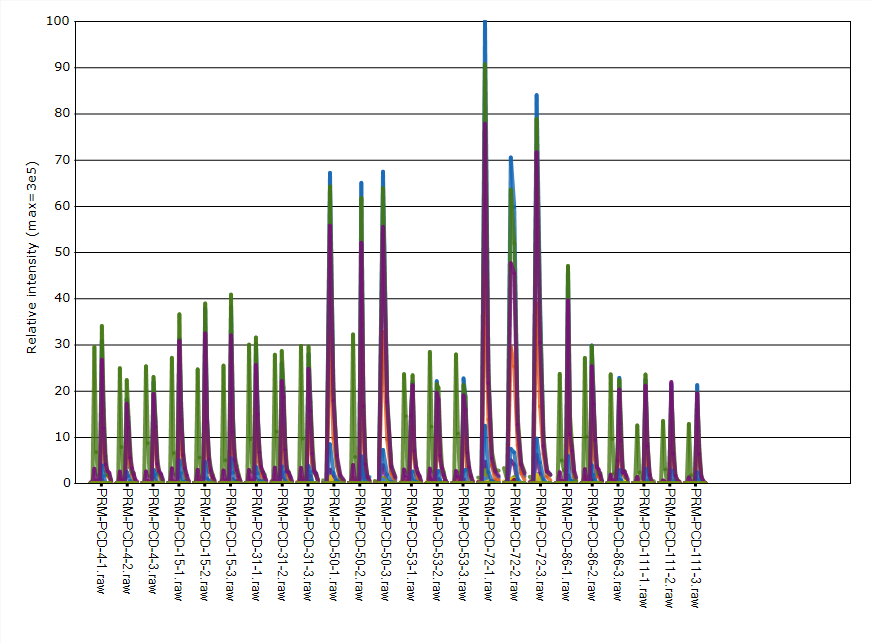
**

**J**

**I**

1. **FLASVSTVLTSK-(CML)YR:** K12-CML(58.00548 Da), Charge: +3, Monoisotopic m/z: 543.96686 Da (+0.44 mmu/+0.81 ppm), MH+: 1629.88602 Da.

**B**

**A**

**D**

**C**

**F**

**E**

**H**

**G**

**J**

**I**

1. **K-(CEL)VADALTNAVAHVDDMPNALSALSDLHAHK:** K1-CEL(72.02113 Da) Charge: +5, Monoisotopic m/z: 640.12512 Da (-1.84 mmu/-2.87 ppm), MH+: 3196.59650 Da.

**B**

**A**

**D**

**C**

**F**

**E**

**H**

**G**

**J**

**I**

1. **TNVK-(CEL)AAWGK:** K4-CEL(72.02113 Da), Charge: +2, Monoisotopic m/z: 523.78406 Da (-1.07 mmu/-2.04 ppm), MH+: 1046.56084 Da.

**B**

**A**

**D**

**C**

**F**

**E**

**H**

**G**

**J**

**I**

1. **VADALTNAVAHVDDMPNALSALSDLHAHK-(CEL)LR:** K29-CEL(72.02113 Da), Charge: +5, Monoisotopic m/z: 668.34460 Da (-0.4 mmu/-0.6 ppm), MH+: 3337.69392 Da.

**B**

**A**

**D**

**C**

**F**

**E**

**H**

**G**

**J**

**I**

1. **KVADALTNAVAHVDDMPNALSALSDLHAHK-(CEL)LR:** K30-CEL(72.02113 Da), Charge: +5, Monoisotopic m/z: 693.96552 Da (+1.52 mmu/+2.19 ppm), MH+: 3465.79847 Da.

**B**

**A**

**D**

**C**

**F**

**E**

**H**

**G**

**I**

**J**

1. **FLASVSTVLTSK-(CEL)YR:** K12-CEL(72.02113 Da), Charge: +3, Monoisotopic m/z: 548.63934 Da (+1.04 mmu/+1.9 ppm), MH+: 1643.90348 Da.

**B**

**A**

**D**

**C**

**F**

**E**

**H**

**G**

**J**

**I**

**Modified peptides of β-hemoglobin**

1. **V-(DFV)HLTPEEK:** V1-DFV (162.05282 Da), Charge: +2, Monoisotopic m/z: 557.78400 Da (-0.99 mmu/-1.77 ppm), MH+: 1114.56072 Da.

**D**

**C**

**B**

**A**

**J**

**I**

**H**

**G**

**F**

**E**

1. **V-(CMV)HLTPEEK:** V1-CMV(58.00548 Da), Charge: +2, Monoisotopic m/z: 505.75946 Da (-1.85 mmu/-3.66 ppm), MH+: 1010.51164 Da.

**D**

**C**

**B**

**A**

**J**

**I**

**H**

**G**

**F**

**E**

1. **V-(CEV)HLTPEEK:** V1-CEV(72.02113 Da), Charge: +2, Monoisotopic m/z: 512.76849 Da (-0.64 mmu/-1.25 ppm), MH+: 1024.52971 Da.

**B**

**A**

**D**

**C**

**F**

**E**

**H**

**G**

**J**

**I**

1. **V-(CMV)HLTPEEK-(CML)SAVTALWGK:** V1-CMV(58.00548 Da), K8-CML(58.00548 Da), Charge: +3, Monoisotopic m/z: 661.34637 Da (-1.18 mmu/-1.83 ppm), MH+: 1982.0245 Da.

**B**

**A**

**D**

**C**

**F**

**E**

**H**

**G**

**J**

**I**

1. **V-(CMV)HLTPEEK-(CML)SAVTALWGK-(CML)VNVDEVGGEALGR:** V1-CMV(58.00548 Da), K8-CML

(58.00548 Da), K17-CML(58.00548 Da) Charge: +3, Monoisotopic m/z: 1112.56604 Da (+2.67 mmu/+2.4 ppm), MH+: 3335.67666 Da.

**B**

**D**

**C**

**A**

**F**

**E**

**H**

**G**

**J**

**I**

1. **FFESFGDLSTPDAVM-(OXIDATION)GNPK-(CML)VK-(CML)AHGK:** M15-OXD(15.99492 Da), K19-CML(58.00548 Da), Charge: +4, Monoisotopic m/z: 703.58643 Da (-1.42 mmu/-2.02 ppm), MH+: 2811.3238 Da.

**B**

**A**

**D**

**C**

**F**

**E**

**H**

**G**

**J**

**I**

1. **AHGK-(CML)K-(CML)VLGAFSDGLAHLDNLK:** K4-CML(58.00548 Da), K5-CML(58.00548 Da), Charge: +4, Monoisotopic m/z: 577.55841 Da (+0.63 mmu/+1.1 ppm), MH+: 2307.21181 Da.

**B**

**A**

**D**

**C**

**F**

**E**

**H**

**G**

**J**

**I**

1. **K-(CML)VLGAFSDGLAHLDNLK-(CML)GTFATLSELHC-(CABD)DK:** K1-CML(58.00548 Da), K17-CML(58.00548 Da), C28-CABD(57.02146 Da) Charge: +5, Monoisotopic m/z: 675.53992 Da (-0.59 mmu/-0.88 ppm), MH+: 3373.67048 Da.

**B**

**A**

**D**

**C**

**F**

**E**

**H**

**G**

**J**

**I**

1. **LLGNVLVC-(CABD)VLAHHFGK-(CML)EFTPPVQAAYQK:** C8-CABD (57.02146 Da), K16-CML (58.00548 Da), Charge: +3, Monoisotopic m/z: 1065.56482 Da (-0.72 mmu/-0.68 ppm), MH+: 3194.67990 Da**.**

**B**

**A**

**D**

**C**

**F**

**E**

**H**

**G**

**J**

**I**

1. **VVAGVANALAHK-(CML)YH:** K12-CML(58.00548 Da), Charge: +3, Monoisotopic m/z: 754.404.27274 Da (+0.67 mmu/+1.34 ppm), MH+: 1507.80365 Da.

**B**

**A**

**D**

**C**

**F**

**E**

**H**

**G**

**J**

**I**

1. **FFESFGDLSTPDAVM-(OXD)GNPK-(CEL)VK:** M15-OXD(15.99492 Da), K19-CEL(72.02113 Da), Charge: +3, Monoisotopic m/z: 792.04761 Da (+0.33 mmu/+0.42 ppm), MH+: 2374.12827 Da.

**B**

**A**

**D**

**C**

**F**

**E**

**H**

**G**

**J**

**I**

1. **VVAGVANALAHK-(CEL)YH:** K12-CEL(72.02113 Da), Charge: +3, Monoisotopic m/z: 507.94379 Da (-0.16 mmu/-0.32 ppm), MH+: 1521.81681 Da.

**B**

**A**

**D**

**C**

**F**

**E**

**H**

**G**

**J**

**I**
