# Supplementary material for: Targeted quantification of N-1-(carboxymethyl) valine and N-1-(carboxyethyl) valine peptides of β-hemoglobin for better diagnostics in diabetes
Source: Clin Proteomics. 2016 Mar 29;13:7. doi: 10.1186/s12014-016-9108-y (PMC4812615; doi:10.1186/s12014-016-9108-y)
Supplement: Supplementary file 5 — 10.1186/s12014-016-9108-y Two way ANOVA followed by Bonferonnis posttests for fold change in AUCs of β-N-1-Val of hemoglobin. [file 12014_2016_9108_MOESM5_ESM.docx]

**Targeted quantification of N-1-(carboxymethyl) valine and N-1-(carboxyethyl) valine peptides of β-hemoglobin for better diagnostics in diabetes**

**Additional File 5:** Two way ANOVA followed by Bonferonnis posttests for fold change in AUCs of β-N-1-Val of hemoglobin.

| Parameter |  |  |  |  |
| --- | --- | --- | --- | --- |
| Table Analyzed | Data 1 |  |  |  |
| Two-way ANOVA |  |  |  |  |
| Source of Variation | % of total variation | P value |  |  |
| Interaction | 12.83 | 0.0004 |  |  |
| Row Factor | 8.26 | 0.0003 |  |  |
| Source of Variation | P value summary | Significant? |  |  |
| Interaction | *** | Yes |  |  |
| Time | *** | Yes |  |  |
| Row Factor | *** | Yes |  |  |
| Source of Variation | Df | Sum-of-squares | Mean square | F |
| Interaction | 6 | 27.91 | 4.652 | 4.608 |
| Time | 3 | 78.7 | 26.23 | 25.98 |
| Row Factor | 2 | 17.98 | 8.988 | 8.902 |
| Residual | 91 | 91.88 | 1.01 |  |
|  |  |  |  |  |
| **Bonferroni posttests** |  |  |  |  |
| **Control vs Prediabetes** | | | | |
| **Row Factor** | **Control** | **Prediabetes** | **Difference** | **95% CI of diff.** |
| DFV | 1 | 1.216 | 0.2158 | -1.241 to 1.672 |
| CMV | 1 | 1.289 | 0.2888 | -1.168 to 1.745 |
| CEV | 1 | 1.378 | 0.3783 | -1.041 to 1.798 |
| **Row Factor** | **Difference** | **t** | **P value** | **Summary** |
| DFV | 0.2158 | 0.4555 | P > 0.05 | ns |
| CMV | 0.2888 | 0.6097 | P > 0.05 | ns |
| CEV | 0.3783 | 0.8194 | P > 0.05 | ns |
| **Control vs Diabetes** | | | | |
| **Row Factor** | **Control** | **Diabetes** | **Difference** | **95% CI of diff.** |
| DFV | 1 | 1.419 | 0.4193 | -1.138 to 1.977 |
| CMV | 1 | 1.572 | 0.5721 | -0.8846 to 2.029 |
| CEV | 1 | 2.065 | 1.065 | -0.3920 to 2.521 |
| **Row Factor** | **Difference** | **t** | **P value** | **Summary** |
| DFV | 0.4193 | 0.828 | P > 0.05 | ns |
| CMV | 0.5721 | 1.208 | P > 0.05 | ns |
| CEV | 1.065 | 2.248 | P > 0.05 | ns |
| **Control vs Poorly Controlled Diabetes** | | | | |
| **Row Factor** | **Control** | **Poorly Controlled Diabetes** | **Difference** | **95% CI of diff.** |
| DFV | 1 | 2.069 | 1.069 | -0.4887 to 2.626 |
| CMV | 1 | 2.765 | 1.765 | 0.2634 to 3.266 |
| CEV | 1 | 5.175 | 4.175 | 2.674 to 5.677 |
| **Row Factor** | **Difference** | **t** | **P value** | **Summary** |
| DFV | 1.069 | 2.11 | P > 0.05 | ns |
| CMV | 1.765 | 3.615 | P<0.01 | ** |
| CEV | 4.175 | 8.551 | P<0.001 | *** |
| **Prediabetes vs Diabetes** | | | | |
| **Row Factor** | **Prediabetes** | **Diabetes** | **Difference** | **95% CI of diff.** |
| DFV | 1.216 | 1.419 | 0.2035 | -1.354 to 1.761 |
| CMV | 1.289 | 1.572 | 0.2832 | -1.173 to 1.740 |
| CEV | 1.378 | 2.065 | 0.6863 | -0.7334 to 2.106 |
| **Row Factor** | **Difference** | **t** | **P value** | **Summary** |
| DFV | 0.2035 | 0.4019 | P > 0.05 | ns |
| CMV | 0.2832 | 0.5979 | P > 0.05 | ns |
| CEV | 0.6863 | 1.487 | P > 0.05 | Ns |
| **Prediabetes vs Poorly Controlled Diabetes** | | | | |
| **Row Factor** | **Prediabetes** | **Poorly Controlled Diabetes** | **Difference** | **95% CI of diff.** |
| DFV | 1.216 | 2.069 | 0.8528 | -0.7045 to 2.410 |
| CMV | 1.289 | 2.765 | 1.476 | -0.02543 to 2.978 |
| CEV | 1.378 | 5.175 | 3.797 | 2.331 to 5.263 |
| **Row Factor** | **Difference** | **t** | **P value** | **Summary** |
| DFV | 0.8528 | 1.684 | P > 0.05 | ns |
| CMV | 1.476 | 3.023 | P<0.01 | ** |
| CEV | 3.797 | 7.966 | P<0.001 | *** |
| **Diabetes vs Poorly Controlled Diabetes** | | | | |
| **Row Factor** | **Diabetes** | **Poorly Controlled Diabetes** | **Difference** | **95% CI of diff.** |
| DFV | 1.419 | 2.069 | 0.6492 | -1.002 to 2.301 |
| CMV | 1.572 | 2.765 | 1.193 | -0.3087 to 2.694 |
| CEV | 2.065 | 5.175 | 3.111 | 1.609 to 4.612 |
| **Row Factor** | **Difference** | **t** | **P value** | **Summary** |
| DFV | 0.6492 | 1.209 | P > 0.05 | ns |
| CMV | 1.193 | 2.443 | P < 0.05 | * |
| CEV | 3.111 | 6.371 | P<0.001 | *** |
